# Ticagrelor Monotherapy or Aspirin Plus Prasugrel After Percutaneous Coronary Intervention: A Network Meta-Analysis

**DOI:** 10.1016/j.jacadv.2026.103298

**Published:** 2026-10-05

**Authors:** Michele Maremmani, Enrico Citerni, Ovidio De Filippo, Raffaele Piccolo, Alberto Menozzi, Carlos M. Campos, Francesco Costa, Fabrizio D’Ascenzo, Christian Templin, Davide Di Vece

**Affiliations:** a Department of Cardiology, Sant'Andrea Hospital, La Spezia, Italy; b Department of Cardiology, Campus Bio-Medico University Hospital, Rome, Italy; c Division of Cardiology, Department of Medical Sciences, AOU Città della Salute e della Scienza, University of Turin, Turin, Italy; d Department of Advanced Biomedical Sciences, University of Naples “Federico II”, Naples, Italy; e Heart Institute (InCor), Universidade de São Paulo (USP), São Paulo, Brazil; f Department of Biomedical and Dental Sciences and Morphological and Functional Imaging, University of Messina, Messina, Italy; A.O.U. Policlinic “G. Martino”, Messina, Italy; g Center for Molecular Cardiology, Schlieren Campus, University of Zurich, Zurich, Switzerland; h Department of Internal Medicine B, University of Medicine Greifswald, Greifswald, Germany; i Department of Internal Medicine, University of Genoa, Genoa, Italy

**Keywords:** diabetes mellitus, dual antiplatelet therapy, network meta-analysis, percutaneous coronary intervention, prasugrel, ticagrelor monotherapy

## Abstract

**Background:**

The 2025 American College of Cardiology/American Heart Association guidelines endorse de-escalation to ticagrelor monotherapy after percutaneous coronary intervention (PCI) for acute coronary syndromes (ACSs), whereas the 2023 European Society of Cardiology guidelines recommend 12-month dual antiplatelet therapy (DAPT) with aspirin plus prasugrel as the default strategy.

**Objectives:**

The objective of the study was to compare ischemic and bleeding outcomes between these strategies and to evaluate whether diabetes modifies their relative effects.

**Methods:**

Randomized controlled trials enrolling patients after PCI and evaluating de-escalation to ticagrelor monotherapy or 12-month DAPT (ticagrelor or prasugrel-based) were included. The primary efficacy and safety outcomes were major adverse cardiovascular or cerebrovascular events (MACCE) and major bleeding. Incidence rate ratios (IRRs) with 95% CIs were pooled using a frequentist random-effects network meta-analysis. Subgroup analyses were performed according to diabetes status.

**Results:**

Seven randomized controlled trials including 27,225 patients were included (mean age, 62 years; 23.2% women; 31.7% diabetes; 98.6% ACS). Ticagrelor monotherapy and aspirin plus prasugrel were compared indirectly through ticagrelor-based DAPT. Ticagrelor monotherapy showed similar MACCE compared with aspirin plus prasugrel (IRR: 1.09; 95% CI: 0.86-1.38), although a clinically meaningful increase in risk cannot be excluded (*P* for noninferiority = 0.33); major bleeding was lower with ticagrelor monotherapy (IRR: 0.54; 95% CI: 0.37-0.80). An interaction between P2Y_12_ inhibitor strategy and diabetes status was observed for MACCE (*P*int = 0.04).

**Conclusions:**

In this network meta-analysis of predominantly ACS patients undergoing PCI, ticagrelor monotherapy reduced major bleeding compared with 12 months of aspirin plus prasugrel, whereas it did not meet noninferiority for MACCE. Direct randomized comparisons are warranted. (Ticagrelor Monotherapy or Aspirin Plus Prasugrel After Percutaneous Coronary Intervention: A Network Meta-Analysis; CRD420261322968)

The optimal antiplatelet strategy after percutaneous coronary intervention (PCI) in patients with acute coronary syndromes (ACSs) remains debated. Twelve-month dual antiplatelet therapy (DAPT) has long been considered the standard of care, although this recommendation largely derives from trials comparing dual vs single antiplatelet therapy or different P2Y_12_ inhibitor potencies.^1^, ^2^, ^3^, ^4^, ^5^, ^6^, ^7^ More recently, randomized controlled trials (RCTs) and their individual patient-level meta-analysis showed that ticagrelor monotherapy after 1 to 3 months of DAPT was noninferior for major adverse cardiovascular or cerebrovascular events (MACCE) and reduced major bleeding compared with continued aspirin plus ticagrelor.^8^, ^9^, ^10^, ^11^, ^12^, ^13^ Accordingly, the 2025 American College of Cardiology/American Heart Association (ACC/AHA) guidelines endorse ticagrelor monotherapy after short DAPT as a Class I indication in ACS patients undergoing PCI.^14^ In contrast, the 2023 European Society of Cardiology (ESC) guidelines recommend 12-month DAPT as the default strategy and favor prasugrel in invasively managed patients, highlighting a clinically relevant divergence in guideline recommendations.^3^ Importantly, de-escalation to ticagrelor monotherapy has been evaluated against continuation of aspirin plus ticagrelor, a regimen shown in direct randomized comparisons to provide less ischemic protection than prasugrel-based DAPT.^15^^,^^16^ Whether ticagrelor monotherapy retains a favorable safety-efficacy trade-off when compared with 12 months of aspirin plus prasugrel remains uncertain. In addition, it remains unclear whether diabetes modifies the response to these strategies, including both the choice of P2Y_12_ inhibitor and the clinical consequences of early aspirin withdrawal. To address these gaps, we performed a frequentist network meta-analysis of RCTs comparing ticagrelor monotherapy with 12 months of aspirin plus prasugrel after PCI, including prespecified subgroup analyses according to diabetes status.

## Methods

### Study design

This systematic review and network meta-analyses were performed and reported following the Cochrane Collaboration Handbook for Systematic Reviews of Interventions and the Preferred Reporting Items for Systematic Reviews and Meta-analysis statement guidelines (Supplemental Methods 1 and 2).^17^^,^^18^ The meta-analysis protocol was registered at the International Prospective Register of Systematic Reviews (PROSPERO; CRD420261322968). Ethics committee approval was waived due to the nature of the study.

### Data source and search strategy

We systematically searched PubMed and EMBASE from inception to March 3, 2026. We also used backward snowballing to identify relevant texts from articles identified in the original search. The complete search strategies are provided in Supplemental Methods 3. Two authors (M.M. and E.C.) performed the systematic review independently, and disagreements were resolved by consensus. Study selection involved screening titles and abstracts, followed by a full-text evaluation of potentially eligible studies.

### Eligibility criteria

Studies were considered eligible for inclusion if they: 1) enrolled patients undergoing PCI with drug-eluting stent implantation; 2) randomly allocated patients to ticagrelor monotherapy after 1 to 3 months of DAPT or to standard 12-month DAPT (ticagrelor or prasugrel-based); and 3) reported at least 1 prespecified ischemic or bleeding endpoint of interest. Studies evaluating P2Y_12_ inhibitors other than ticagrelor or prasugrel, de-escalation to prasugrel monotherapy, mixed antiplatelet regimens, or immediate aspirin withdrawal without an initial period of DAPT were excluded, as were trials with substantial treatment crossover or protocol deviations likely to bias comparisons between P2Y_12_ inhibitors. In trials enrolling mixed populations, ACS-specific data sets were preferentially extracted when available. Subgroup analyses were used to obtain data for patients with and without diabetes but were not considered separate studies in the quantitative synthesis. No restrictions were applied regarding language, race, or ethnicity.

### Endpoints

The prespecified primary efficacy and safety end points were trial-defined MACCE and major bleeding, respectively. When multiple definitions were reported, those more closely aligned with the Academic Research Consortium recommendations were prioritized. Secondary end points included all-cause death, cardiac death, myocardial infarction (MI), stroke, and stent thrombosis (ST), according to each study’s definition. Effect estimates were derived from primary intention-to-treat analysis.

### Data extraction and quality assessment

Trial eligibility and data extraction were performed by 2 independent reviewers (M.M. and E.C.), and any disagreement was resolved by consensus. Study quality (performed by M.M. and E.C.) was evaluated using the Cochrane Risk of Bias Tool for RCTs.^19^ The certainty of evidence was assessed using the Grading of Recommendations Assessment, Development and Evaluation (GRADE) framework for network meta-analysis.^20^ Publication bias was assessed by visual inspection of comparison-adjusted funnel plots, whereas Egger test was not performed because of the limited number of included studies.^21^

### Statistical analysis

Pairwise and frequentist network meta-analyses were performed (March 2026). Effects were expressed as incidence rate ratios (IRRs) with 95% CIs, using patient-years as the denominator, and pooled as log-IRR using inverse-variance random-effects models with restricted maximum likelihood. An exploratory noninferiority analysis was performed for the primary efficacy endpoint comparing ticagrelor monotherapy and 12 months of aspirin plus prasugrel, using network-derived estimates and a margin of 1.15 derived from prior meta-analysis evaluating ticagrelor monotherapy vs 12 months of aspirin plus ticagrelor.^13^ Noninferiority was defined as the upper bound of the two-sided 95% CI below 1.15, corresponding to a one-sided α of 0.025. We produced network plots for each endpoint to visualize network geometry and node connectivity.^22^ Treatments were ranked using P-scores, with higher values indicating better overall performance. The numeric P-score values are nearly identical to the surface under the cumulative ranking curve.^23^ All statistical tests were 2-tailed with a significance threshold of *P* < 0.05, except for the noninferiority analysis (1-sided), for which the significance threshold was *P* < 0.025. Network heterogeneity was quantified using τ^2^ and I^2^ statistics. Prespecified subgroup analyses according to diabetes status were performed for the primary efficacy and safety endpoints for both pairwise and network meta-analyses. Effect modification was assessed using interaction tests. Sensitivity analyses included restriction to ACS-only trials, exclusion of predominantly East Asian populations, and analyses according to the timing of aspirin withdrawal, including: 1) restriction to trials with ≥3 months of DAPT before de-escalation; and 2) exclusion of trials with very early aspirin discontinuation (<1 month after PCI). Additional sensitivity analyses included the use of common-effect models when heterogeneity was low and pooling of HRs for time-to-event outcomes. Analyses were performed using R (version 4.5.2; R Foundation for Statistical Computing) with the “netmeta” and “dmetar” packages.^24^^,^^25^

## Results

### Study selection and baseline characteristics

Our systematic search identified 2,207 potential articles, of which 42 underwent full-text review (Supplemental Figure 1). Ultimately, five RCTs and 2 prespecified subgroup analyses of RCTs were included, totaling 27,225 patients (mean age, 62.0 years; 6,328 women [23.2%]; 8,642 with diabetes [31.7%]; 26,847 with ACS [98.6%]; 12,088 East Asian [44%]).^8^, ^9^, ^10^^,^^15^^,^^16^^,^^26^^,^^27^ All included data sets comprised patients with ACS, except for TUXEDO-2, which was included as the full randomized cohort because ACS-specific outcome data for the prespecified endpoints were not available; non-ACS patients accounted for 380 individuals (21.1% of TUXEDO-2 and 1.4% of the overall network). Five studies evaluated a strategy of short DAPT (1-3 months) followed by ticagrelor monotherapy compared with 12 months of aspirin plus ticagrelor; in T-PASS, aspirin was discontinued within 1 month after PCI, representing an early de-escalation approach.^8^, ^9^, ^10^^,^^26^^,^^27^ Two studies compared 12 months of aspirin plus ticagrelor with 12 months of aspirin plus prasugrel.^15^^,^^16^ Key characteristics of the included studies and populations are summarized in Tables 1 and 2. The duration of follow-up was 12 months in all studies included. The assumption of transitivity appeared broadly acceptable across the included studies. The mean age was identical across treatment nodes (63 years), whereas the prevalence of diabetes ranged from 28.2% to 45.6%, the proportion of patients presenting with ACS from 93% to 100%, and the proportion of East Asian patients from 31% to 49%. Detailed study-level characteristics summarized by treatment node are reported in Supplemental Table 1.

**Table 1:** Key Characteristics of Randomized Trials Included in the Meta-Analysis

Included Studies	Study Type (Years)	Inclusion Criteria	Sample Size (Intervention/Control)	Timing of Randomization	Timing of DAPT Interruption	Primary Efficacy	Primary Safety	Timing of the Endpoints
De-escalation to ticagrelor monotherapy after short DAPT vs 12 months ticagrelor-based DAPT								
GLOBAL-LEADERS-ACS (Tomaniak et al)^26^	Nonprespecified, subgroup analysis of multicenter, open-label, RCT (GLOBAL-LEADERS, 2013-2015)	ACS undergoing PCI with BP-BES	3,750/3,737	Pre-PCI	1 mo	All-cause death or new Q-wave MI	BARC 3 or 5	12 mo
TICO (Kim et al)^10^	Multicenter, open-label RCT (2015-2018)	ACS after successful PCI with BP-SES	1,527/1,529	Post-PCI	3 mo	NACE (major bleeding or MACCE)	TIMI major bleeding	12 mo
TWILIGHT-ACS (Baber et al)^27^	Prespecified subgroup analysis of multicenter, double-blind, placebo-controlled, RCT (TWILIGHT, 2015-2017)	High-risk ACS undergoing PCI with DES and event free after 3 mo DAPT	2,273/2,341	3 mo post-PCI	3 mo	All-cause death, MI, or stroke	BARC 2, 3, or 5	12 mo
T-PASS (Hong et al)^9^	Multicenter, open-label, noninferiority RCT (2019-2022)	ACS after successful PCI with BP-SES	1,426/1,424	Post-PCI	<1 mo	All-cause death, MI, ST, stroke, or major bleeding	BARC 3 or 5	12 mo
ULTIMATE-DAPT (Ge et al)^8^	Multicenter, placebo-controlled, double-blind RCT (2019-2022)	ACS event-free after 1-mo DAPT (IVUS-guided PCI)	1,700/1,700	1 mo post-PCI	1 mo	Cardiac death, MI, stroke, ST, and clinically driven TVR	BARC 2, 3, or 5	12 mo
12-month ticagrelor-based vs prasugrel-based DAPT								
ISAR-REACT 5 (Schupke et al)^15^	Multicenter, open-label RCT (2013-2018)	ACS planned for invasive strategy; DES used in 90.0% of patients undergoing PCI	2,012/2,006	Pre-PCI	12 mo	All-cause death, MI, or stroke	BARC 3, 4, or 5	12 mo
TUXEDO-2 (Bangalore et al)^16^	Multicenter, open-label, 2×2 factorial RCT (2020-2024)	Patients with T2DM and multivessel CAD undergoing PCI with BP-SES or DP-EES	901/899	Post-CA	12 mo	NACE (All-cause death, MI, stroke, or major bleeding)	BARC 3, 4, or 5	12 mo

ACS = acute coronary syndrome; BARC = Bleeding Academic Research Consortium; BP-BES = biodegradable polymer biolimus-eluting stent; BP-SES = biodegradable polymer sirolimus-eluting stent; CA = coronary angiography; CAD = coronary artery disease; DAPT = dual antiplatelet therapy; DES = drug-eluting stent; DP-EES = durable polymer everolimus-eluting stent; IVUS = intravascular ultrasound; MACCE = major adverse cardiovascular or cerebrovascular events; MI = myocardial infarction; NACE = net adverse clinical events; PCI = percutaneous coronary intervention; RCT = randomized controlled trial; ST = stent thrombosis; TVR = target vessel revascularization; T2DM = type 2 diabetes mellitus.

**Table 2:** Baseline Characteristics of the Trial Populations

Included Studies (Sample Size)	Global Leaders ACS, 2019^26^ (n = 7,487)	TICO, 2020^10^ (n = 3,056)	ULTIMATE-DAPT, 2024^8^ (n = 3,400)	T-PASS, 2023^9^ (n = 2,850)	TWILIGHT-ACS, 2020^27^ (n = 4,614)	ISAR-REACT 5, 2019^15^ (n = 4,018)	TUXEDO-2, 2026^16^ (n = 1,800)
Mean age, y	65	61	62	61	64	64	60
Female, n (%)	1,724 (23.1)	628 (20.5)	879 (25.9)	476 (16.7)	1,161 (25.2)	956 (23.8)	504 (28.0)
Hypertension, n (%)	5,083 (68.3)	1,541 (50.4)	2,121 (62.4)	1,348 (47.3)	3,111 (67.4)	2,816 (70.1)	1,084 (60.2)
Diabetes, n (%)	1,604 (21.5)	835 (27.3)	1,075 (31.6)	830 (29.1)	1,614 (35.0)	892 (22.2)	1,792 (99.6)
Dyslipidemia, n (%)	4,389 (61.4)	1,846 (60.4)	2,335 (68.7)	2,106 (73.4)	NA	2,341 (58.3)	1,497 (83.2)
Current smoker, n (%)	2,543 (33.9)	1,142 (37.4)	968 (28.5)	1,094 (38.4)	1,150 (24.9)	1,349 (33.6)	286 (15.9)
CKD^a^, n (%)	967 (12.9)	620 (20.3)	248 (7.3)	222 (7.8)	662 (14.3)	176 (4.4)	47 (2.6)
East Asian^b^, n (%)	142 (1.9)	3,056 (100)	2,995 (88)	2,850 (100)	1,387 (30)	0 (0)	0 (0)
ACS, n (%)	7,487 (100)	3,056 (100)	3,400 (100)	2,850 (100)	4,614 (100)	4,018 (100)	1,420 (78.9)
STEMI, n (%)	2,092 (27.9)	1,103 (36.1)	948 (27.9)	1,150 (40.4)	0 (0)	1,653 (41.1)	452 (25.1)
NSTEMI, n (%)	3,373 (45.1)	1,027 (33.6)	1,076 (31.6)	992 (34.8)	4,614 (100)	1,855 (46.2)	607 (33.7)
Unstable angina, n (%)	2,022 (27.0)	926 (30.3)	1,376 (40.5)	708 (24.8)	0 (0)	510 (12.7)	361 (20.1)
MVD, n (%)	NA	1,703 (55.7)	1,079 (32)	1,487 (52.2)	2,801 (60.7)	2,487 (61.9)	1,800 (100)
Previous MI, n (%)	1,380 (18.5)	113 (3.7)	299 (8.8)	52 (1.8)	1,167 (25.3)	631 (15.7)	387 (21.5)
Previous PCI, n (%)	1,726 (18.5)	262 (8.6)	345 (10.1)	184 (6.5)	1,583 (34.3)	916 (22.8)	74 (4.1)
Previous CABG, n (%)	275 (3.7)	18 (0.6)	6 (0.2)	6 (0.2)	400 (8.7)	245 (6.1)	NA
Previous stroke, n (%)	175 (2.4)	126 (4.1)	301 (8.9)	92 (3.2)	NA	NA	19 (1.1)
Previous bleeding, n (%)	48 (0.6)	NA	NA	NA	38 (0.8)	NA	NA

CABG = coronary artery bypass graft; CKD = chronic kidney disease; MVD = multivessel disease; NA = not available; NSTEMI = non–ST-segment elevation myocardial infarction; STEMI = ST-segment elevation myocardial infarction; other abbreviations as in Table 1.

a Chronic kidney disease is defined according to individual trial criteria.

b East Asian trials include TICO, T-PASS, and ULTIMATE-DAPT; TUXEDO-2 enrolled a South Asian population.

### Structure of the network meta-analysis

Figure 1 and Supplemental Figure 2 illustrate the network geometry of the 3 antithrombotic strategies used in each endpoint analysis: short DAPT with aspirin plus ticagrelor (1-3 months) followed by ticagrelor monotherapy, 12 months of aspirin plus ticagrelor (A + T), and 12 months of aspirin plus prasugrel (A + P). We set 12 months of aspirin plus prasugrel as the reference comparator. MACCE definitions varied across studies. However, all studies included death and MI as core components of the composite endpoint. Four studies defined MACCE as the composite of all-cause death, MI, or stroke.^15^^,^^16^^,^^26^^,^^27^ One study additionally included target vessel revascularization,^10^ whereas 2 studies defined MACCE as a composite of cardiac death, MI, stroke, ST, and target vessel revascularization.^8^^,^^9^ Major bleeding was defined according to Bleeding Academic Research Consortium (BARC) criteria as reported in each trial. Five studies used BARC type 3 or 5 definitions,^8^, ^9^, ^10^^,^^26^^,^^27^ whereas 2 studies defined major bleeding as BARC type 3, 4, or 5 events.^15^^,^^16^ Endpoint definitions for efficacy and safety analyses are provided in Supplemental Table 2.

**Figure 1:**
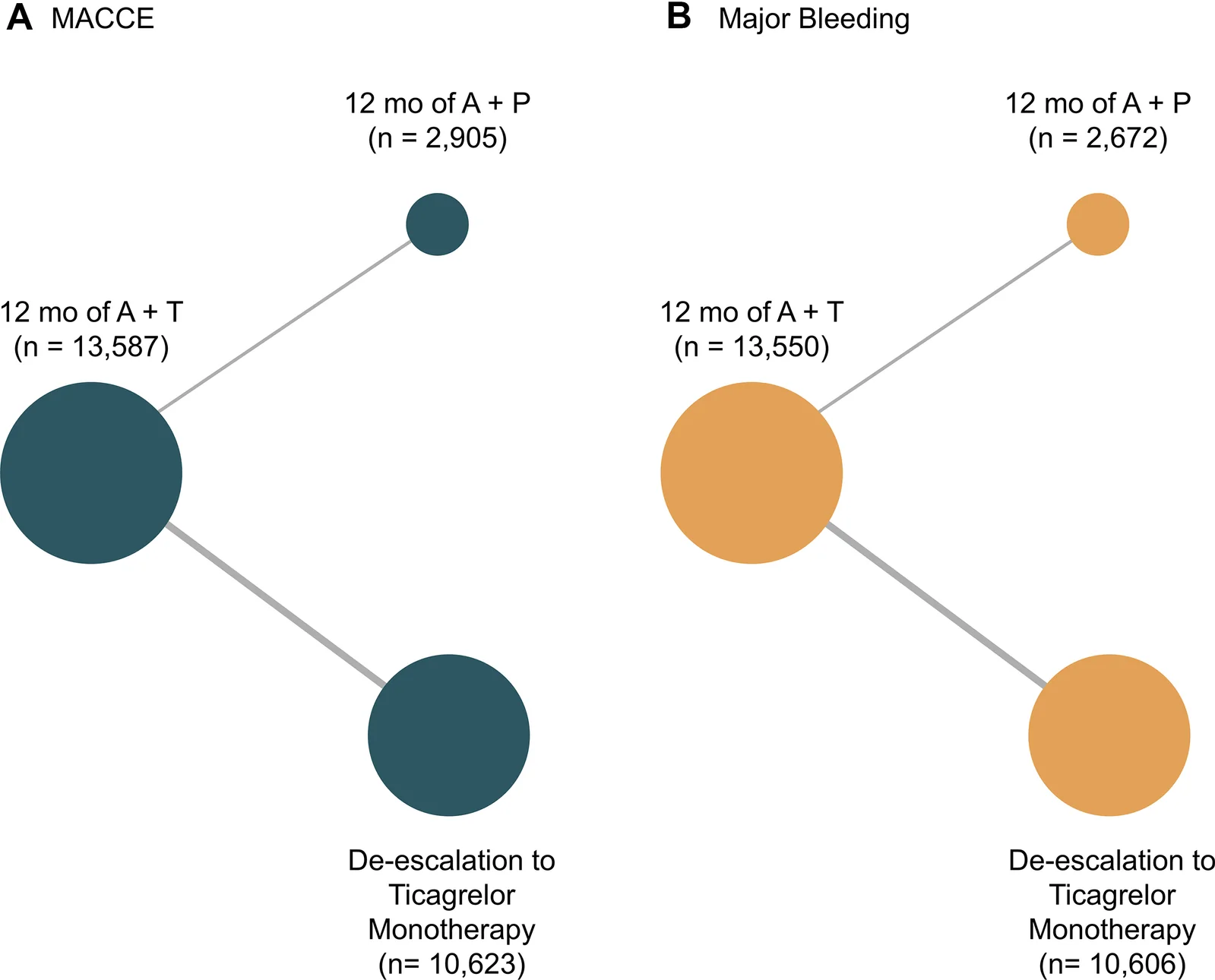
Primary Endpoints Network Plots (A) MACCE. (B) Major bleeding. Nodes represent antithrombotic strategies and edges represent direct comparisons in included trials. Node size correlates with patients assigned, and edge thickness correlates with the number of direct comparisons. Numbers may differ slightly between endpoints due to variations in outcome reporting across trials. A = aspirin; MACCE = major adverse cardiovascular and cerebrovascular events; P = prasugrel; T = ticagrelor.

### Primary endpoints

MACCE occurred in 1,100 of 27,115 patients (4.0% of patients). As shown in Figures 2A and 2B, 12 months of aspirin plus ticagrelor was associated with a higher risk of MACCE compared with aspirin plus prasugrel (IRR: 1.28; 95% CI: 1.07-1.53). De-escalation to ticagrelor monotherapy showed a numerically lower incidence of MACCE compared with ticagrelor-based DAPT (IRR: 0.85; 95% CI: 0.73-1.00), without reaching statistical significance. In the network meta-analysis, ticagrelor monotherapy showed similar MACCE rates compared with aspirin plus prasugrel (IRR: 1.09; 95% CI: 0.86-1.38); however, the CI did not exclude a clinically meaningful excess risk, and the prespecified exploratory noninferiority criterion was not met (P for noninferiority = 0.33). Major bleeding occurred in 661 of 26,828 patients (2.5%). As shown in Figures 2C and 2D, aspirin plus ticagrelor was not associated with a significant difference in major bleeding compared with aspirin plus prasugrel (IRR: 1.14; 95% CI: 0.85-1.54). Conversely, ticagrelor monotherapy significantly reduced major bleeding compared with both ticagrelor-based (IRR: 0.47; 95% CI: 0.36-0.61) and prasugrel-based DAPT (IRR: 0.54; 95% CI: 0.37-0.80). Pairwise analyses are shown in Supplemental Figures 3 and 4, and study-level data are reported in Supplemental Table 3.

**Figure 2:**
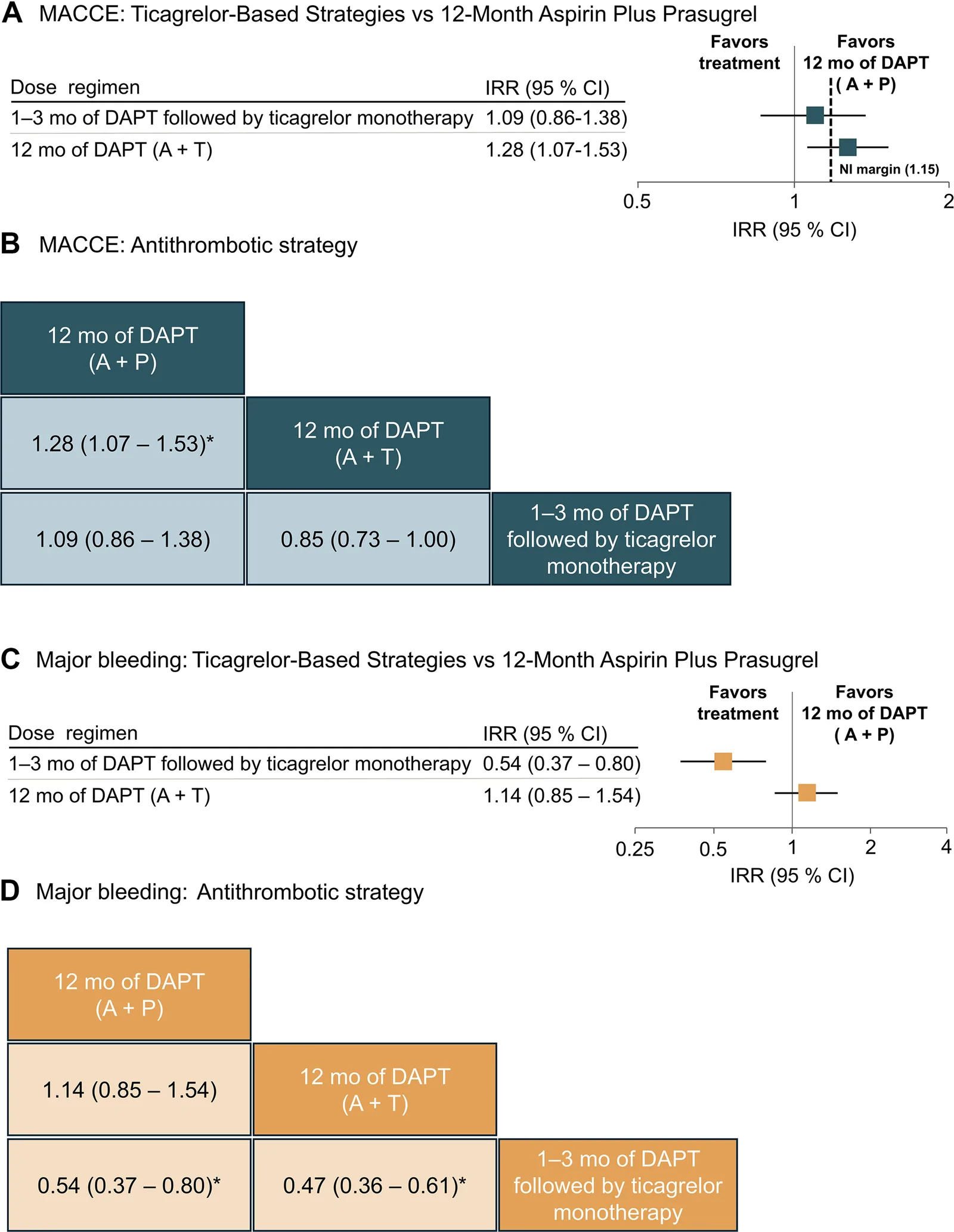
Network Estimates and League Tables for Primary Endpoints (A and C) Incidence rate ratios (IRRs) with 95% CIs from the random-effects network meta-analysis comparing ticagrelor-based strategies with 12 months of aspirin plus prasugrel (reference). The dashed vertical line indicates the prespecified noninferiority margin (IRR: 1.15). (B and D) League tables of treatment comparisons. Treatments are listed alphabetically. Data are presented as IRRs with 95% CIs. Comparisons should be read from right to left (row vs column treatment). IRRs <1 favor the row-defining treatment. ∗Statistically significant. DAPT = dual antiplatelet therapy; NI = noninferiority; other abbreviations as in Figure 1.

### Secondary endpoints

All-cause death occurred in 1.7% of patients and was lower with ticagrelor monotherapy compared with 12 months of aspirin plus ticagrelor (IRR: 0.76; 95% CI: 0.59-0.98). No significant differences were observed for cardiac death. In the network meta-analysis, aspirin plus ticagrelor was associated with a higher risk of MI compared with aspirin plus prasugrel (IRR: 1.38; 95% CI: 1.08-1.78). Stroke and ST were infrequent and did not differ across treatment strategies. Indirect estimates are summarized in Central Illustration A, with detailed forest plots provided in Supplemental Figures 5 to 9.

**Central Illustration:**
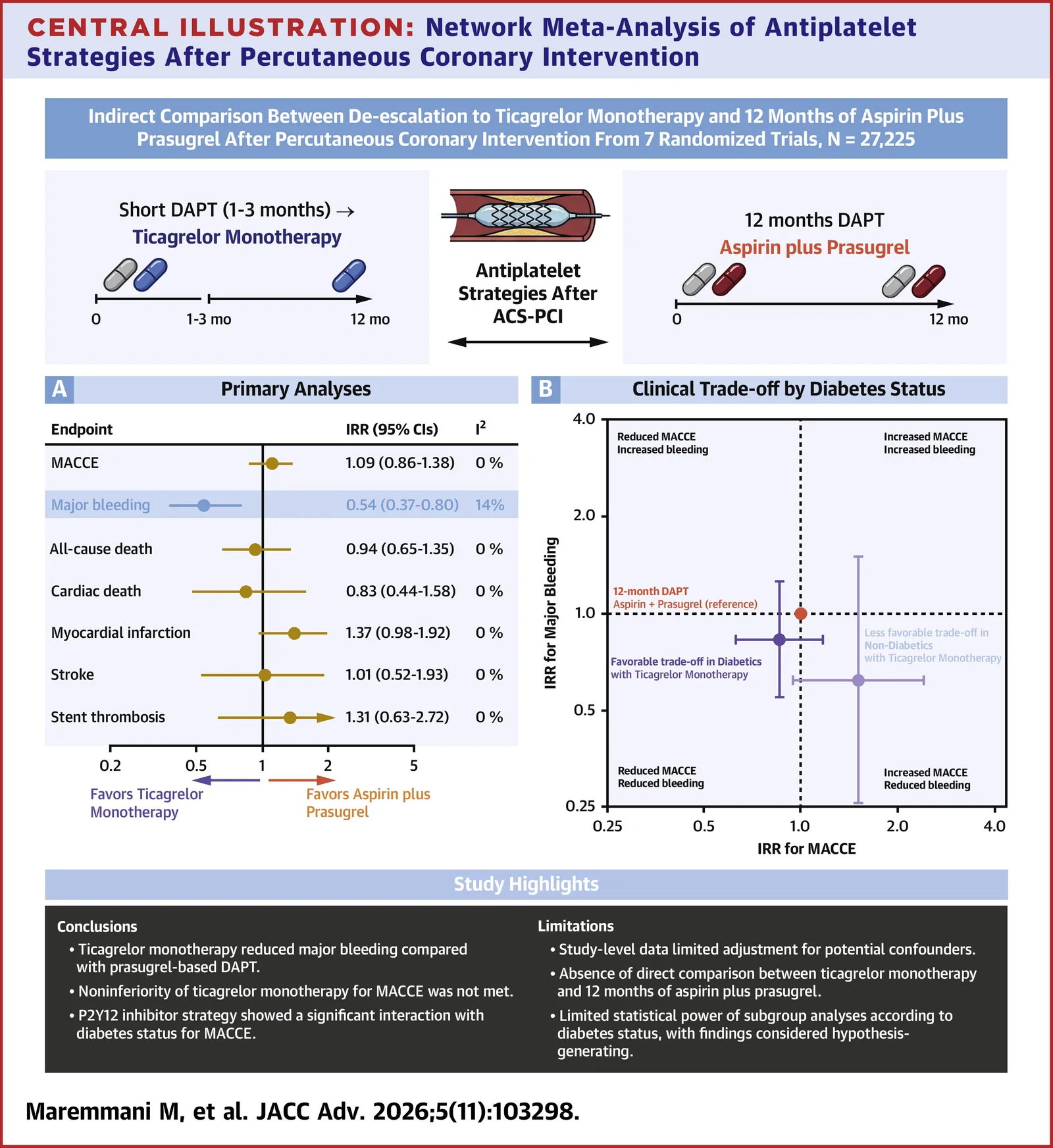
Network Meta-Analysis of Antiplatelet Strategies After Percutaneous Coronary Intervention This illustration depicts the indirect comparison of 2 antiplatelet strategies after PCI in patients with acute coronary syndrome and illustrates the ischemic-bleeding trade-off in patients with and without diabetes. ACS = acute coronary syndrome; DAPT = dual antiplatelet therapy; IRR = incidence rate ratio; MACCE = major adverse cardiovascular or cerebrovascular events; PCI = percutaneous coronary intervention.

### Ranking of treatment strategies

The P-score values for efficacy and safety endpoints are presented in Table 3 and Supplemental Figure 10. Twelve months of aspirin plus prasugrel ranked first for ischemic outcomes, including MACCE, MI, and ST. In contrast, ticagrelor monotherapy ranked best for major bleeding, with a concordant ranking for all-cause death (Supplemental Figure 11), and showed a favorable ischemic-bleeding trade-off (Figure 3). Stroke rankings were similar across antithrombotic strategies. Notably, 12 months of aspirin plus ticagrelor consistently had the lowest ranking across most endpoints.

**Table 3:** P-Scores for Each Antithrombotic Strategy and Endpoint

Antithrombotic Strategy	MACCE	Major Bleeding	All-Cause Death	Cardiac Death	Myocardial Infarction	Stroke	Stent Thrombosis
12 mo DAPT (aspirin plus prasugrel)	**0.8744**	0.4049	0.6539	0.5221	**0.9800**	0.5312	**0.8407**
1-3 mo of DAPT followed by ticagrelor monotherapy	0.6118	**0.9995**	**0.8094**	**0.7733**	0.2879	**0.5315**	0.4302
12 mo DAPT (aspirin plus ticagrelor)	0.0139	0.0956	0.0367	0.2046	0.2321	0.4372	0.2291

The regimen with the highest P-score for each endpoint is shown in **bold**.

Abbreviations as in Table 1.

**Figure 3:**
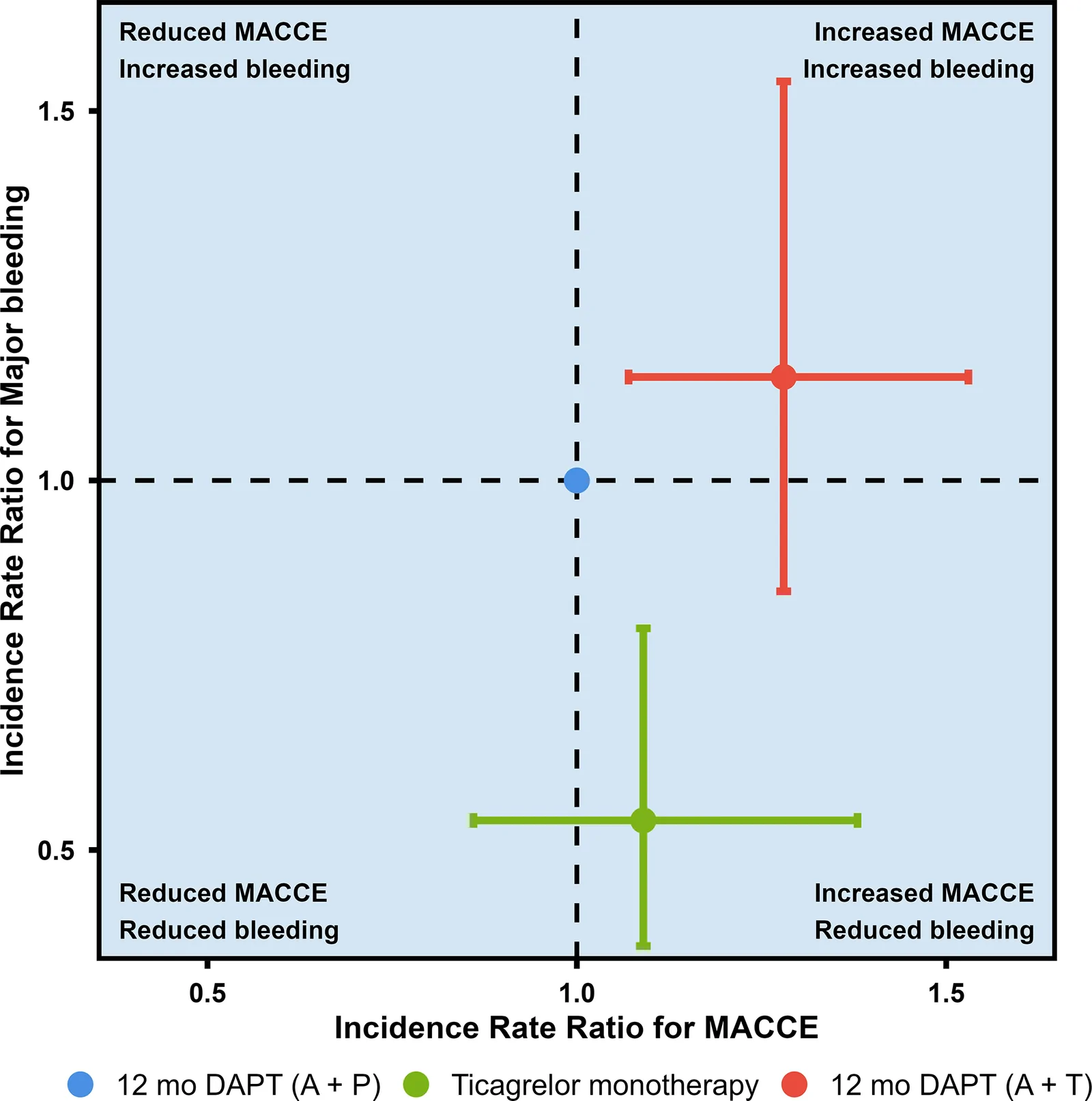
Benefit–Risk Tradeoff Between Major Adverse Cardiovascular and Cerebrovascular Events and Major Bleeding Incidence rate ratio for MACCE (x-axis) and major bleeding (y-axis) across antiplatelet strategies relative to 12 months of aspirin plus prasugrel (reference), with corresponding 95% CIs. Dashed lines indicate the line of no effect (incidence rate ratio: 1). Abbreviations as in Figures 1 and 2.

### Network consistency and heterogeneity

Because the network contained no closed treatment loops, global and local inconsistency could not be formally assessed (Supplemental Results 1). Heterogeneity was low across all endpoints (MACCE: I^2^ = 0%, *P* = 0.59; major bleeding: I^2^ = 14.7%, *P* = 0.32; all-cause death: I^2^ = 0%, *P* = 0.96; cardiac death: I^2^ = 0%, *P* = 0.77; MI: I^2^ = 0%, *P* = 0.46; stroke: I^2^ = 0%, *P* = 0.59; ST: I^2^ = 0%, *P* = 0.80). The contribution matrix showed that the indirect comparison between ticagrelor monotherapy and aspirin plus prasugrel was informed through the ticagrelor-based DAPT node, with 50% of the network estimate contributed by each direct comparison forming this pathway; ISAR-REACT 5 and TUXEDO-2 accounted for 32.7% and 17.3% of the overall indirect estimate, respectively (Supplemental Table 4).

### Sensitivity and meta-regression analyses

Sensitivity analyses were consistent with the primary findings. In ACS-restricted analyses, aspirin plus prasugrel remained the top-ranked strategy for MACCE, whereas ticagrelor monotherapy consistently ranked highest for major bleeding. Results were unchanged across analyses excluding East Asian populations and across sensitivity analyses according to the timing of aspirin withdrawal, including restriction to trials with ≥3 months of DAPT before monotherapy and exclusion of trials with very early aspirin discontinuation (<1 month after PCI; T-PASS). Analyses using common-effect models or pooled HRs yielded similar results. In leave-one-out analyses, exclusion of ISAR-REACT 5 altered the relative MACCE ranking of prasugrel- and ticagrelor-based strategies, although the extent to which this reflected single-study influence vs restriction to a diabetic population remains uncertain. Meta-regression showed no significant effect modification across study-level characteristics (Supplemental Results 2, Supplemental Figures 12 to 23, Supplemental Table 5).

### Subgroup analysis according to diabetes status

Prespecified analyses according to diabetes status demonstrated differential patterns of treatment effect across antiplatelet strategies. For MACCE, no significant interaction by diabetes status was observed for ticagrelor monotherapy vs 12-month aspirin plus ticagrelor (*P*int = 0.552), indicating a consistent effect of aspirin withdrawal irrespective of diabetes status (Supplemental Figure 24A). In contrast, significant interactions according to diabetes status were observed for comparisons involving ticagrelor- and prasugrel-based strategies. The relative effect of 12-month aspirin plus ticagrelor vs 12-month aspirin plus prasugrel differed according to diabetes status (*P*int = 0.019) (Supplemental Figure 24B), and a similar interaction was observed for the network comparison between ticagrelor monotherapy and prasugrel-based DAPT (*P*int = 0.04) (Figure 4, Supplemental Figures 25 and 26). In ranking analyses, ticagrelor monotherapy ranked highest for MACCE among patients with diabetes, whereas 12 months of aspirin plus prasugrel ranked highest among those without diabetes (Supplemental Table 6). For major bleeding, no significant interaction by diabetes status was observed, and ticagrelor monotherapy consistently ranked as the most favorable strategy in both subgroups (Figure 4, Supplemental Figures 27 to 29). Results were consistent when analyses were restricted to ACS populations (Supplemental Figures 30 and 31). Among patients with diabetes, ticagrelor monotherapy may provide a favorable ischemic-bleeding trade-off; however, this finding should be considered hypothesis-generating and should not be interpreted as evidence of superiority over prasugrel-based DAPT (Central Illustration B, Supplemental Figure 32). Study-level data for subgroup analyses are reported in Supplemental Table 7.

**Figure 4:**
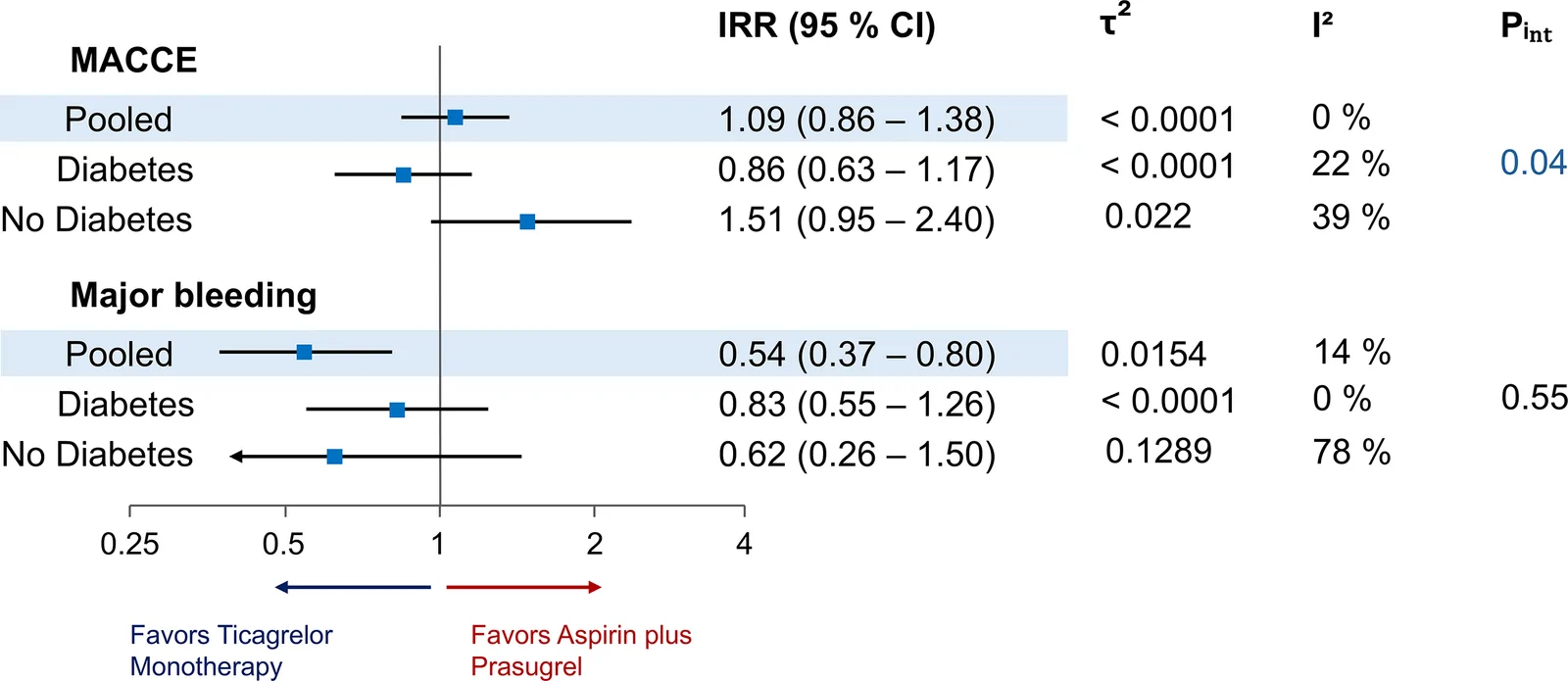
Network Meta-Analysis Estimates for Major Adverse Cardiovascular and Cerebrovascular Events and Major Bleeding by Diabetes IRR = incidence rate ratio; other abbreviations as in Figure 1.

### Quality assessment

Visual inspection of funnel plots did not suggest publication bias, whereas formal testing was not performed because of the limited number of included studies. Most studies were judged at low risk of bias, with 2 showing some concerns related to subgroup analyses of randomized trials. GRADE assessment indicated high certainty for most direct comparisons and moderate certainty for the indirect comparison. Detailed results are reported in the Supplemental Results 3, Supplemental Figures 33 and 34, and Supplemental Tables 8 to 10.

## Discussion

The principal findings of this network meta-analysis are as follows. First, 12 months of aspirin plus ticagrelor was associated with a 28% higher risk of MACCE compared with 12 months of aspirin plus prasugrel, supporting prasugrel-based DAPT as an important reference strategy for ischemic risk reduction in this setting and highlighting the need to evaluate ticagrelor monotherapy against this strategy. Second, although ticagrelor monotherapy after short DAPT was associated with similar rates of MACCE to 12 months of aspirin plus prasugrel, residual uncertainty remained and a clinically meaningful excess ischemic risk could not be excluded. Third, ticagrelor monotherapy significantly reduced major bleeding compared with 12-month DAPT strategies. Fourth, the reduction in bleeding was directionally associated with lower all-cause mortality. Fifth, diabetes appeared to modify treatment effects when comparing ticagrelor- and prasugrel-based strategies, whereas no clear interaction was observed according to aspirin withdrawal.

To our knowledge, this is the first network meta-analysis comparing de-escalation to ticagrelor monotherapy with 12-month prasugrel-based DAPT after PCI, while also exploring effect modification by diabetes status. In the overall population, 12-month aspirin plus prasugrel showed the highest P-score for MACCE; however, this ranking largely reflected its significant advantage over aspirin plus ticagrelor, whereas no significant difference was observed between aspirin plus prasugrel and ticagrelor monotherapy. This favorable comparison of prasugrel- vs ticagrelor-based DAPT is biologically plausible and consistent with prior randomized evidence and recent network meta-analytic data demonstrating superior protection from ischemic events with prasugrel compared with ticagrelor in patients undergoing PCI, supporting prasugrel-based DAPT as a contemporary reference strategy for ischemic risk reduction.^15^^,^^28^ This advantage likely reflects the synergistic inhibition of complementary platelet activation pathways achieved by DAPT. The continued use of aspirin in combination with a potent P2Y_12_ inhibitor provides sustained suppression of thromboxane A_2_-mediated platelet activation, a key driver of coronary atherothrombosis and early ST after PCI. In our analysis, the network estimate for MACCE comparing ticagrelor monotherapy with prasugrel-based DAPT was close to unity, with relatively wide CIs, precluding firm conclusions regarding a modest ischemic benefit or harm. Importantly, this comparison was entirely indirect through ticagrelor-based DAPT and depended on the ISAR-REACT 5 and TUXEDO-2 trials, with ISAR-REACT 5 providing the larger contribution to the prasugrel-based comparison. In this context, our exploratory noninferiority analysis underscores the persistence of clinical uncertainty and supports a conservative interpretation, avoiding unwarranted claims of equivalence. Overall, these findings reinforce 12 months of aspirin plus prasugrel as a contemporary reference strategy for ischemic risk reduction after PCI for ACS, while highlighting the need for direct comparisons before assuming comparable ischemic protection with ticagrelor monotherapy. Conversely, de-escalation to ticagrelor monotherapy significantly reduced major bleeding compared with both DAPT strategies, suggesting that prolonged exposure to aspirin combined with potent P2Y_12_ inhibition is a key driver of bleeding after PCI. Importantly, the reduction in bleeding was directionally associated with lower mortality, underscoring the prognostic impact of bleeding events.

These findings are consistent with recent meta-analyses showing that, after PCI, abbreviated DAPT followed by P2Y_12_ inhibitor monotherapy is associated with a significant reduction in bleeding compared with continued DAPT.^29^^,^^30^ However, although no significant increase in MACCE was observed, the width of the CIs precluded exclusion of a clinically meaningful excess ischemic risk, leaving residual uncertainty regarding whether aspirin-free strategies preserve the degree of ischemic protection achieved with prolonged DAPT. Moreover, these analyses pooled different P2Y_12_ inhibitors within both DAPT and monotherapy strategies, thereby evaluating treatment approaches according to aspirin exposure rather than according to the specific P2Y_12_ inhibitor used. Importantly, evidence from randomized trials has shown important differences in efficacy and safety across individual P2Y_12_ inhibitors, suggesting that treatment effects may not be fully explained by a class effect alone.^5^^,^^6^^,^^15^^,^^16^ Consequently, pooling ticagrelor-, prasugrel-, and clopidogrel-based regimens may dilute clinically relevant differences related to P2Y_12_ inhibitor selection and provide limited guidance regarding the optimal antiplatelet strategy for individual patients. In contrast, our analysis specifically contrasts ticagrelor monotherapy with prasugrel-based DAPT, thereby separating the effects of aspirin withdrawal from those related to the choice of potent P2Y_12_ inhibition. This distinction is particularly relevant given the current divergence between ACC/AHA and ESC guideline recommendations. The 2025 ACC/AHA guidelines endorse ticagrelor monotherapy after abbreviated DAPT as a routine strategy following PCI for ACS, whereas the ESC guidelines continue to support 12 months of DAPT, preferably with prasugrel, as the default antiplatelet strategy. In this context, our findings further support the role of prasugrel-based DAPT as an important contemporary reference strategy for ischemic risk reduction.

An important hypothesis-generating finding of the present study is the significant interaction between treatment effect and diabetes status. Diabetes is a well-recognized determinant of recurrent ischemic events after ACS and PCI, yet the relative efficacy of different P2Y_12_ inhibitors in this population remains uncertain. Although prasugrel is often perceived as providing greater ischemic protection than ticagrelor in patients with diabetes, direct comparative evidence is inconclusive. In the ISAR-REACT 5 trial, prasugrel was superior to ticagrelor in the overall ACS population, but this advantage was not observed among patients with diabetes.^31^ Conversely, the TUXEDO-2 trial, conducted exclusively in patients with diabetes and multivessel coronary artery disease undergoing PCI, did not demonstrate the noninferiority of ticagrelor compared with prasugrel for net clinical benefit. Rather than indicating a consistent divergence of treatment effect, these findings collectively underscore the lack of definitive evidence regarding the optimal P2Y_12_ inhibitor in patients with diabetes.^16^

Our meta-analysis demonstrated that diabetes status significantly modified the effect of ticagrelor- vs prasugrel-based strategies on MACCE. Notably, this interaction was observed both in the direct comparison between 12-month aspirin plus ticagrelor and 12-month aspirin plus prasugrel and in the network comparison between ticagrelor monotherapy and prasugrel-based DAPT. In contrast, no interaction was observed for the comparison between ticagrelor monotherapy and 12-month aspirin plus ticagrelor. Taken together, these findings suggest that the modifying effect of diabetes may be primarily related to differences between P2Y_12_ inhibitors rather than to aspirin discontinuation itself. Importantly, the higher MACCE ranking of ticagrelor monotherapy among patients with diabetes should not be interpreted as evidence of superiority over prasugrel-based DAPT. No interaction according to diabetes status was observed for major bleeding.

Although the biological basis of this interaction remains speculative, several mechanisms may plausibly explain the observed findings. Mechanistically, diabetes is characterized by enhanced platelet reactivity, upregulation of P2Y_12_ signaling pathways, and accelerated platelet turnover, resulting in a greater proportion of newly generated hyper-reactive platelets that may escape sustained pharmacologic inhibition.^32^^,^^33^ In addition, oxidative stress, chronic inflammation, endothelial dysfunction, and microvascular impairment contribute to a prothrombotic vascular milieu that further amplifies ischemic risk after ACS. In this context, differences in the pharmacologic profiles of P2Y_12_ inhibitors may become particularly relevant. Pharmacodynamic studies in patients with diabetes and coronary artery disease have shown that ticagrelor may achieve lower platelet reactivity compared with prasugrel, suggesting more consistent inhibition of adenosine diphosphate–mediated platelet activation in this high-risk population.^34^^,^^35^ Beyond platelet inhibition, ticagrelor exerts adenosine-mediated effects through inhibition of equilibrative nucleoside transporter-1, increasing extracellular adenosine levels. Elevated adenosine has been associated with enhanced coronary vasodilation, improved microvascular perfusion, and modulation of inflammatory and endothelial pathways.^36^, ^37^, ^38^ Consistently, in a randomized crossover study of diabetic patients with non–ST-segment elevation ACS undergoing PCI, ticagrelor was associated with greater reductions in inflammatory cytokines and improved endothelial function compared with prasugrel.^39^ In addition, prasugrel requires hepatic bioactivation via cytochrome P450 enzymes, whereas ticagrelor is directly active, potentially resulting in more predictable platelet inhibition in clinical settings characterized by metabolic alterations and polypharmacy. In contrast, among patients without diabetes, 12 months of aspirin plus prasugrel appeared to maintain the most favorable ranking for MACCE, albeit at the cost of increased major bleeding. This pattern suggests that, in this population, ischemic protection beyond the early post-PCI phase may be primarily driven by potent P2Y_12_ inhibition, whereas bleeding risk is largely related to continued aspirin exposure. Within this framework, removal of aspirin while maintaining prasugrel may represent a potential strategy to preserve ischemic protection while mitigating bleeding risk. Aspirin-free prasugrel strategies have recently been tested, but available evidence remains limited by predominantly East Asian populations and reduced-dose prasugrel regimens, thereby restricting generalizability to western practice.^40^^,^^41^ Our network meta-analysis suggests that the choice of P2Y_12_ inhibitor and the timing of aspirin withdrawal represent distinct components of antiplatelet therapy after PCI. Aspirin withdrawal after an initial period of DAPT was consistently associated with a lower bleeding risk across subgroups, whereas the relative effects of ticagrelor- and prasugrel-based strategies appeared to vary according to diabetes status. These findings support a more individualized approach to antiplatelet therapy after PCI, in which diabetes may inform P2Y_12_ inhibitor selection, while bleeding risk may guide early aspirin discontinuation. Future randomized trials should stratify patients according to diabetes status and directly compare potent P2Y_12_ inhibitor monotherapy strategies (ticagrelor or prasugrel) with 12 months of aspirin plus prasugrel, which currently represents an important reference strategy for ischemic protection.

### Study limitations

This study has several limitations. First, the absence of patient-level data precluded adjustment for individual patient characteristics, including differences in diabetes prevalence across treatment groups. Moreover, the substantial representation of East Asian populations may limit the generalizability of the findings to other populations, although sensitivity analyses excluding East Asian trials yielded findings consistent with the primary analysis. Second, the comparison between ticagrelor monotherapy and aspirin plus prasugrel relies on indirect evidence in the absence of head-to-head randomized trials, reflecting an important evidence gap in contemporary antiplatelet therapy. Third, despite the predominance of ACS populations (98.6%), 1 trial included a small proportion of non-ACS patients; however, analyses restricted to ACS populations were consistent. Fourth, definitions of MACCE varied across trials, although heterogeneity was low and analyses of individual components showed consistent directional effects. Fifth, subgroup analyses according to diabetes status were based on smaller sample sizes and should be considered hypothesis-generating. Sixth, although transitivity appeared acceptable, differences in PCI characteristics across trials, including stent platforms and intracoronary imaging use, may represent a source of clinical heterogeneity. Despite these limitations, the overall pattern of the network estimates and the biological plausibility of the findings support the clinical relevance of this analysis in addressing an important knowledge gap.

## Conclusions

In this network meta-analysis of predominantly ACS patients undergoing PCI, ticagrelor monotherapy reduced major bleeding compared with 12 months of aspirin plus prasugrel, whereas it did not meet noninferiority for MACCE. Direct randomized comparisons are warranted.

Perspectives **COMPETENCY IN PATIENT CARE:** In patients with ACS undergoing PCI, prasugrel-based DAPT remains an important contemporary reference strategy when ischemic risk reduction is prioritized. Ticagrelor monotherapy reduces major bleeding, but comparable ischemic protection has not been established.

**TRANSLATIONAL OUTLOOK:** Future randomized trials should directly compare ticagrelor monotherapy with aspirin plus prasugrel and evaluate whether diabetes modifies the relative efficacy and safety of these strategies.

## Funding support and author disclosures

The authors have reported that they have no relationships relevant to the contents of this paper to disclose.
