# Supplementary material for: Ticagrelor Monotherapy or Aspirin Plus Prasugrel After Percutaneous Coronary Intervention: A Network Meta-Analysis
Source: JACC Adv. 2026 Oct 5;5(11):103298. doi: 10.1016/j.jacadv.2026.103298 (PMC13671373; doi:10.1016/j.jacadv.2026.103298)
Supplement: Supplementary file 1 [file mmc1.pdf]

SUPPLEMENTARY MATERIAL

Ticagrelor Monotherapy or Aspirin Plus Prasugrel After Percutaneous Coronary Intervention: A Network Meta-Analysis

Michele Maremmani, MD,[a] Enrico Citerni, MD,[b] Ovidio De Filippo, MD,[c] Raffaele Piccolo, MD, PhD,[d] Alberto Menozzi, MD,[a] Carlos M. Campos, MD, PhD,[e] Francesco Costa, MD, PhD,[f] Fabrizio D'Ascenzo, MD, PhD[c], Christian Templin, MD, PhD[g,h], Davide Di Vece, MD[h,i]

[a]Department of Cardiology, Sant'Andrea Hospital, La Spezia, Italy; [b]Department of Cardiology, Campus Bio-Medico University Hospital, Rome, Italy; [c]Division of Cardiology, Department of Medical Sciences, AOU Città della Salute e della Scienza, University of Turin, Turin, Italy; [d]Department of Advanced Biomedical Sciences, University of Naples "Federico II", Naples, Italy; [e]Heart Institute (InCor), Universidade de São Paulo (USP), São Paulo, Brazil; [f]Department of Biomedical and Dental Sciences and Morphological and Functional Imaging, University of Messina, Messina, Italy; A.O.U. Policlinic "G. Martino", Messina, Italy; [g]Center for Molecular Cardiology, Schlieren Campus, University of Zurich, Zurich, Switzerland; [h]Department of Internal Medicine B, University of Medicine Greifswald, Greifswald, Germany; [i]Department of Internal Medicine, University of Genoa, Genoa, Italy.

Address for correspondence:
Corresponding author:
Davide Di Vece, MD
Department of Internal Medicine B, University of Medicine Greifswald, Greifswald, Germany.
E-mail: Davide.DiVece@med.uni-greifswald.de

Co-corresponding author:
Michele Maremmani, MD
Department of Cardiology, Ospedale Civile Sant'Andrea, Via Vittorio Veneto 197, 19124, La Spezia, Italy.
E-mail: michelemaremmani.90@gmail.com

Table of Contents

Supplementary Methods 1. PRISMA-NMA Checklist.. 4

Supplementary Methods 2. PRISMA Abstract Checklist.. 7

Supplementary Methods 3. Details of the Search Strategy According to the Database 8

Supplementary Figure 1. PRISMA Flow Diagram of Study Screening and Selection 9

Supplementary Table 1. Baseline characteristics across treatment nodes. 10

Supplementary Figure 2. Network geometry and study connections for each endpoint. 11

Supplementary Table 2. Endpoint definition for each included studies 12

Supplementary Figure 3. Pairwise and Network Meta-analyses for MACCE 13

Supplementary Figure 4. Pairwise and Network Meta-analyses for major bleeding.. 14

Supplementary Table 3. Study-Level Event Counts and Effect Estimates Used for the Network Meta-analysis 15

Supplementary Figure 5. Pairwise and Network Meta-analyses for all-cause death.. 17

Supplementary Figure 6. Pairwise and Network Meta-analyses for cardiac death 18

Supplementary Figure 7. Pairwise and Network Meta-analyses for myocardial infarction 19

Supplementary Figure 8. Pairwise and Network Meta-analyses for stroke.. 20

Supplementary Figure 9. Pairwise and Network Meta-analyses for stent thrombosis 21

Supplementary Figure 10. Ranking probabilities (rankograms) for primary and secondary endpoints 22

Supplementary Figure 11. Relationship Between Major Bleeding and All-Cause Death.. 25

Supplementary Results 1. Consistency and Heterogeneity Assessment.. 26

Supplementary Table 4. Contribution Matrix (direct and indirect) for the Network Meta-analysis (All End Points).. 27

Supplementary Results 2. Sensitivity Analysis 28

Supplementary Figure 12. League Tables and Rankograms for the Sensitivity Analysis of MACCE Restricted to the ACS Population.. 30

Supplementary Figure 13. League Tables and Rankograms for the Sensitivity Analysis of MACCE Restricted to the Non–East Asian Population.. 30

Supplementary Figure 14. League tables and rankograms for sensitivity analyses of MACCE according to timing of aspirin withdrawal.. 31

Supplementary Figure 15. League Tables and Rankograms for the Sensitivity Analysis of MACCE Using a Common-Effect Model.. 32

Supplementary Figure 16. League Tables and Rankograms for the Sensitivity Analysis of MACCE Using Pooled Hazard Ratios (Time-to-Event Analysis) 32

Supplementary Figure 17. League Tables and Rankograms for the Sensitivity Analysis of Major Bleeding Restricted to the ACS Population.. 33

Supplementary Figure 18. League Tables and Rankograms for the Sensitivity Analysis of Major Bleeding Restricted to the Non–East Asian Population.. 33

Supplementary Figure 19. League tables and rankograms for sensitivity analyses of major bleeding according to timing of aspirin withdrawal.. 34

Supplementary Figure 20. League Tables and Rankograms for the Sensitivity Analysis of Major Bleeding Using a Common-Effect Model 35

Supplementary Figure 21. League Tables and Rankograms for the Sensitivity Analysis of Major Bleeding Using Pooled Hazard Ratios (Time-to-Event Analysis)........ 35

Supplementary Figure 22. Forest plot and Rankogram for the Leave-One-Out Sensitivity Analysis of MACCE Excluding ISAR-REACT 5........ 36

Supplementary Figure 23. Forest plot and Rankogram for the Leave-One-Out Sensitivity Analysis of MACCE Excluding TUXEDO-2........ 36

Supplementary Table 5. Meta-regression Analyses........ 37

Supplementary Figure 24. Pairwise meta-analysis of the interaction between diabetes status and treatment effect on the MACCE endpoint........ 39

Supplementary Figure 25. Network meta-analysis forest plots, league tables, and ranking probabilities (rankograms) for MACCE in subgroup analyses of patients with diabetes........ 40

Supplementary Figure 26. Network meta-analysis forest plots, league tables, and ranking probabilities (rankograms) for MACCE in subgroup analyses of patients without diabetes........ 41

Supplementary Table 6. P-scores for Each Antithrombotic Strategy stratified by Diabetes status........ 42

Supplementary Figure 27 Pairwise meta-analysis of the interaction between diabetes status and treatment effect on major bleeding endpoint........ 43

Supplementary Figure 28. Network meta-analysis forest plots, league tables, and ranking probabilities (rankograms) for the major bleeding endpoint in patients with diabetes........ 44

Supplementary Figure 29. Network meta-analysis forest plots, league tables, and ranking probabilities (rankograms) for the major bleeding endpoint in patients without diabetes........ 45

Supplementary Figure 30. Sensitivity analysis for MACCE restricted to ACS patients with diabetes, excluding GLASSY, TWILIGHT, and TUXEDO-2, and including TWILIGHT-Diabetes-ACS........ 46

Supplementary Figure 31. Sensitivity analysis for major bleeding restricted to ACS patients with diabetes, excluding GLASSY, TWILIGHT, and TUXEDO-2, and including TWILIGHT-T2DM-ACS........ 47

Supplementary Figure 32. Benefit–Risk Tradeoff Between MACCE and Major Bleeding in Patients With Diabetes........ 48

Supplementary Table 7. Study-Level Event Counts and Effect Estimates Stratified by Diabetes Status 49

Supplementary Results 3. Quality Assessment and Risk of Bias........ 50

Supplementary Figure 33. Publication Bias Assessment........ 51

Supplementary Figure 34. RoB 2 - Cochrane tool for assessing the risk of bias in randomized clinical trials 55

Supplementary Table 8. GRADE certainty of evidence assessment for direct estimates........ 56

Supplementary Table 9. GRADE certainty of evidence assessment for indirect estimates........ 57

Supplementary Table 10. GRADE certainty of evidence assessment for network estimates........ 58

References........ 59

Supplementary Methods 1. PRISMA-NMA Checklist

Topic	No.	Item	Location where item is reported
**TITLE**			
**Title**	1	Identify the report as a systematic review incorporating a network meta-analysis.	Pag.1
**ABSTRACT**			
**Abstract**	2	See the PRISMA 2020 for Abstracts checklist	Supplementary Methods 2
**INTRODUCTION**			
**Rationale**	3	Describe the rationale for the review in the context of existing knowledge, including mention of why a network meta-analysis has been conducted.	Pag.4
**Objectives**	4	Provide an explicit statement of the objective(s) or question(s) the review addresses.	Pag.4
**METHODS**			
**Protocol and registration**	5	Indicate whether a review protocol exists and if and where it can be accessed; and if available, provide a registration number.	Pag. 5
**Eligibility criteria**	6	Specify study characteristics (e.g., PICOS, length of follow-up) and report characteristics (e.g., years considered, language, publication status) used as criteria for eligibility, giving rationale. Clearly describe eligible treatments included in the treatment network and note whether any have been clustered or merged into the same node (with justification).	Pag.5
**Information sources**	7	Describe all information sources (e.g., databases with dates of coverage, contact with study authors to identify additional studies) in the search and date last searched.	Pag.5
**Search strategy**	8	Present the full search strategies for all databases, registers and websites, including any filters and limits used.	Supplementary Methods 3
**Study selection**	9	State the process for selecting studies (i.e., screening, eligibility, included in systematic review, and, if applicable, included in the meta-analysis).	Pag.5-6<br>Supplementary Figure 1
**Data collection process**	10	Describe method of data extraction from reports (e.g., piloted forms, independently, in duplicate) and any processes for obtaining and confirming data from investigators.	Pag.5-6
**Data items**	11	Describe method of data extraction from reports (e.g., piloted forms, independently, in duplicate) and any processes for obtaining and confirming data from investigators.	Pag.6
**Geometry of the network**	S1	Describe methods used to explore the geometry of the treatment network under study and potential biases related to it. This should include how the evidence base has been graphically summarized for presentation, and what characteristics were compiled and used to describe the evidence base to readers.	Pag.8<br>Figure 1<br>Supplementary Figure 2
**Risk of bias within individual studies**	12	Describe methods used for assessing risk of bias of individual studies (including specification of whether this was done at the study or outcome level), and how this information is to be used in any data synthesis.	Pag. 6

Topic	No.	Item	Location where item is reported
**Summary measures**	13	State the principal summary measures (e.g., risk ratio, difference in means). Also describe the use of additional summary measures assessed, such as treatment rankings and surface under the cumulative ranking curve (SUCRA) values, as well as modified approaches used to present summary findings from meta-analyses.	Pag. 6-7
**Planned methods of analysis**	14a	Describe the processes used to decide which studies were eligible for each synthesis (e.g. tabulating the study intervention characteristics and comparing against the planned groups for each synthesis (item 5)).	Pag. 6-7<br>Supplementary Tables 1, 2 and 3
	14b	Describe any methods required to prepare the data for presentation or synthesis, such as handling of missing summary statistics, or data conversions.	Pag. 6-7
	14c	Describe any methods used to tabulate or visually display results of individual studies and syntheses.	Pag. 6-7<br>Supplementary Tables 1, 2 and 3
	14d	Describe any methods used to synthesize results and provide a rationale for the choice(s). If meta-analysis was performed, describe the model(s), method(s) to identify the presence and extent of statistical heterogeneity, and software package(s) used.	Pag. 6-7
	14e	Describe any methods used to explore possible causes of heterogeneity among study results (e.g. subgroup analysis, meta-regression).	Pag. 6-7
	14f	Describe any sensitivity analyses conducted to assess robustness of the synthesized results.	Pag. 6-7
**Assessment of Inconsistency**	S2	Describe the statistical methods used to evaluate the agreement of direct and indirect evidence in the treatment network(s) studied. Describe efforts taken to address its presence when found.	Pag. 6-7
**Risk of bias across studies**	15	Specify any assessment of the risk of bias that may affect the cumulative evidence (e.g., publication bias, selective reporting within studies).	Pag. 6-7
**Additional analyses**	16	Describe methods of additional analyses if done, indicating which were pre-specified. This may include, but not be limited to, the following:<br>• Sensitivity or subgroup analyses;<br>• Meta-regression analyses;<br>• Alternative formulations of the treatment network;<br>• Use of alternative prior distributions for Bayesian analyses (if applicable).	Pag. 6-7
**RESULTS**			
**Study selection**	17	Give numbers of studies screened, assessed for eligibility, and included in the review, with reasons for exclusions at each stage, ideally with a flow diagram.	Pag.7-8<br>Supplementary Figure 1
**Presentation of network structure**	S3	Provide a network graph of the included studies to enable visualization of the geometry of the treatment network.	Pag.8<br>Figure 1<br>Supplementary Figure 2
**Summary of network geometry**	S4	Provide a brief overview of characteristics of the treatment network. This may include commentary on the abundance of trials and randomized patients for the different interventions and pairwise comparisons in the network, gaps of evidence in the treatment network, and potential biases reflected by the network structure.	Pag.8<br>Figure 1<br>Supplementary Figure 2
**Study characteristics**	18	For each study, present characteristics for which data were extracted (e.g., study size, PICOS, follow-up period) and provide the citations.	Tables 1 and 2
**Risk of bias within studies**	19	Present data on risk of bias of each study and, if available, any outcome level assessment.	Supplementary Results 3<br>Supplementary Figures 31 and 32
**Results of individual studies**	20	For all outcomes, present, for each study:(a) summary statistics for each group (where appropriate) and (b) an effect estimates and its precision (e.g. confidence/credible interval), ideally using structured tables or plots.	Supplementary Figures 5-9

Topic	No.	Item	Location where item is reported
**Synthesis of results**	21	Present results of each meta-analysis done, including confidence/credible intervals. In larger networks, authors may focus on comparisons versus a particular comparator (e.g. placebo or standard care), with full findings presented in an appendix. League tables and forest plots may be considered to summarize pairwise comparisons. If additional summary measures were explored (such as treatment rankings), these should also be presented.	Pag. 9-10<br>Figure 2<br>Supplementary Figures 5-9
**Exploration for inconsistency**	S5	Describe results from investigations of inconsistency. This may include such information as measures of model fit to compare consistency and inconsistency models, P values from statistical tests, or summary of inconsistency estimates from different parts of the treatment network.	Supplementary Figures 5-9
**Risk of bias across studies**	22	Present assessments of risk of bias due to missing results (arising from reporting biases) for each synthesis assessed.	Supplementary Results 3<br>Supplementary Figures 31 and 32
**Results of additional analyses**	23	Give results of additional analyses, if done (e.g., sensitivity or subgroup analyses, meta-regression analyses, alternative network geometries studied, alternative choice of prior distributions for Bayesian analyses, and so forth).	Pag. 10-11<br>Figure 4<br>Supplementary Results 2<br>Supplementary Table 5<br>Supplementary Figures 12-21
**DISCUSSION**			
**Discussion**	24a	Provide a general interpretation of the results in the context of other evidence.	Pag. 12-15
	24b	Discuss any limitations of the evidence included in the review.	Pag. 15-16
	24c	Discuss any limitations of the review processes used.	Pag. 15-16
	24d	Discuss implications of the results for practice, policy, and future research.	Pag.14-15
**OTHER INFORMATION**			
**Support**	25	Describe sources of financial or non-financial support for the review, and the role of the funders or sponsors in the review.	Pag.1
**Competing interests**	26	Declare any competing interests of review authors.	Pag.1
**Availability of data, code and other materials**	27	Report which of the following are publicly available and where they can be found: template data collection forms; data extracted from included studies; data used for all analyses; analytic code; any other materials used in the review.	Supplementary Tables 1, 2 and 3

Supplementary Methods 2. PRISMA Abstract Checklist

Topic	No.	Item	Reported?
**TITLE**			
**Title**	1	Identify the report as a systematic review.	Yes
**BACKGROUND**			
**Objectives**	2	Provide an explicit statement of the main objective(s) or question(s) the review addresses.	Yes
**METHODS**			
**Eligibility criteria**	3	Specify the inclusion and exclusion criteria for the review.	Yes
**Information sources**	4	Specify the information sources (e.g. databases, registers) used to identify studies and the date when each was last searched.	Yes
**Risk of bias**	5	Specify the methods used to assess risk of bias in the included studies.	No
**Synthesis of results**	6	Specify the methods used to present and synthesize results.	Yes
**RESULTS**			
**Included studies**	7	Give the total number of included studies and participants and summarise relevant characteristics of studies.	Yes
**Synthesis of results**	8	Present results for main outcomes, preferably indicating the number of included studies and participants for each. If meta-analysis was done, report the summary estimate and confidence/credible interval. If comparing groups, indicate the direction of the effect (i.e. which group is favoured).	Yes
**DISCUSSION**			
**Limitations of evidence**	9	Provide a brief summary of the limitations of the evidence included in the review (e.g. study risk of bias, inconsistency and imprecision).	Yes
**Interpretation**	10	Provide a general interpretation of the results and important implications.	No
**OTHER**			
**Funding**	11	Specify the primary source of funding for the review.	No
**Registration**	12	Provide the register name and registration number.	Yes

Supplementary Methods 3. Details of the Search Strategy According to the Database

Database	Search strategy
**PubMed**	(("Percutaneous Coronary Intervention"[Mesh] OR "Coronary Angioplasty"[Mesh] OR PCI[Title/Abstract] OR "percutaneous coronary intervention"[Title/Abstract] OR "drug-eluting stent*"[Title/Abstract]) AND ("Ticagrelor"[Mesh] OR ticagrelor[Title/Abstract] OR "Prasugrel Hydrochloride"[Mesh] OR prasugrel[Title/Abstract]) AND (monotherap*[Title/Abstract] OR "dual antiplatelet therapy"[Title/Abstract] OR DAPT[Title/Abstract] OR de-escalat*[Title/Abstract] OR descalat*[Title/Abstract] OR deescalat*[Title/Abstract] OR short*[Title/Abstract] OR duration[Title/Abstract] OR discontinu*[Title/Abstract] OR withdraw*[Title/Abstract] OR "aspirin discontinuation"[Title/Abstract] OR "aspirin withdrawal"[Title/Abstract])) AND (randomized controlled trial[Publication Type] OR clinical trial[Publication Type] OR random*[Title/Abstract]) AND ("2000/01/01"[Date - Publication] : "3000"[Date - Publication])
**Embase**	('percutaneous coronary intervention'/exp OR 'coronary angioplasty'/exp OR pci:ti,ab OR 'percutaneous coronary intervention':ti,ab OR 'drug eluting stent*':ti,ab) AND ('ticagrelor'/exp OR ticagrelor:ti,ab OR 'prasugrel'/exp OR prasugrel:ti,ab) AND (monotherap*:ti,ab OR 'dual antiplatelet therapy':ti,ab OR dapt:ti,ab OR de-escalat*:ti,ab OR descalat*:ti,ab OR deescalat*:ti,ab OR short*:ti,ab OR duration:ti,ab OR discontinu*:ti,ab OR withdraw*:ti,ab OR 'aspirin discontinuation':ti,ab OR 'aspirin withdrawal':ti,ab) AND ('randomized controlled trial'/exp OR 'clinical trial'/exp OR random*:ti,ab) AND [2000-2026]/py
**Other Sources**	Abstracts and proceedings from major cardiology meetings (TCT, EuroPCR, ESC, AHA) and affiliated journals (JACC, EuroIntervention) were screened for relevant ongoing trials and abstract presentations of unpublished RCTs.

Supplementary Figure 1. PRISMA Flow Diagram of Study Screening and Selection

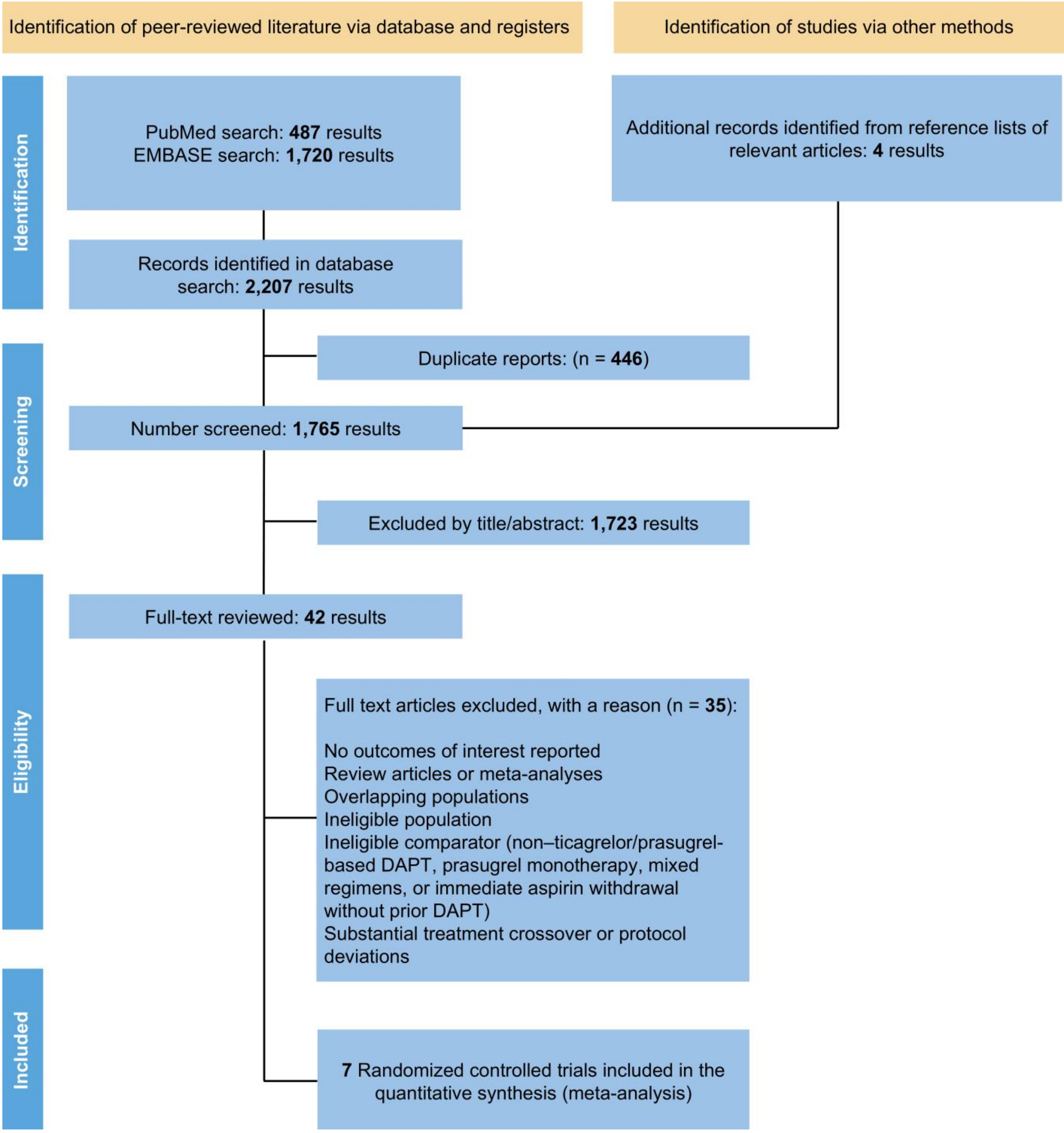

Supplementary Table 1. Baseline characteristics across treatment nodes.

Characteristic	De-escalation to Ticagrelor Monotherapy	12 mo DAPT (A + T)	12 mo DAPT (A + P)
**Mean age, years**	63	63	63
**T2DM, %**	28.2	31.8	45.6
**ACS, %**	100	99	93
**East Asian, %**	49	44	31

Supplementary Figure 2. Network geometry and study connections for each endpoint.

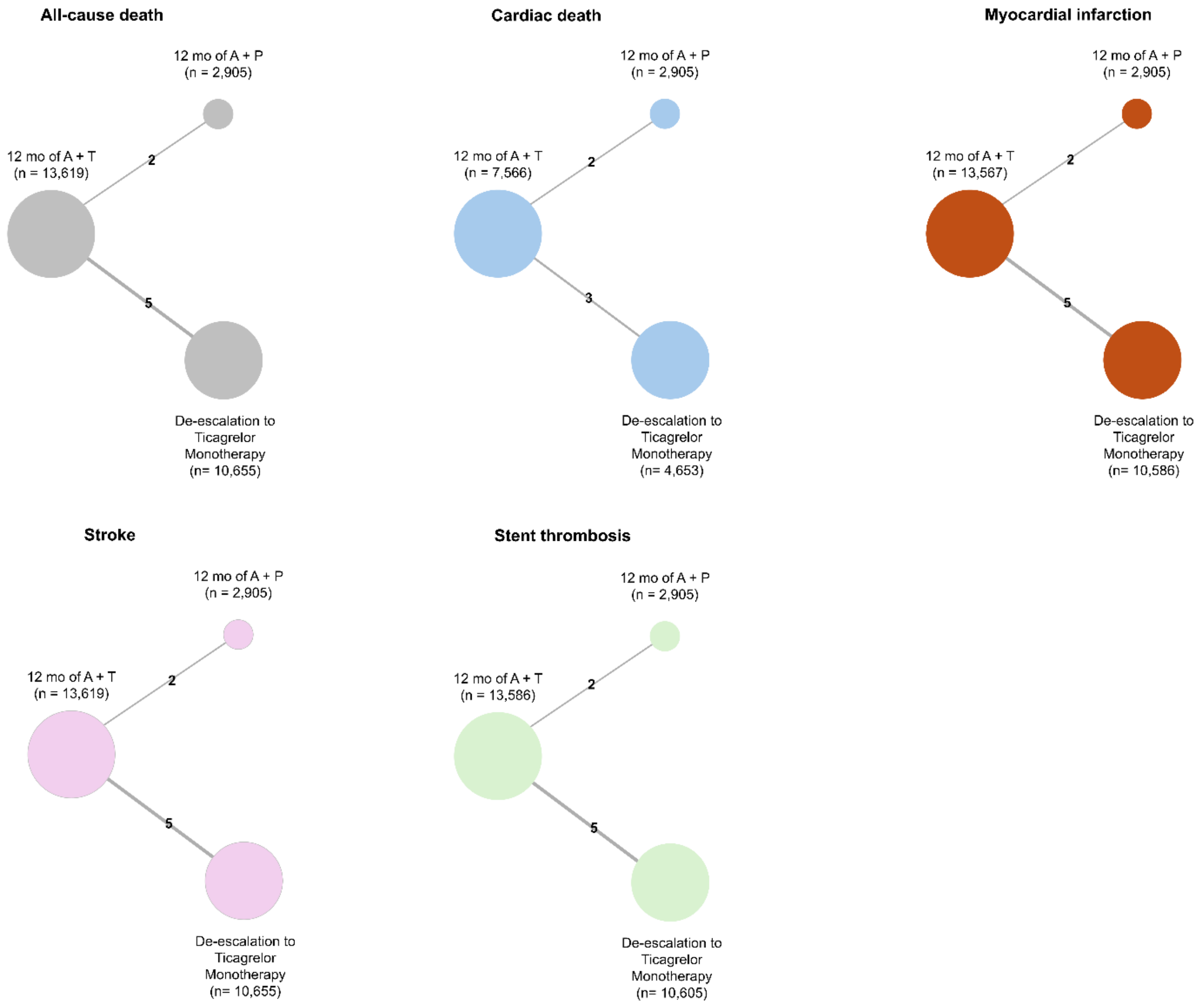

Nodes represent antithrombotic strategies and edges represent direct comparisons in included trials. Node size correlates with patients assigned, and edge thickness correlates with the number of direct comparisons.

Supplementary Table 2. Endpoint definition for each included studies

Study ID (First Author, Year)	MACCE	Major Bleeding	Stroke	Stent Thrombosis	Myocardial Infarction
**GLOBAL LEADERS-ACS (Tomaniak et al., 2019)[1]**	All-cause death, MI, or stroke	BARC 3 or 5	Ischemic stroke (TIA excluded)	Definite ST (ARC definition)	Site-reported MI according to the Third Universal Definition
**TICO (Kim et al., 2020)[2]**	All-cause death, MI, stroke, ST, or TVR	BARC 3 or 5	Neurological deficit >24 h or imaging-confirmed infarction/hemorrhage	Definite or probable ST (ARC definition)	Clinical symptoms or ECG/imaging changes with CK-MB elevation or troponin >99th percentile
**TWILIGHT-ACS (Baber et al., 2020)[3]**	All-cause death, MI, or stroke	BARC 3 or 5	Neurological dysfunction >24 h confirmed by imaging or CSF analysis (including TIA)	Definite, probable, or possible ST (ARC definition)	MI according to the Third Universal Definition
**T-PASS (Hong et al., 2023)[4]**	Cardiovascular death, MI, ST, or ischemia-driven TVR	BARC 3 or 5	Acute cerebrovascular event with neurological deficit or imaging-confirmed infarction	Definite or probable ST (ARC definition)	MI according to the Third Universal Definition
**ULTIMATE-DAPT (Ge et al., 2024)[5]**	Cardiac death, MI, stroke, ST, or clinically driven TVR	BARC 3 or 5	Neurological dysfunction >24 h with imaging or CSF evidence	Definite or probable ST (ARC definition)	MI according to the Third Universal Definition
**ISAR-REACT 5 (Schupke et al., 2019)[6]**	All-cause death, MI, or stroke	BARC 3–5	New neurological deficit due to ischemia or hemorrhage lasting >24 h or resulting in death	Definite or probable ST (ARC definition)	MI classified according to the Universal Definition (types 1, 2, 4a, 4b, 5)
**TUXEDO-2 (Bangalore et al., 2026)[7]**	All-cause death, MI, or stroke	BARC 3–5	Not specified	Definite or probable ST (ARC definition; acute, subacute, late)	MI according to the Third Universal Definition

Supplementary Figure 3. Pairwise and Network Meta-analyses for MACCE

Supplementary Figure 3A. Pairwise Meta-analysis of De-escalation to Ticagrelor Monotherapy vs 12 Months of Ticagrelor-Based DAPT

Study	De-escalation T- Monotherapy Events	PY	12 mo A + T Events	PY	IRR	95%-CI	Weight
GLOBAL LEADERS-ACS	67	3697	85	3680	0.78	[0.57; 1.08]	24.8%
TICO	35	1527	51	1529	0.69	[0.45; 1.06]	13.7%
TWILIGHT-ACS	96	2273	102	2341	0.97	[0.73; 1.28]	32.7%
T-PASS	21	1426	31	1424	0.68	[0.39; 1.18]	8.3%
ULTIMATE-DAPT	61	1700	63	1700	0.97	[0.68; 1.38]	20.5%
**Random effects model**					**0.85**	**[0.73; 1.00]**	**100.0%**

Heterogeneity: $I^2 = 0.0\%$, $\tau^2 = 0$, $p = 0.5233$

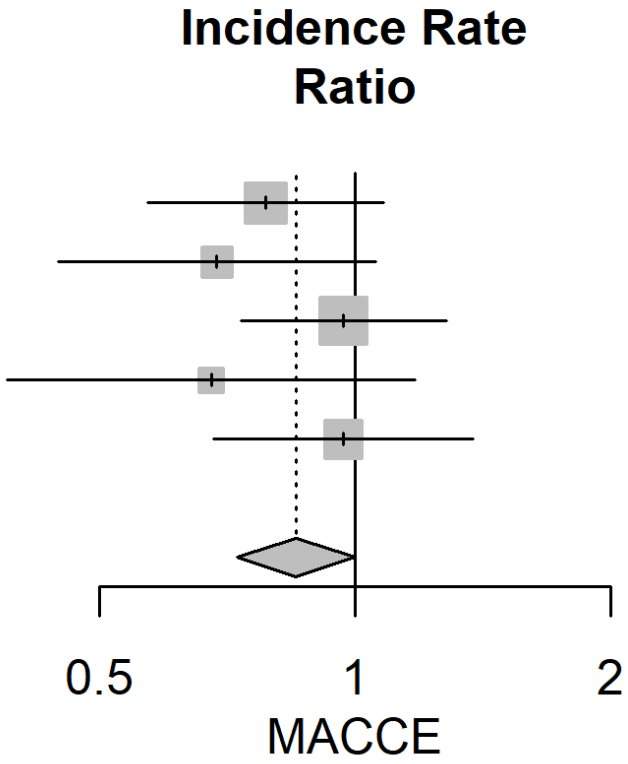

Supplementary Figure 3B . Pairwise Meta-analysis of 12 Months of Aspirin Plus Ticagrelor vs 12 Months of Aspirin Plus Prasugrel

Study	12 mo A + T Events	PY	12 mo A + P Events	PY	IRR	95%-CI	Weight
ISAR-REACT 5	184	2012	137	2006	1.34	[1.07; 1.67]	65.4%
TUXEDO-2	90	901	77	899	1.17	[0.86; 1.58]	34.6%
**Random effects model**					**1.28**	**[1.07; 1.53]**	**100.0%**

Heterogeneity: $I^2 = 0.0\%$, $\tau^2 = 0$, $p = 0.4715$

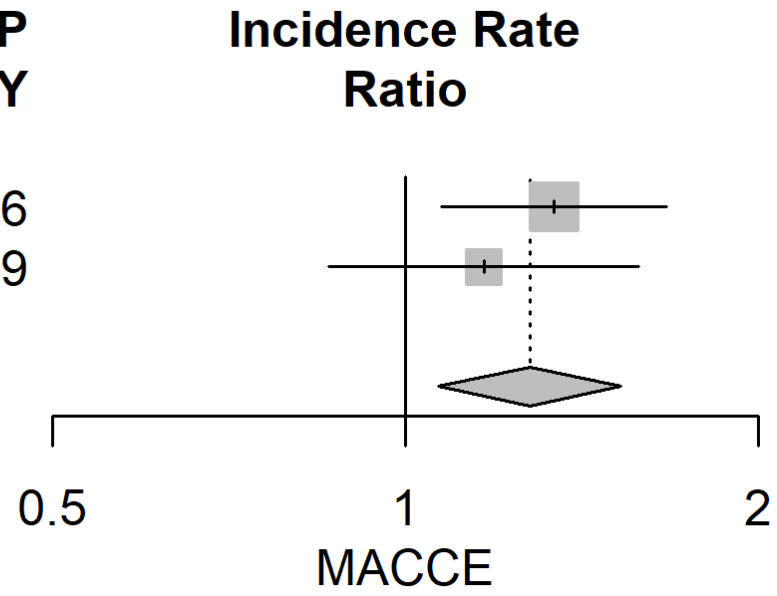

Supplementary Figure 3C . Random-Effects Network Meta-analysis Comparing Ticagrelor-Based Strategies With 12 Months of Aspirin Plus Prasugrel (Reference Treatment)

Treatment	IRR	95%-CI
12 mo A + T	1.28	[1.07; 1.53]
De-escalation T- Monotherapy	1.09	[0.86; 1.38]

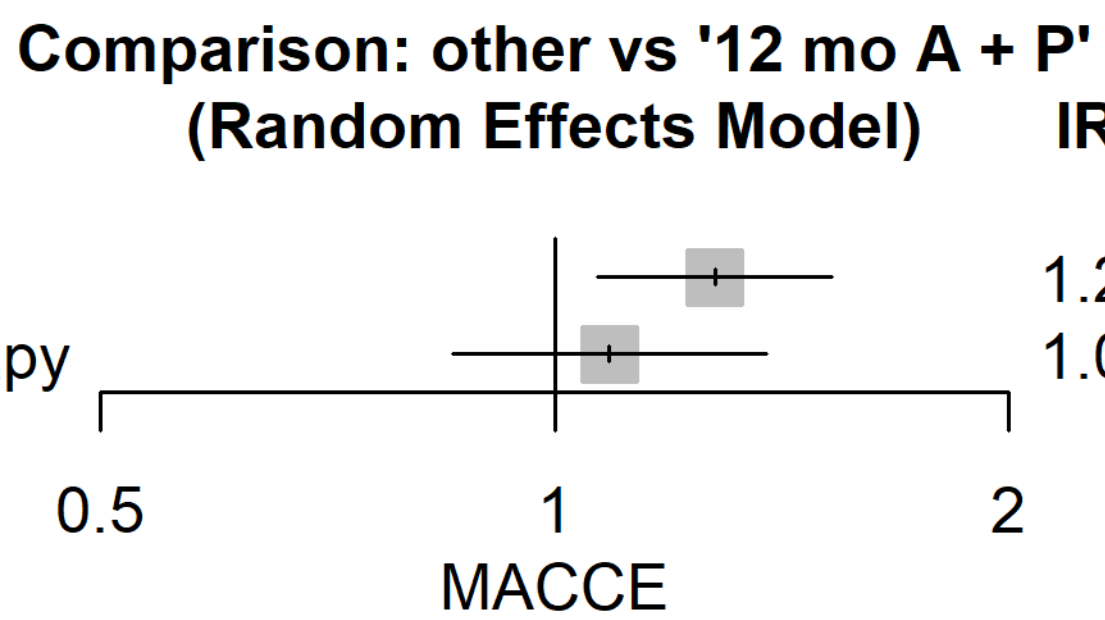

Heterogeneity: $I^2 = 0.0\%$, $\tau^2 < 0.0001$, $p = 0.5891$

Supplementary Figure 4. Pairwise and Network Meta-analyses for major bleeding

Supplementary Figure 4A. Pairwise Meta-analysis of De-escalation to Ticagrelor Monotherapy vs 12 Months of Ticagrelor-Based DAPT

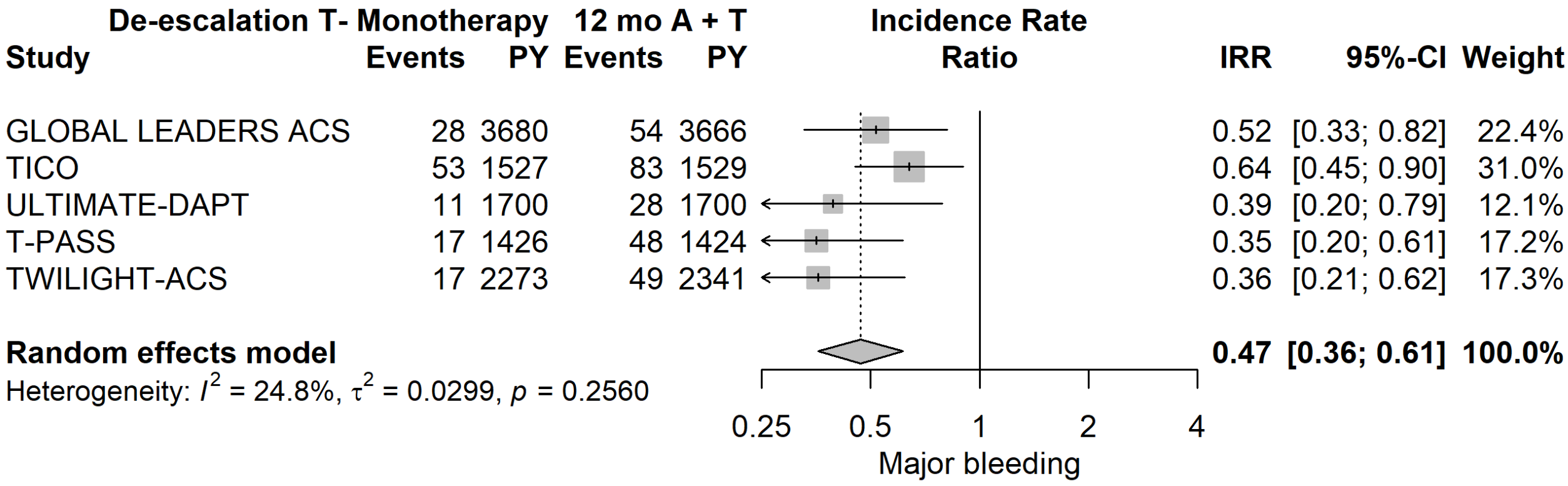

Supplementary Figure 4B . Pairwise Meta-analysis of 12 Months of Aspirin Plus Ticagrelor vs 12 Months of Aspirin Plus Prasugrel

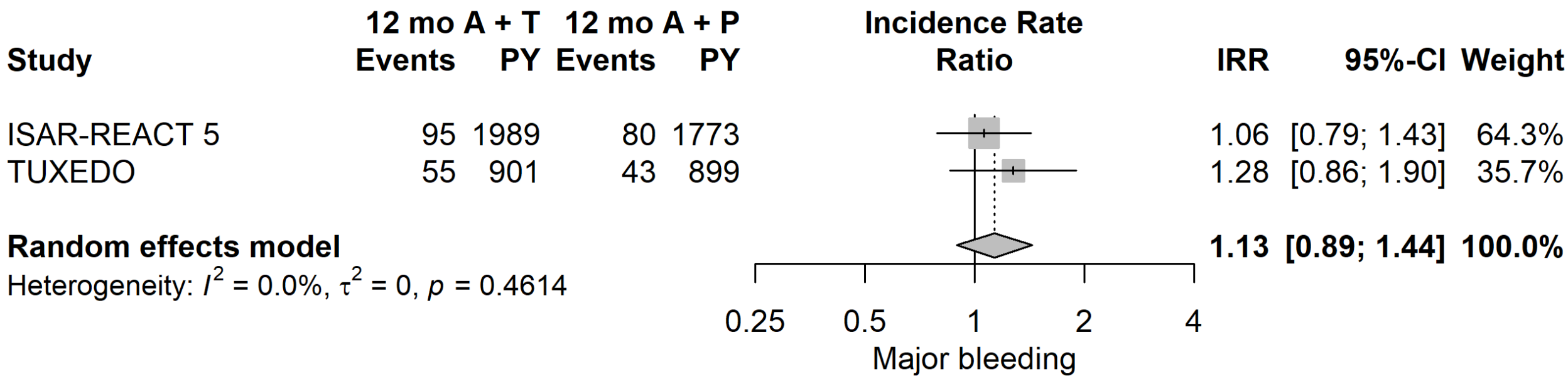

Supplementary Figure 4C . Random-Effects Network Meta-analysis Comparing Ticagrelor-Based Strategies With 12 Months of Aspirin Plus Prasugrel (Reference Treatment)

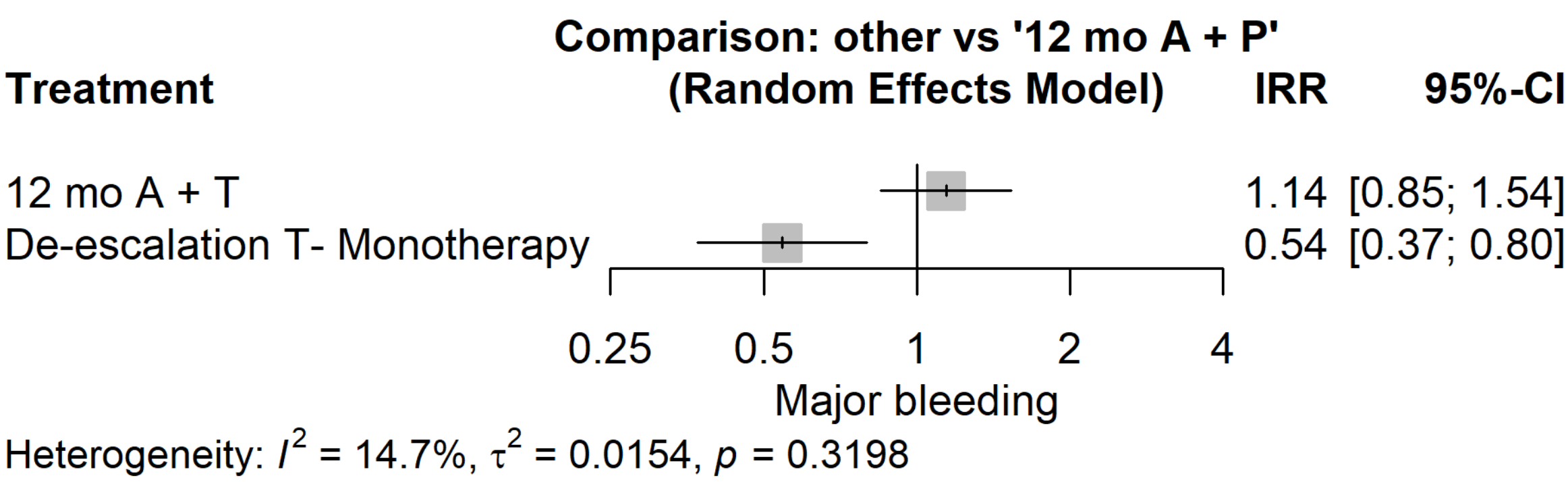

Supplementary Table 3. Study-Level Event Counts and Effect Estimates Used for the Network Meta-analysis

**Study Acronym** (First Author, Year)	**Arms**	**Sample Size**	**Endpoints**													
			**Primary Efficacy (MACCE)**		**Primary Safety (Major Bleeding)**		**All-Cause Death**		**Cardiac Death**		**Myocardial Infarction**		**Stroke**		**Stent thrombosis**	
			**Events**	**HR (95 % CIs)**	**Events**	**HR (95 % CIs)**	**Events**	**HR (95 % CIs)**	**Events**	**HR (95 % CIs)**	**Events**	**HR (95 % CIs)**	**Events**	**HR (95 % CIs)**	**Events**	**HR (95 % CIs)**
**GLOBAL LEADERS-ACS** (Tomaniak et al., 2019)[1] ITT analysis (31-365 days)	De-escalation to Ticagrelor Monotherapy	3750	67/3697	0.79 (0.57-1.08)	28/3680	0.52 (0.33-0.81)	38/3729	0.74 (0.49-1.13)	NA	NA	50/3660	0.96 (0.65-1.42)	17/3697	1.21 (0.60-2.46)	7/3679	1.17 (0.39-3.47)
	Aspirin + Ticagrelor	3737	85/3680		54/3666		51/3712				52/3660		14/3683		6/3679	
**TICO** (Kim et al., 2020)[2] ITT analysis (30-365 days)	De-escalation to Ticagrelor Monotherapy	1527	35	0.69 (0.45-1.06)	25	0.56 (0.34-0.91)	16	0.70 (0.37-1.32)	7	NA	6	0.55 (0.20-1.48)	8	0.73 (0.29-1.81)	6	1.51 (0.43-5.33)
	Aspirin + Ticagrelor	1529	51		45		23		12		11		11		4	
**ULTIMATE-DAPT** (Ge et al., 2024)[5] ITT analysis (1 year after randomization)	De-escalation to Ticagrelor Monotherapy	1700	61	0.98 (0.69-1·39)	11	0.39 (0.19-0·79)	12	0.93 (0.42-2.03)	8	1.15 (0.42-3.18)	17	1.45 (0.67-3.23)	20	0.83 (0.46-1.50)	5	0.97 (0.28-3.40)
	Aspirin + Ticagrelor	1700	63		28		13		7		11		24		5	
**T-PASS** (Hong et al., 2023)[4] ITT analysis (1-365 days)	De-escalation to Ticagrelor Monotherapy	1426	21	0.68 (0.39-1.18)	17	0.35 (0.20-0.61)	14	1.00 (0.48-2.10)	6	0.67 (0.24-1.88)	7	0.88 (0.32-2.41)	8	0.73 (0.29-1.81)	2	1.00 (0.14-7.09)
	Aspirin + Ticagrelor	1424	31		48		14		9		8		11		2	

**Study Acronym**<br>(First Author, Year)	**Arms**	**Sample Size**	**Endpoints**													
			**Primary Efficacy (MACCE)**		**Primary Safety (Major Bleeding)**		**All-Cause Death**		**Cardiac Death**		**Myocardial Infarction**		**Stroke**		**Stent thrombosis**	
			**Events**	**HR (95 % CIs)**	**Events**	**HR (95 % CIs)**	**Events**	**HR (95 % CIs)**	**Events**	**HR (95 % CIs)**	**Events**	**HR (95 % CIs)**	**Events**	**HR (95 % CIs)**	**Events**	**HR (95 % CIs)**
**TWILIGHT-ACS**<br>(Baber et al., 2020)[3]<br><br>ITT<br>(1 year after randomization)	De-escalation to Ticagrelor Monotherapy	2273	96	0.97 (0.74-1.28)	17	0.36 (0.20-0.62)	22	0.67 (0.39-1.14)	NA	NA	70	1.00 (0.72-1.39)	11	1.89 (0.70-5.10)	8	0.59 (0.25-1.40)
	Aspirin + Ticagrelor	2341	102		49		34				72		6		14	
**ISAR-REACT 5**<br>(Schupke et al., 2019)[6]<br><br>ITT analysis<br>(7-365 days)	Aspirin + Ticagrelor	2012	184	1.36 (1.09-1.70)	95°	1.12 (0.83-1.51)	90	1.23 (0.91-1.68)	63	NA	96	1.63 (1.18-2.25)	22	1.17 (0.63-2.15	26	1.30 (0.72-2.33)
	Aspirin + Prasugrel	2006	137		80°		73		59		60		19		20	
**TUXEDO-2**<br>(Bangalore et al., 2026)[7]<br><br>ITT<br>(1-365 days)	Aspirin + Ticagrelor	901	90	1.17 (0.87-1.59)	55	1.31 (0.88-1.95)	41	1.25 (0.79-1.98)	34	1.22 (0.74-2.01)	52[¥]	1.13 (0.76-1.68)	4	0.67 (0.19-2.38)	10	2.00 (0.68-5.85)
	Aspirin + Prasugrel	899	77		43		33		28		46		6		5	

MACCE=Major Adverse Cardiac and Cerebrovascular Events; ST= stent thrombosis; ITT=intention to treat analysis; PP=per protocol

[¥]Reported as Nonfatal MI
[1]Reported as Rate Ratio in the original study.
° Sample size for the Major Bleeding respectively 1989 and 1773

Supplementary Figure 5. Pairwise and Network Meta-analyses for all-cause death

Supplementary Figure 5A. Pairwise Meta-analysis of De-escalation to Ticagrelor Monotherapy vs 12 Months of Ticagrelor-Based DAPT

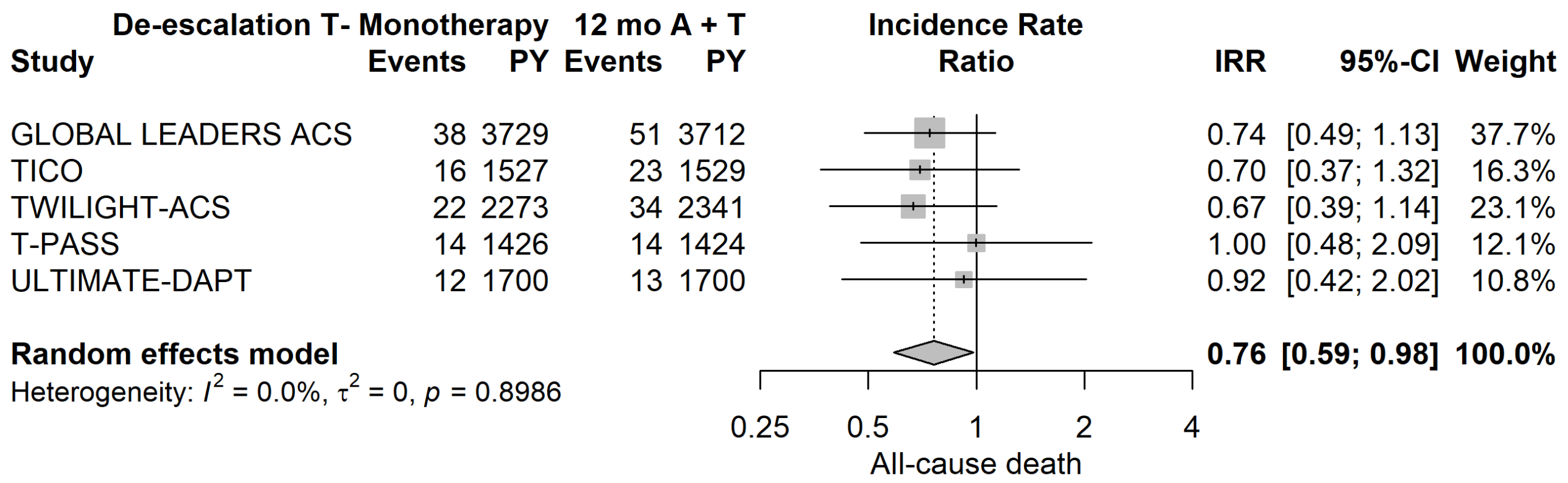

Supplementary Figure 5B . Pairwise Meta-analysis of 12 Months of Aspirin Plus Ticagrelor vs 12 Months of Aspirin Plus Prasugrel

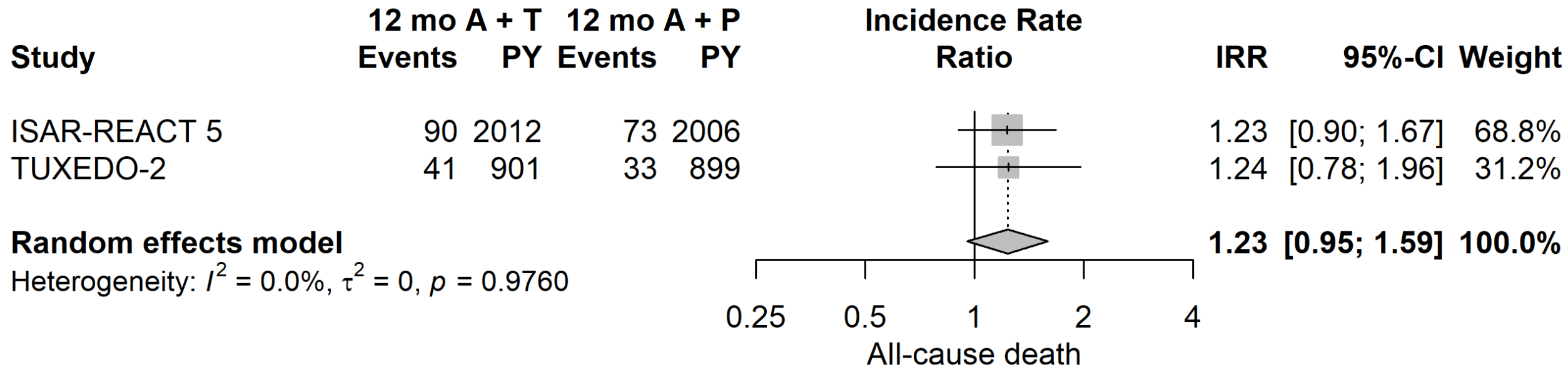

Supplementary Figure 5C . Random-Effects Network Meta-analysis Comparing Ticagrelor-Based Strategies With 12 Months of Aspirin Plus Prasugrel (Reference Treatment)

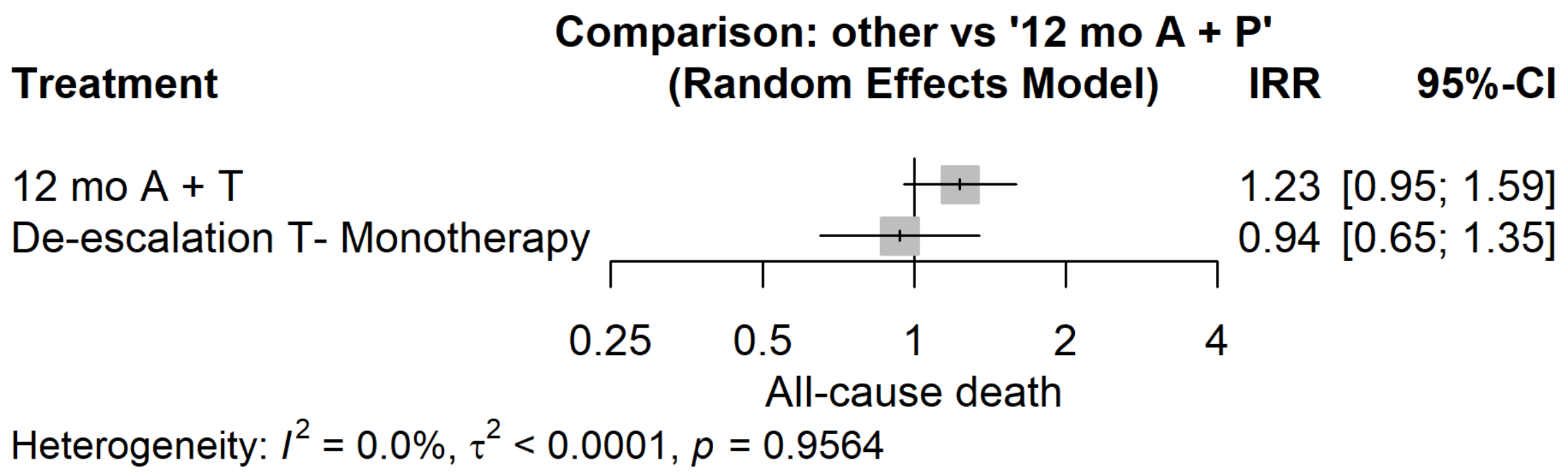

Supplementary Figure 5D. League table

12 mo of DAPT (A + P)		
1.23 [0.95; 1.59]	12 mo of DAPT (A + T)	
0.94 [0.65; 1.35]	0.76 [0.59; 0.98]*	1–3 mo of DAPT followed by ticagrelor monotherapy

Supplementary Figure 6. Pairwise and Network Meta-analyses for cardiac death

Supplementary Figure 6A. Pairwise Meta-analysis of De-escalation to Ticagrelor Monotherapy vs 12 Months of Ticagrelor-Based DAPT

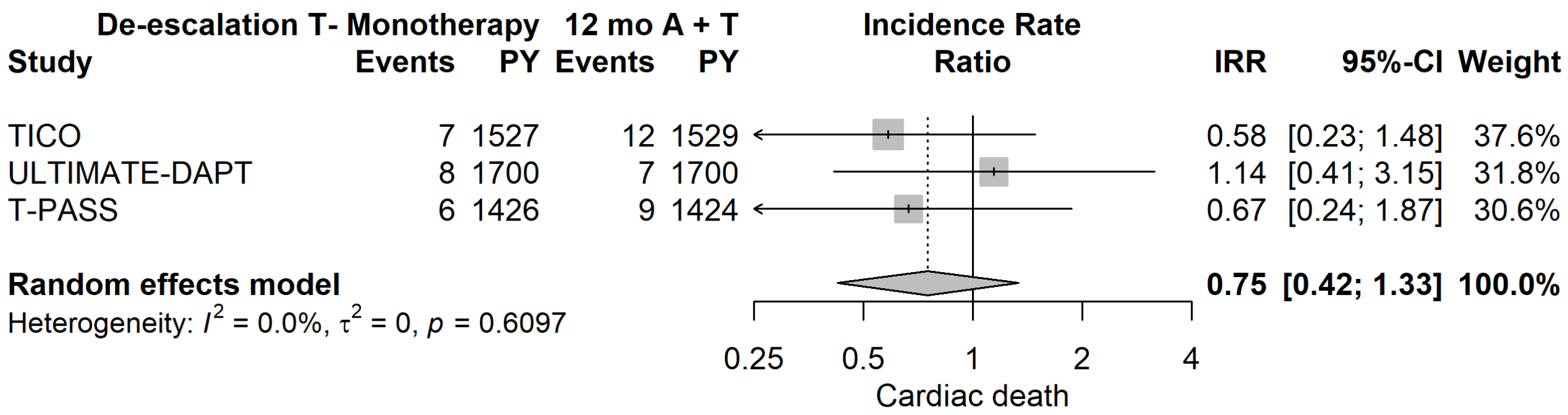

Supplementary Figure 6B . Pairwise Meta-analysis of 12 Months of Aspirin Plus Ticagrelor vs 12 Months of Aspirin Plus Prasugrel

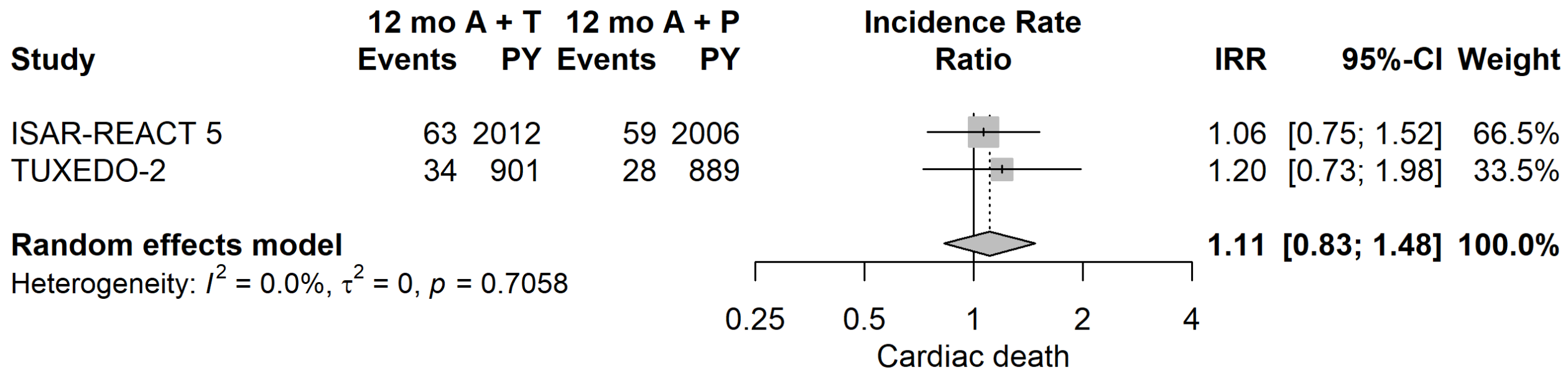

Supplementary Figure 6C . Random-Effects Network Meta-analysis Comparing Ticagrelor-Based Strategies With 12 Months of Aspirin Plus Prasugrel (Reference Treatment)

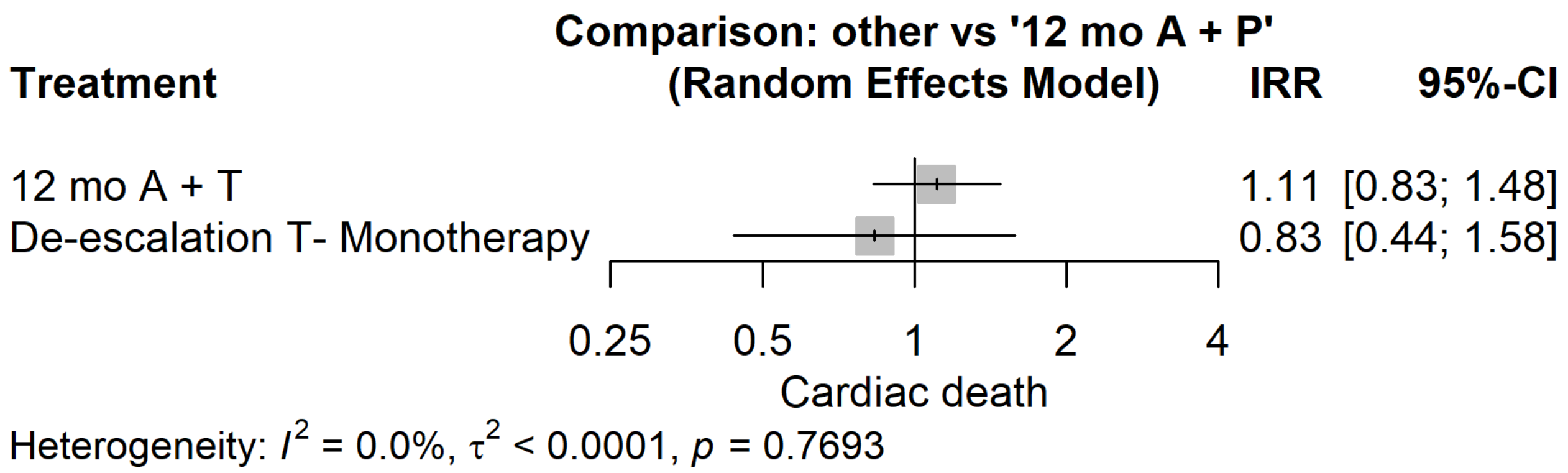

Supplementary Figure 6D. League table

12 mo of DAPT (A + P)		
1.11 [0.83; 1.48]	12 mo of DAPT (A + T)	
0.83 [0.44; 1.58]	0.75 [0.42; 1.33]	1–3 mo of DAPT followed by ticagrelor monotherapy

Supplementary Figure 7. Pairwise and Network Meta-analyses for myocardial infarction

Supplementary Figure 7A. Pairwise Meta-analysis of De-escalation to Ticagrelor Monotherapy vs 12 Months of Ticagrelor-Based DAPT

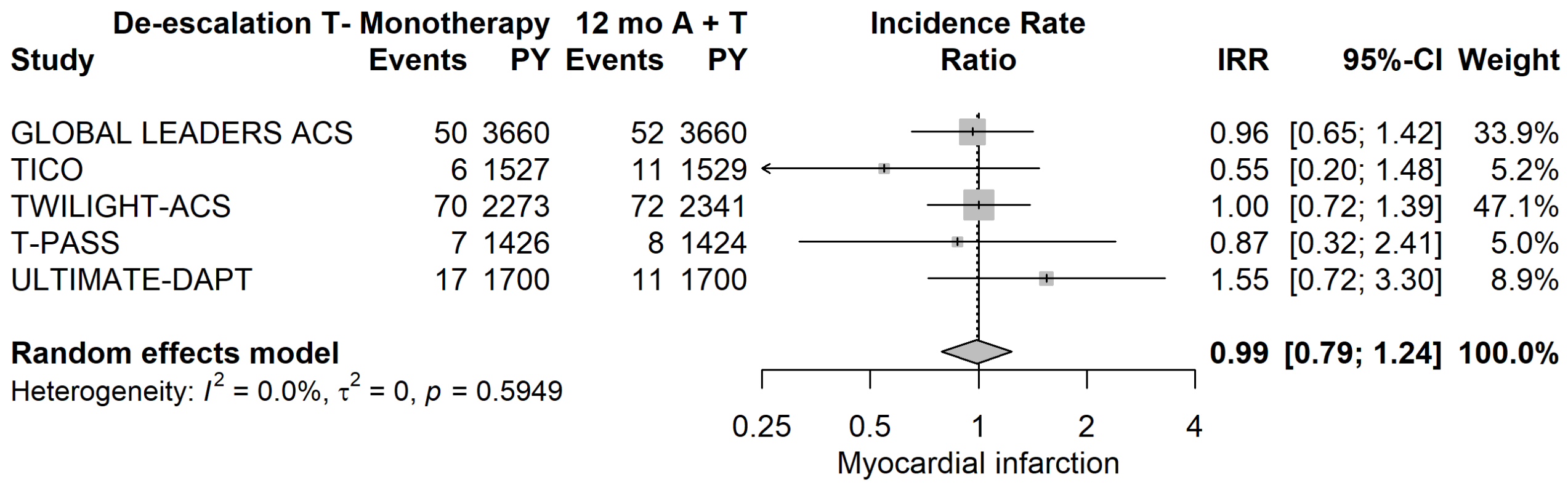

Supplementary Figure 7B . Pairwise Meta-analysis of 12 Months of Aspirin Plus Ticagrelor vs 12 Months of Aspirin Plus Prasugrel

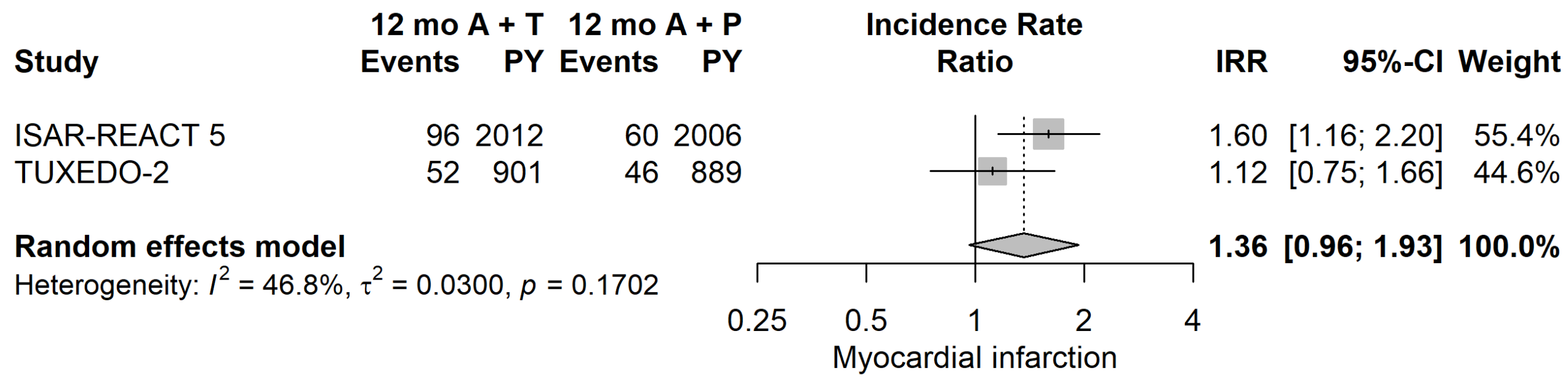

Supplementary Figure 7C . Random-Effects Network Meta-analysis Comparing Ticagrelor-Based Strategies With 12 Months of Aspirin Plus Prasugrel (Reference Treatment)

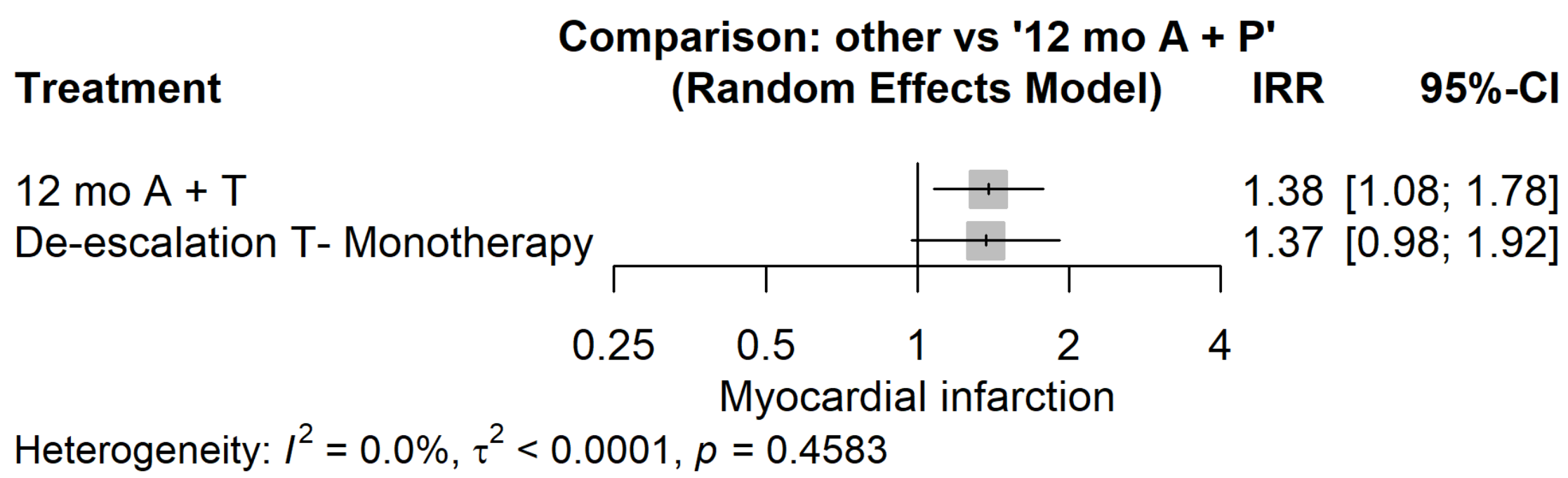

Supplementary Figure 7D. League table

12 mo of DAPT (A + P)		
1.38 [1.08; 1.78]*	12 mo of DAPT (A + T)	
1.37 [0.98; 1.92]	0.99 [0.79; 1.24]	1–3 mo of DAPT followed by ticagrelor monotherapy

Supplementary Figure 8. Pairwise and Network Meta-analyses for stroke

Supplementary Figure 8A. Pairwise Meta-analysis of De-escalation to Ticagrelor Monotherapy vs 12 Months of Ticagrelor-Based DAPT

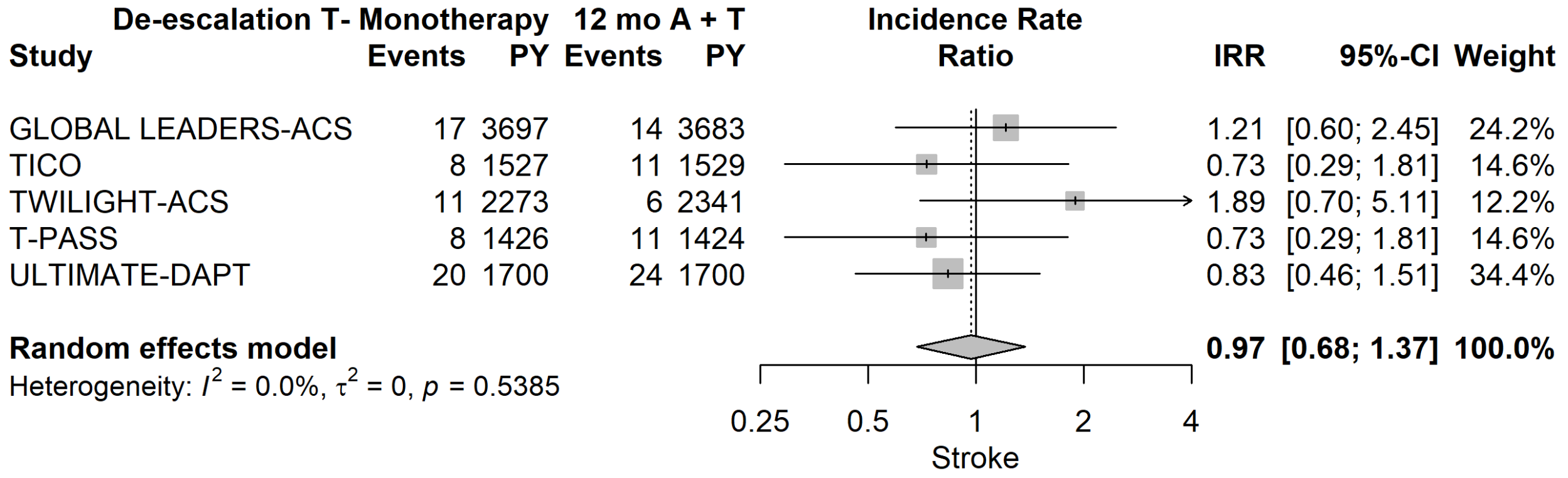

Supplementary Figure 8B . Pairwise Meta-analysis of 12 Months of Aspirin Plus Ticagrelor vs 12 Months of Aspirin Plus Prasugrel

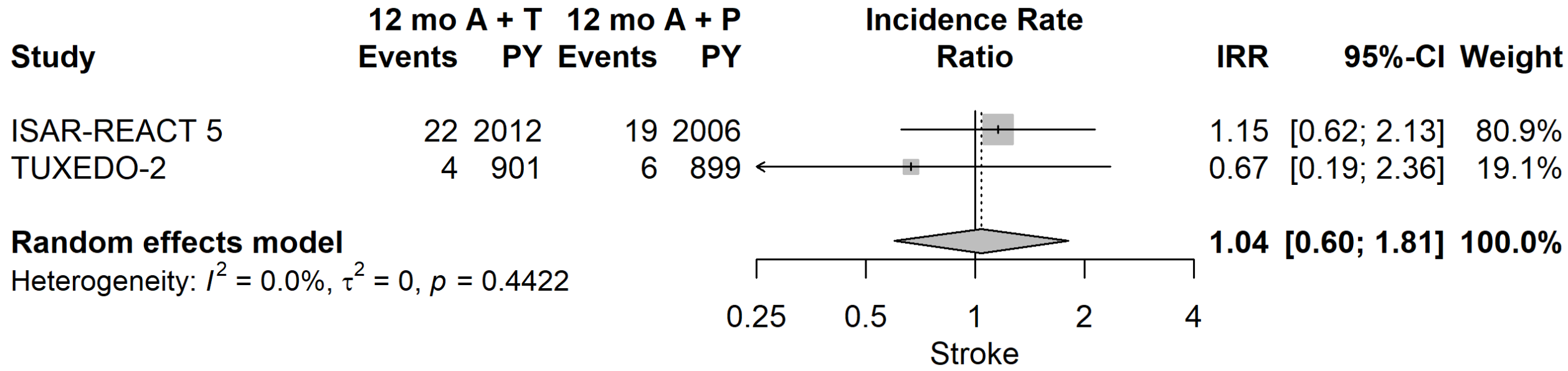

Supplementary Figure 8C . Random-Effects Network Meta-analysis Comparing Ticagrelor-Based Strategies With 12 Months of Aspirin Plus Prasugrel (Reference Treatment)

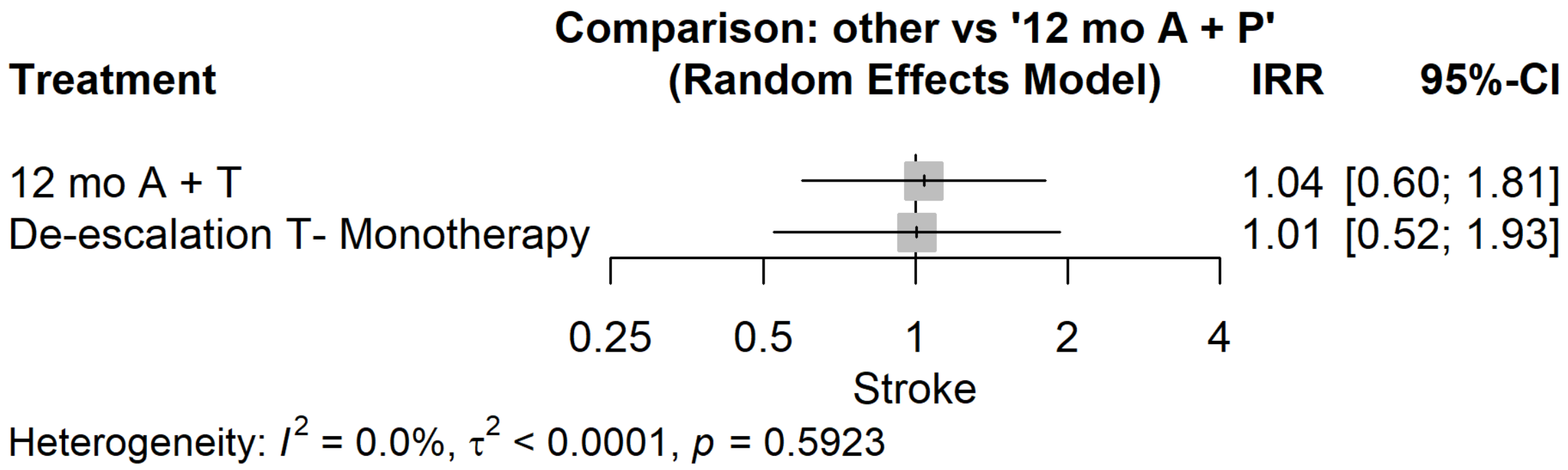

Supplementary Figure 8D. League table

12 mo of DAPT (A + P)		
1.04 [0.60; 1.81]	12 mo of DAPT (A + T)	
1.01 [0.52; 1.93]	0.97 [0.68; 1.37]	1–3 mo of DAPT followed by ticagrelor monotherapy

Supplementary Figure 9. Pairwise and Network Meta-analyses for stent thrombosis

Supplementary Figure 9A. Pairwise Meta-analysis of De-escalation to Ticagrelor Monotherapy vs 12 Months of Ticagrelor-Based DAPT

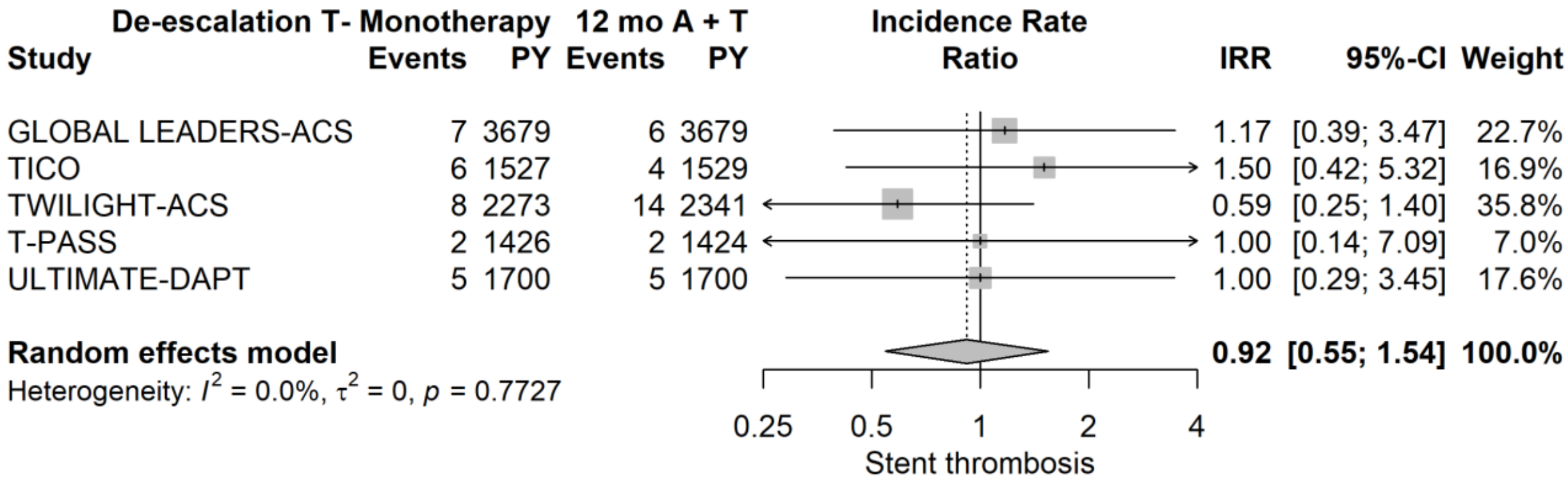

Supplementary Figure 9B . Pairwise Meta-analysis of 12 Months of Aspirin Plus Ticagrelor vs 12 Months of Aspirin Plus Prasugrel

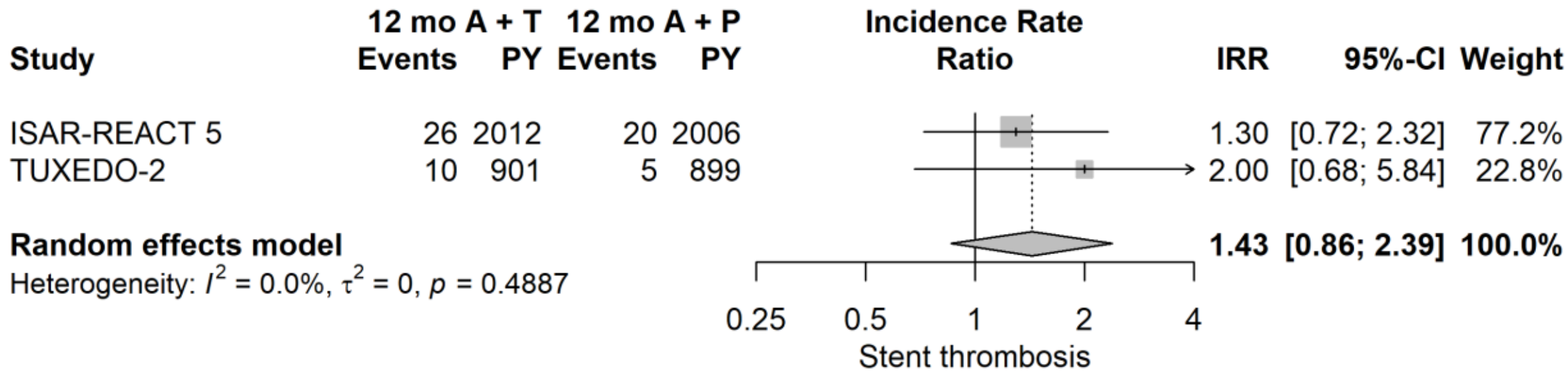

Supplementary Figure 9C . Random-Effects Network Meta-analysis Comparing Ticagrelor-Based Strategies With 12 Months of Aspirin Plus Prasugrel (Reference Treatment)

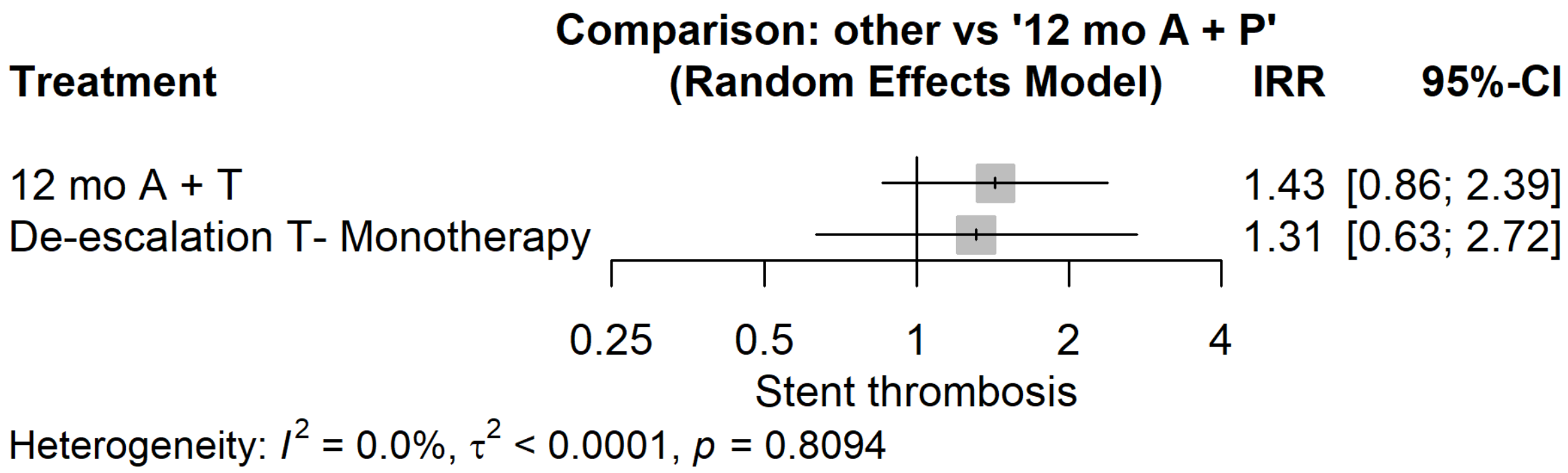

Supplementary Figure 9D. League table

12 mo of DAPT (A + P)		
1.43 [0.86; 2.39]	12 mo of DAPT (A + T)	
1.31 [0.63; 2.72]	0.92 [0.55; 1.54]	1–3 mo of DAPT followed by ticagrelor monotherapy

Supplementary Figure 10. Ranking probabilities (rankograms) for primary and secondary endpoints

Supplementary Figure 10A. MACCE

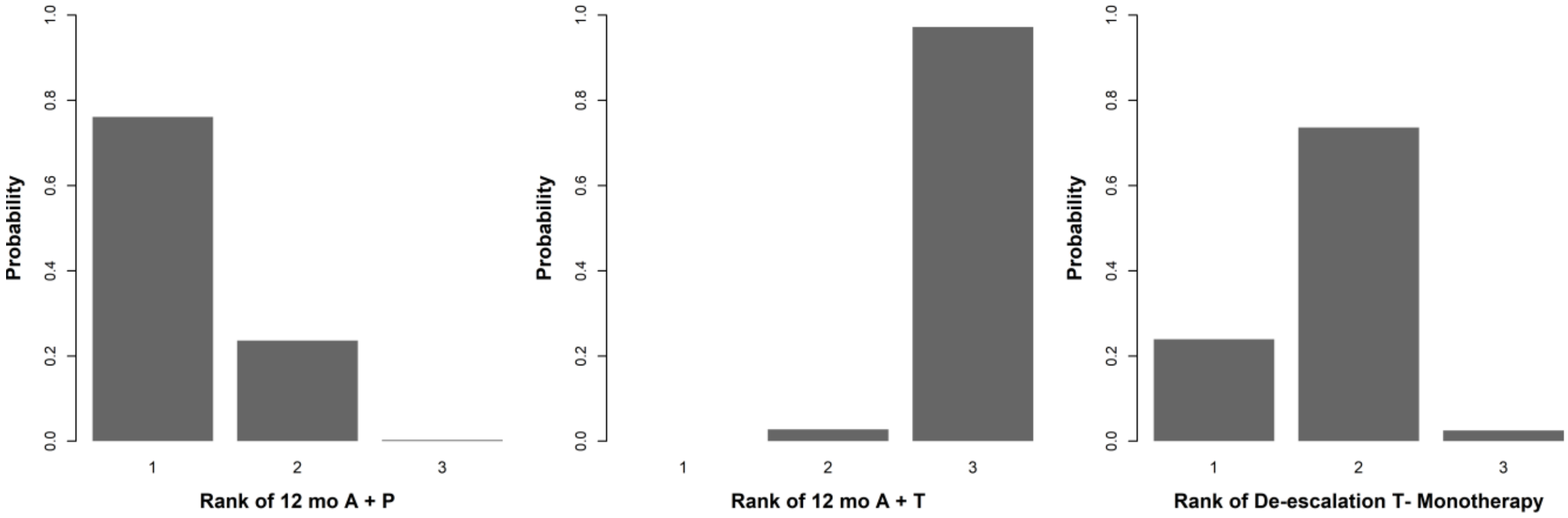

Supplementary Figure 10B. Major bleeding

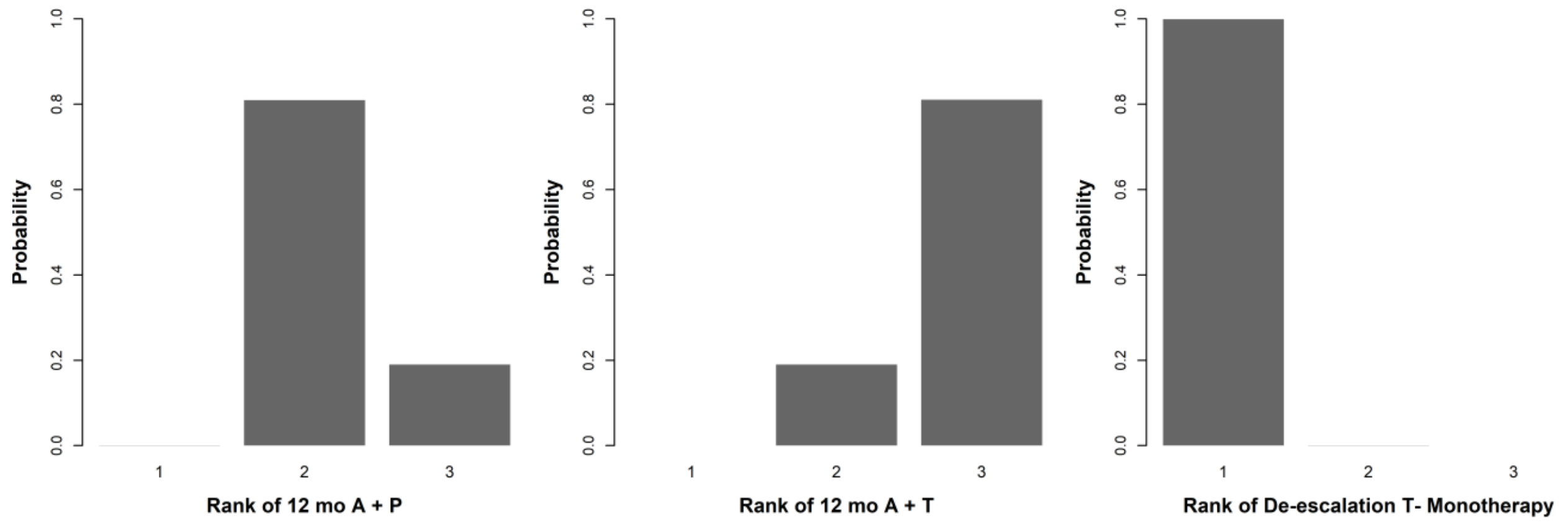

Supplementary Figure 10C. All-cause death

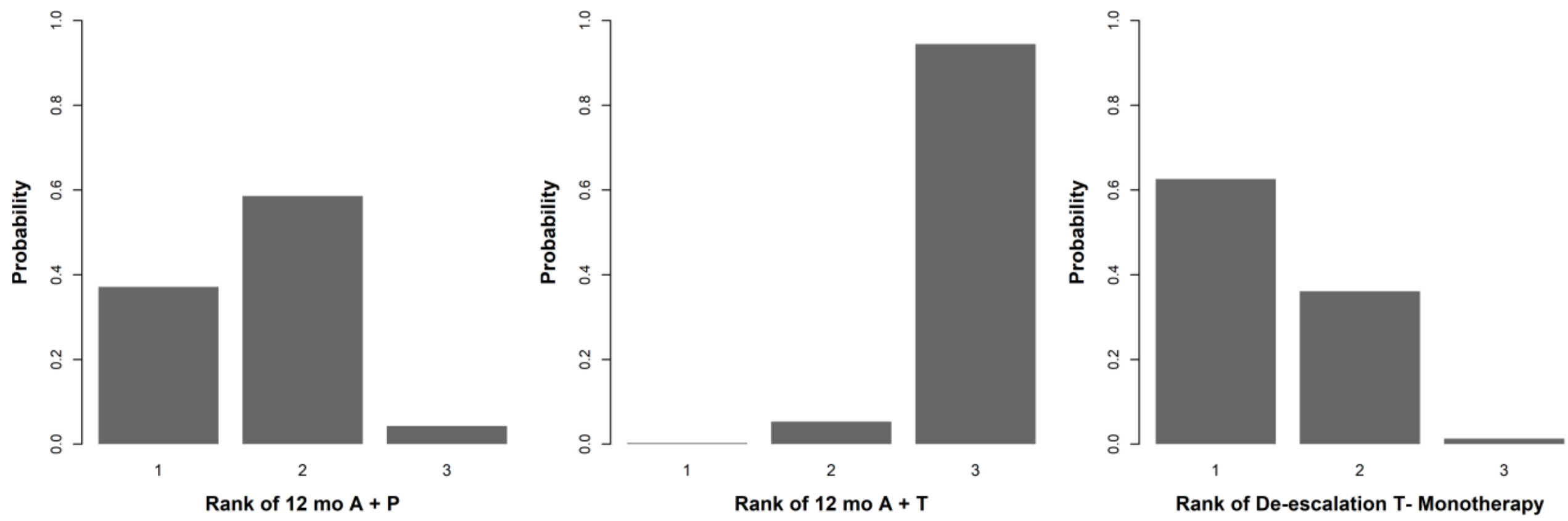

Supplementary Figure 10D. Cardiac death

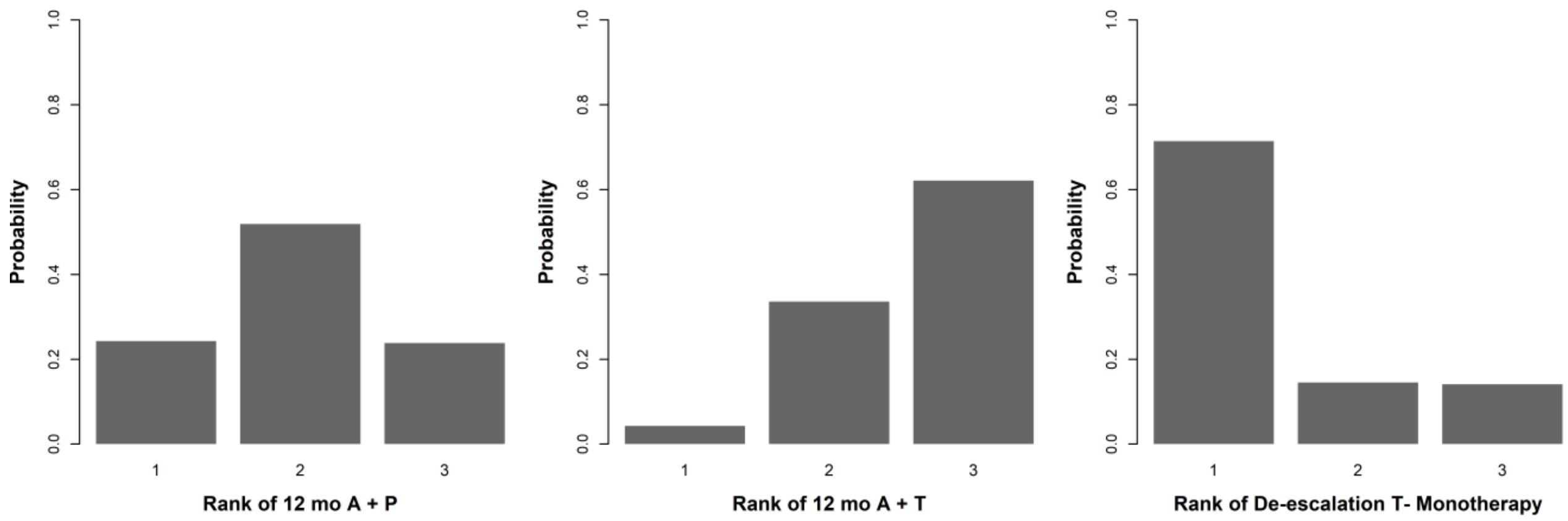

Supplementary Figure 10E. Myocardial infarction

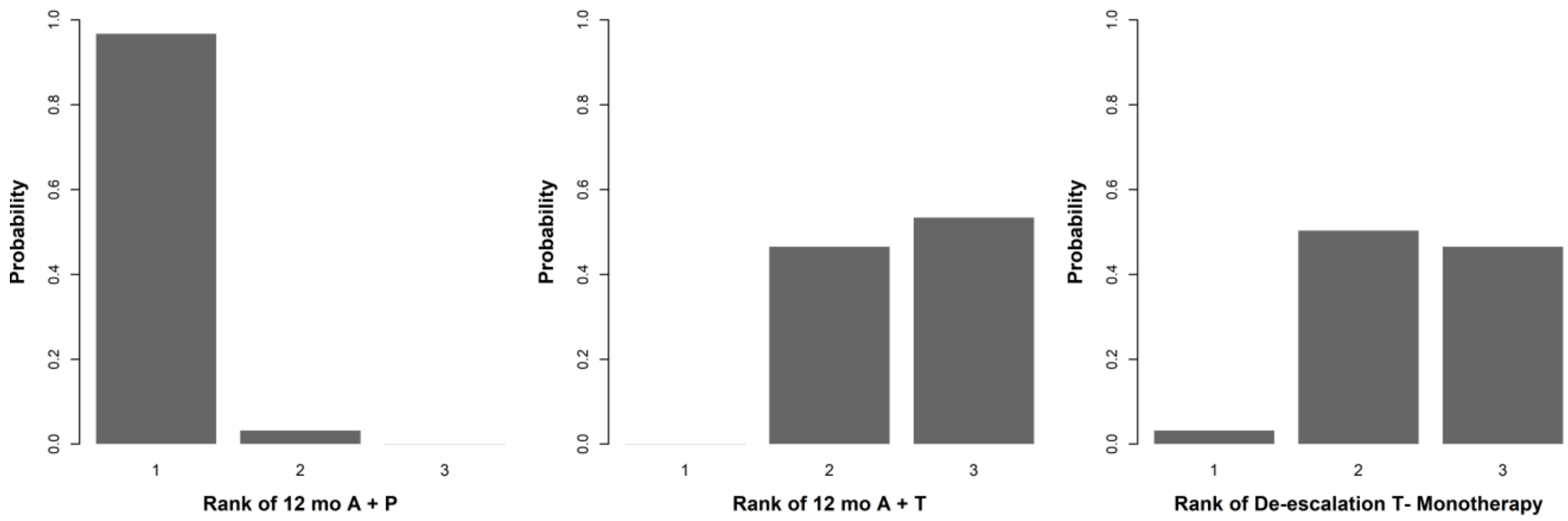

Supplementary Figure 10F. Stroke

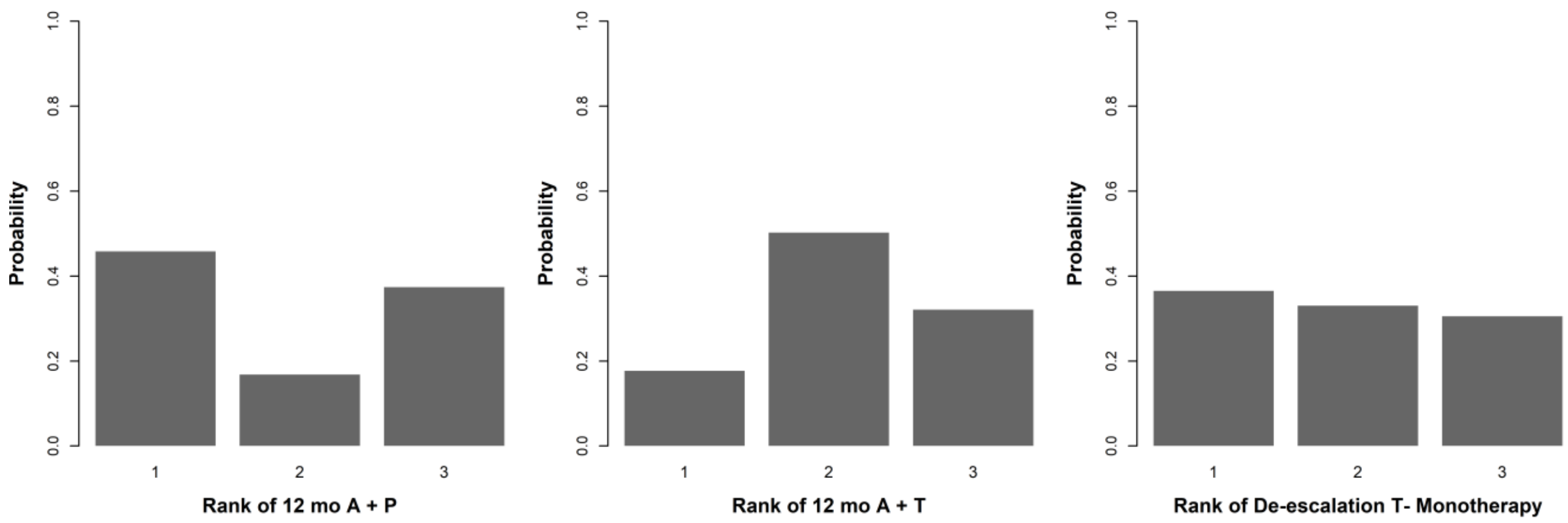

Supplementary Figure 10G. Stent thrombosis

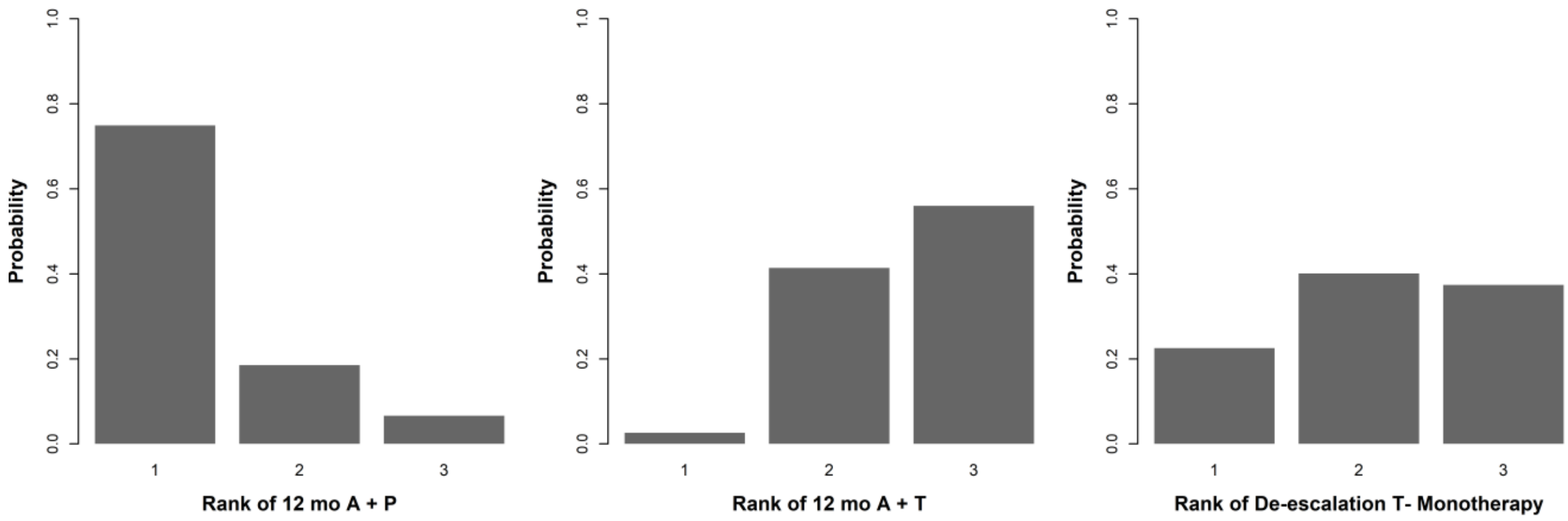

Supplementary Figure 11. Relationship Between Major Bleeding and All-Cause Death

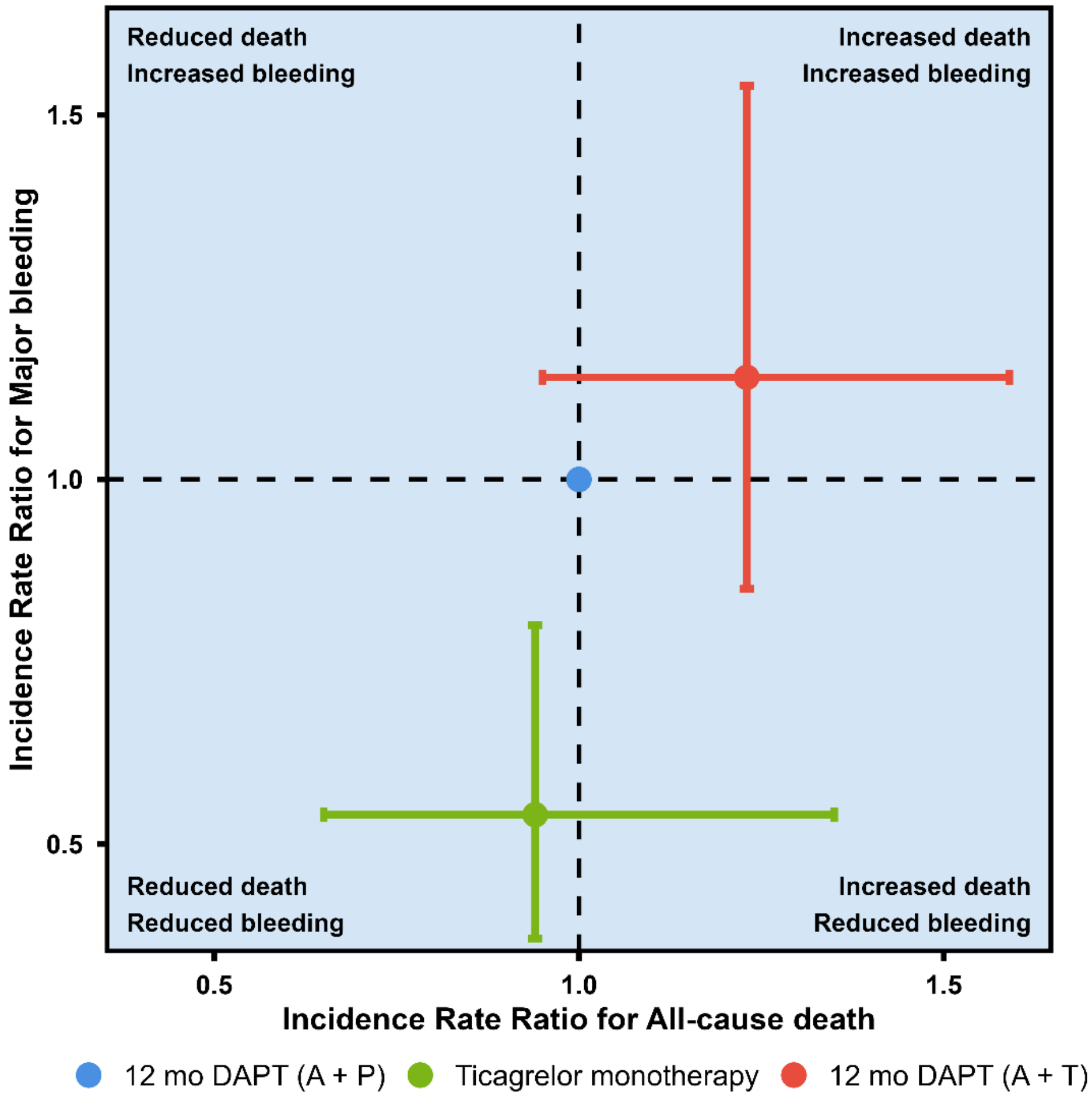

Incidence rate ratios for all-cause death (x-axis) and major bleeding (y-axis) across antiplatelet strategies relative to 12 months of aspirin plus prasugrel (reference) with corresponding 95% confidence intervals. Dashed lines represent the line of no effect (IRR = 1). The plot illustrates the relationship between bleeding and mortality risk across treatment strategies.

Supplementary Results 1. Consistency and Heterogeneity Assessment

Because the network contained no closed treatment loops and no comparison was informed by both direct and indirect evidence, global and local inconsistency could not be formally assessed; accordingly, the design-by-treatment interaction and node-splitting approaches were not informative. However, heterogeneity was low across all endpoints. For MACCE, heterogeneity was negligible ($Q = 3.73$, $df = 5$, $P = 0.59$; $\tau^2 < 0.0001$; $I^2 = 0\%$). For major bleeding, heterogeneity was low ($Q = 5.86$, $df = 5$, $P = 0.32$; $\tau^2 = 0.015$; $I^2 = 14.7\%$). Heterogeneity was negligible for all-cause death ($Q = 1.07$, $df = 5$, $P = 0.96$; $\tau^2 < 0.0001$; $I^2 = 0\%$), cardiac death ($Q = 1.13$, $df = 3$, $P = 0.77$; $\tau^2 < 0.0001$; $I^2 = 0\%$), myocardial infarction ($Q = 4.66$, $df = 5$, $P = 0.46$; $\tau^2 < 0.0001$; $I^2 = 0\%$), stroke ($Q = 3.71$, $df = 5$, $P = 0.59$; $\tau^2 < 0.0001$; $I^2 = 0\%$), and stent thrombosis ($Q = 2.28$, $df = 5$, $P = 0.81$; $\tau^2 < 0.0001$; $I^2 = 0\%$).

Supplementary Table 4. Contribution Matrix (direct and indirect) for the Network Meta-analysis (All End Points)

Network Comparison	12 mo A + T vs 12 mo A + P	De-escalation T-Monotherapy vs 12 mo A + T
**12 mo A + T vs 12 mo A + P**	1.00	0.00
**De-escalation T-Monotherapy vs 12 mo A + P**	0.50<br><br>32.7% ISAR-REACT 5<br>17.3% TUXEDO-2	0.50<br><br>GLOBAL LEADERS-ACS: 12.4%<br>TICO: 6.9%<br>TWILIGHT-ACS: 16.4%<br>T-PASS: 4.1%<br>ULTIMATE-DAPT: 10.2%
**De-escalation T-Monotherapy vs 12 mo A + T**	0.00	1.00

Values represent the proportion of information contributed by each direct comparison to the corresponding network estimate, based on the random-effects model (Papakonstantinou et al., 2018). The comparison between 12 months of aspirin plus prasugrel and 12 months of aspirin plus ticagrelor was informed exclusively by direct evidence (100%), as was the comparison between 12 months of aspirin plus ticagrelor and de-escalation to ticagrelor monotherapy (100%). The indirect comparison between ticagrelor monotherapy and aspirin plus prasugrel received equal contributions from the two direct comparisons forming the network pathway (50% each). For this indirect comparison, percentages reported beneath each contribution represent the study-level decomposition of the corresponding 50% contribution according to random-effects study weights. Thus, ISAR-REACT 5 and TUXEDO-2 contributed 32.7% and 17.3%, respectively, to the overall indirect estimate; percentages for the five ticagrelor-monotherapy trials similarly sum to the remaining 50%.

Supplementary Results 2. Sensitivity Analysis

Sensitivity analyses yielded results consistent with the primary analysis for MACCE. Restricting the network to trials enrolling patients with ACS did not materially alter the ranking of treatment strategies, with 12 months of aspirin plus prasugrel remaining the highest-ranked strategy and aspirin plus ticagrelor consistently ranking lowest. Notably, in the ACS-restricted analysis, aspirin plus ticagrelor was associated with a significantly higher risk of MACCE compared with aspirin plus prasugrel (IRR 1.34, 95% CI 1.07–1.67), while ticagrelor monotherapy showed no significant differences compared with prasugrel-based DAPT. Similar findings were observed after excluding East-Asian populations and when restricting the analysis to trials with 3 months of DAPT before de-escalation to ticagrelor monotherapy. Sensitivity analyses using a common-effect model and pooled hazard ratios from time-to-event analyses also confirmed the robustness of the primary findings (Supplementary Figures 12–16). Sensitivity analyses for major bleeding were consistent with the primary analysis. Restricting the network to trials enrolling patients with ACS did not materially alter treatment ranking, with de-escalation to ticagrelor monotherapy remaining the top-ranked strategy and showing a significant reduction in major bleeding compared with both 12 months of aspirin plus prasugrel (IRR 0.50, 95% CI 0.29–0.84) and 12 months of aspirin plus ticagrelor (IRR 0.47, 95% CI 0.36–0.61). Similar findings were observed after excluding East Asian populations and across sensitivity analyses according to the timing of aspirin withdrawal, including restriction to trials with ≥3 months of DAPT before de-escalation and exclusion of trials with very early aspirin discontinuation (<1 month after PCI; T-PASS). Sensitivity analyses using a common-effect model and pooled hazard ratios from time-to-event analyses also confirmed the robustness of the primary findings, with ticagrelor monotherapy consistently ranking highest for bleeding reduction. Results are presented in Supplementary Figures 12–21.

In leave-one-out analyses of the direct comparison between 12 months of aspirin plus ticagrelor and 12 months of aspirin plus prasugrel, exclusion of ISAR-REACT 5 yielded an IRR of 0.99 (95% CI, 0.70–1.40) for the indirect comparison of ticagrelor monotherapy versus aspirin plus prasugrel, with

relatively close P-scores for ticagrelor monotherapy and aspirin plus prasugrel (0.746 and 0.662, respectively). Conversely, after exclusion of TUXEDO-2, the corresponding IRR was 1.14 (95% CI, 0.87–1.50), and aspirin plus prasugrel remained the highest-ranked strategy (P-score, 0.911). The change in ranking after exclusion of ISAR-REACT 5 occurred alongside a substantial change in the population informing the ticagrelor-versus-prasugrel comparison, as TUXEDO-2, conducted exclusively in patients with diabetes, became the sole source of direct evidence for this network edge. Given the observed effect modification by diabetes status for the direct ticagrelor-versus-prasugrel comparison (P for interaction=.019), the relative contribution of single-study influence and differences in the population informing this comparison remains uncertain (Supplementary Figures 22 and 23).

Supplementary Figure 12. League Tables and Rankograms for the Sensitivity Analysis of MACCE Restricted to the ACS Population

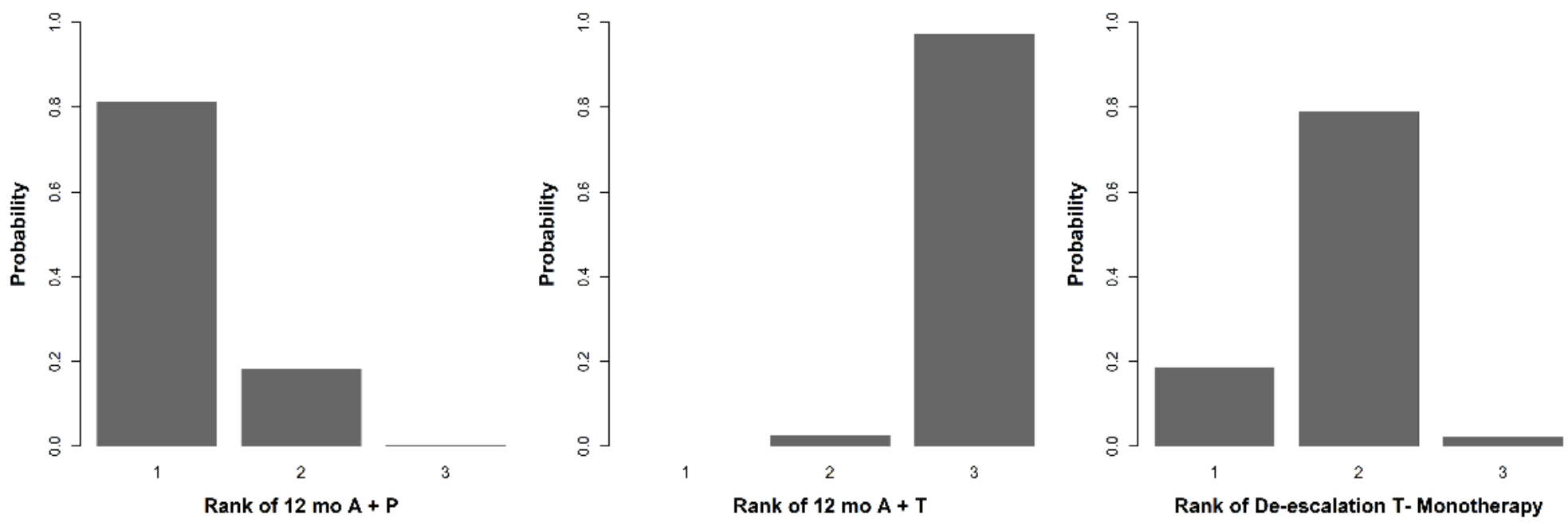

12 mo of DAPT (A + P)		
1.34 [1.07; 1.67]*	12 mo of DAPT (A + T)	
1.14 [0.87; 1.50]	0.85 [0.73; 1.00]	1–3 mo of DAPT followed by ticagrelor monotherapy

Supplementary Figure 13. League Tables and Rankograms for the Sensitivity Analysis of MACCE Restricted to the Non–East Asian Population

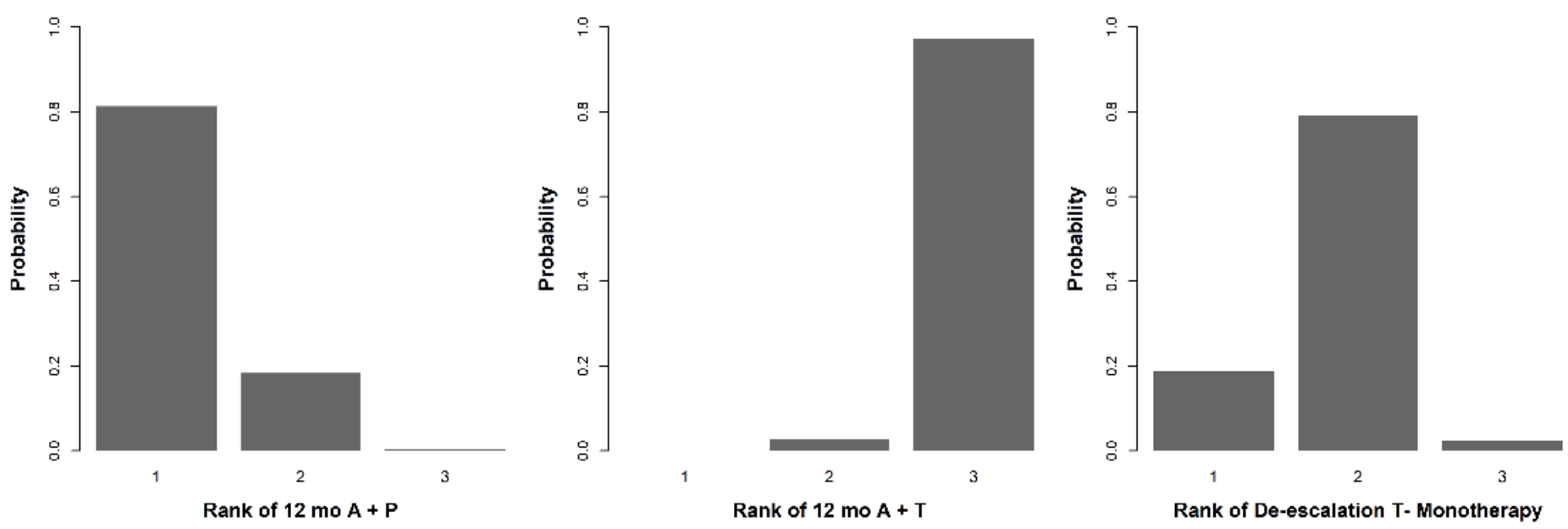

12 mo of DAPT (A + P)		
1.28 [1.07; 1.53]*	12 mo of DAPT (A + T)	
1.13 [0.86; 1.49]	0.88 [0.72; 1.09]	1–3 mo of DAPT followed by ticagrelor monotherapy

Supplementary Figure 14. League tables and rankograms for sensitivity analyses of MACCE according to timing of aspirin withdrawal

Supplementary Figure 14A. Restricted to trials with ≥3 months of DAPT before de-escalation to ticagrelor monotherapy

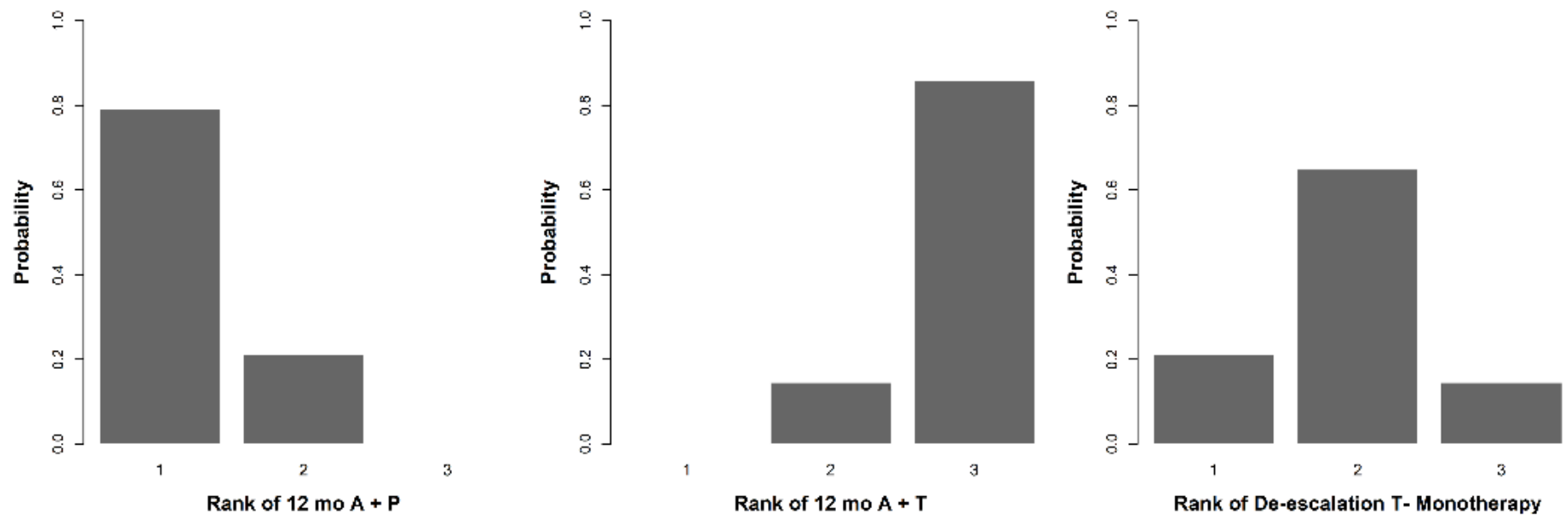

12 mo of DAPT (A + P)		
1.28 [1.07; 1.53] *	12 mo of DAPT (A + T)	
1.12 [0.83; 1.50]	0.88 [0.69; 1.11]	1–3 mo of DAPT followed by ticagrelor monotherapy

Supplementary Figure 14B. Excluding trials with very early aspirin withdrawal (<1 month after PCI; T-PASS)

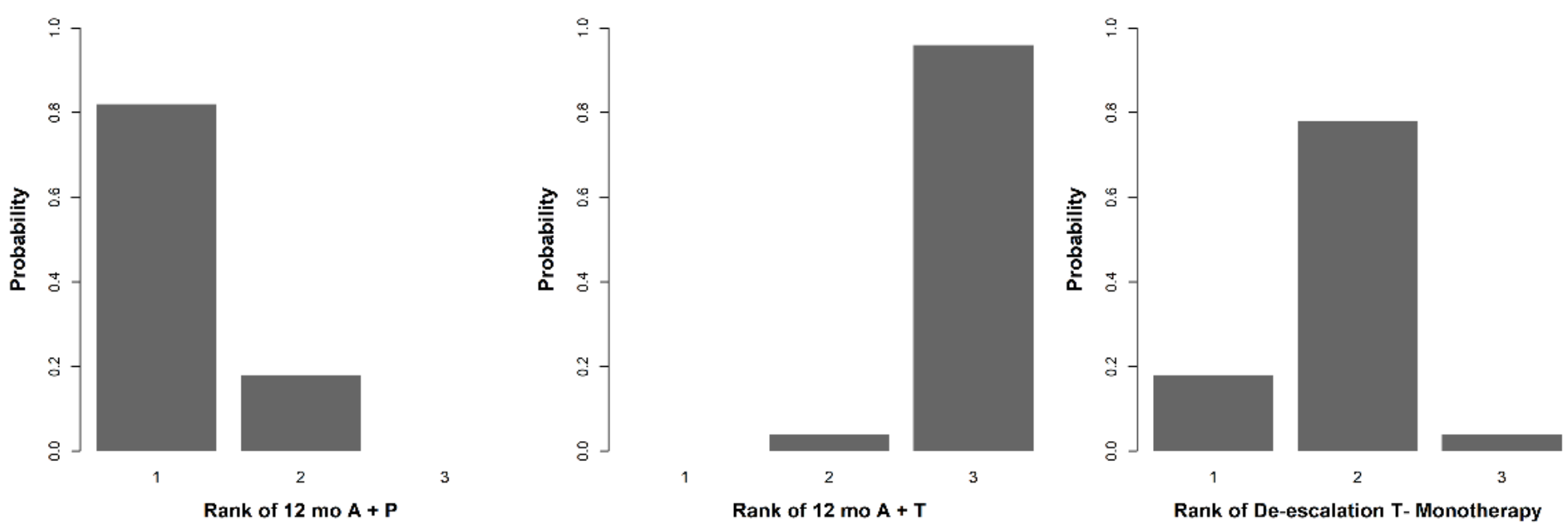

12 mo of DAPT (A + P)		
1.28 [1.07; 1.53]*	12 mo of DAPT (A + T)	
1.11 [0.87; 1.42]	0.87 [0.74; 1.03]	1–3 mo of DAPT followed by ticagrelor monotherapy

Supplementary Figure 15. League Tables and Rankograms for the Sensitivity Analysis of MACCE Using a Common-Effect Model

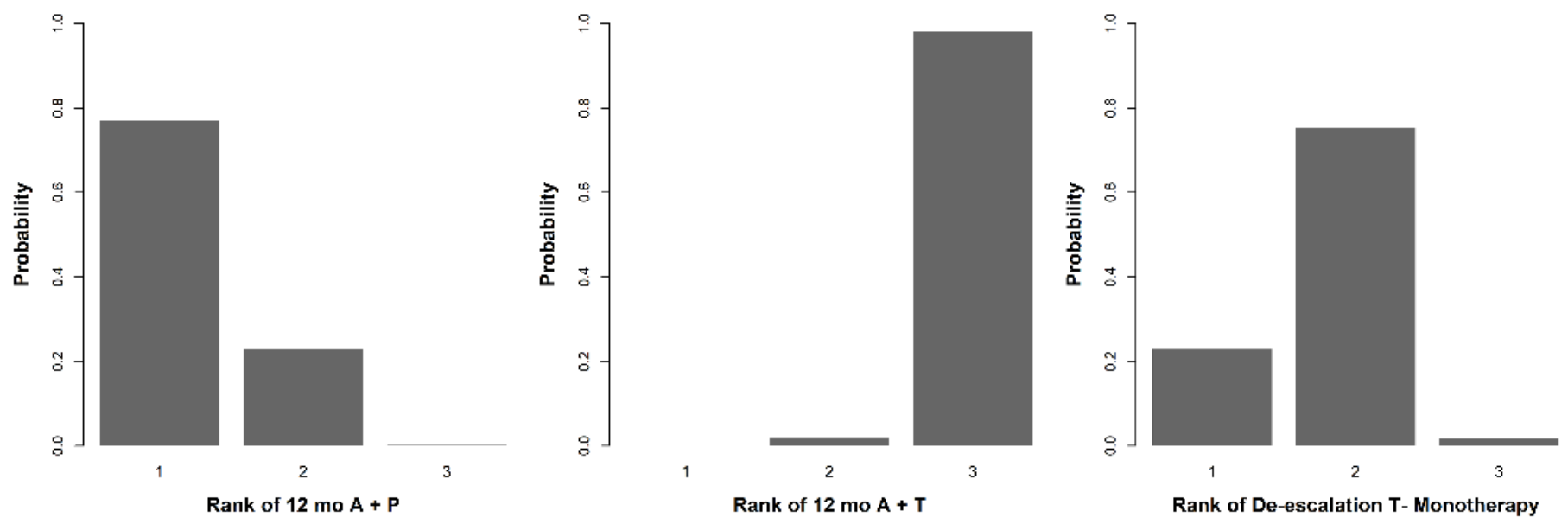

12 mo of DAPT (A + P)		
1.28 [1.07; 1.53]*	12 mo of DAPT (A + T)	
1.09 [0.86; 1.38]	0.85 [0.73; 1.00]	1–3 mo of DAPT followed by ticagrelor monotherapy

Supplementary Figure 16. League Tables and Rankograms for the Sensitivity Analysis of MACCE Using Pooled Hazard Ratios (Time-to-Event Analysis)

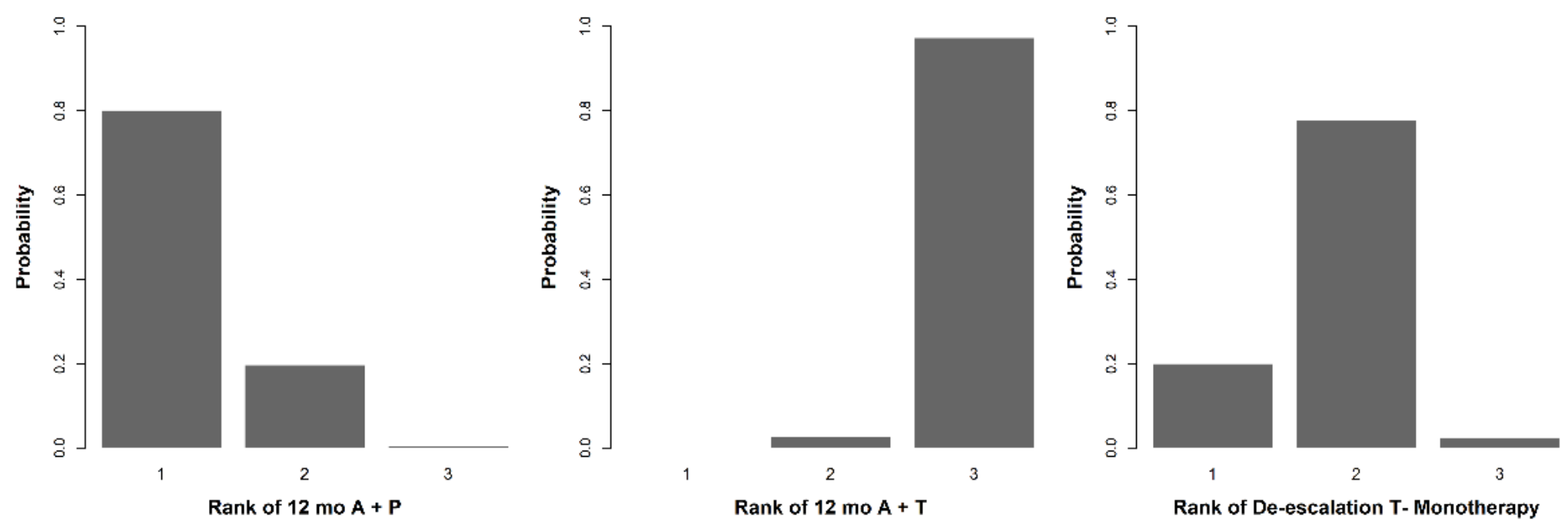

12 mo of DAPT (A + P)		
1.29 [1.08; 1.54]*	12 mo of DAPT (A + T)	
1.11 [0.87; 1.40]	0.86 [0.73; 1.00]	1–3 mo of DAPT followed by ticagrelor monotherapy

Supplementary Figure 17. League Tables and Rankograms for the Sensitivity Analysis of Major Bleeding Restricted to the ACS Population

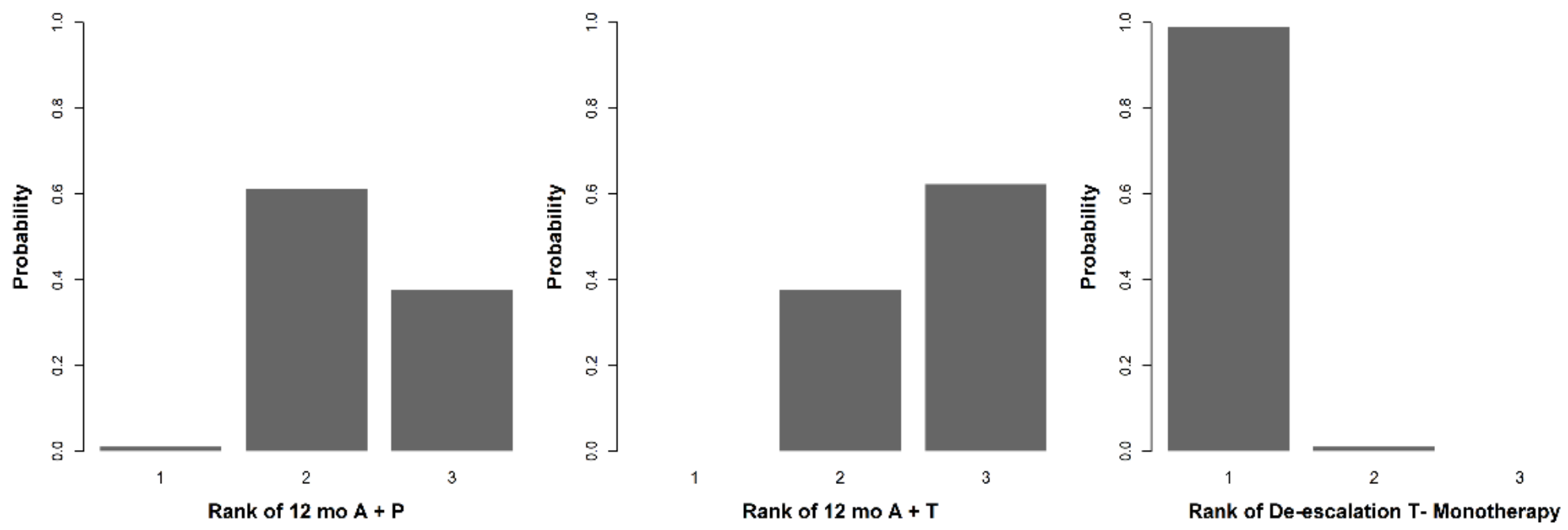

12 mo of DAPT (A + P)		
1.06 [0.67; 1.66]	12 mo of DAPT (A + T)	
0.50 [0.29; 0.84]*	0.47 [0.36; 0.61]*	1–3 mo of DAPT followed by ticagrelor monotherapy

Supplementary Figure 18. League Tables and Rankograms for the Sensitivity Analysis of Major Bleeding Restricted to the Non–East Asian Population

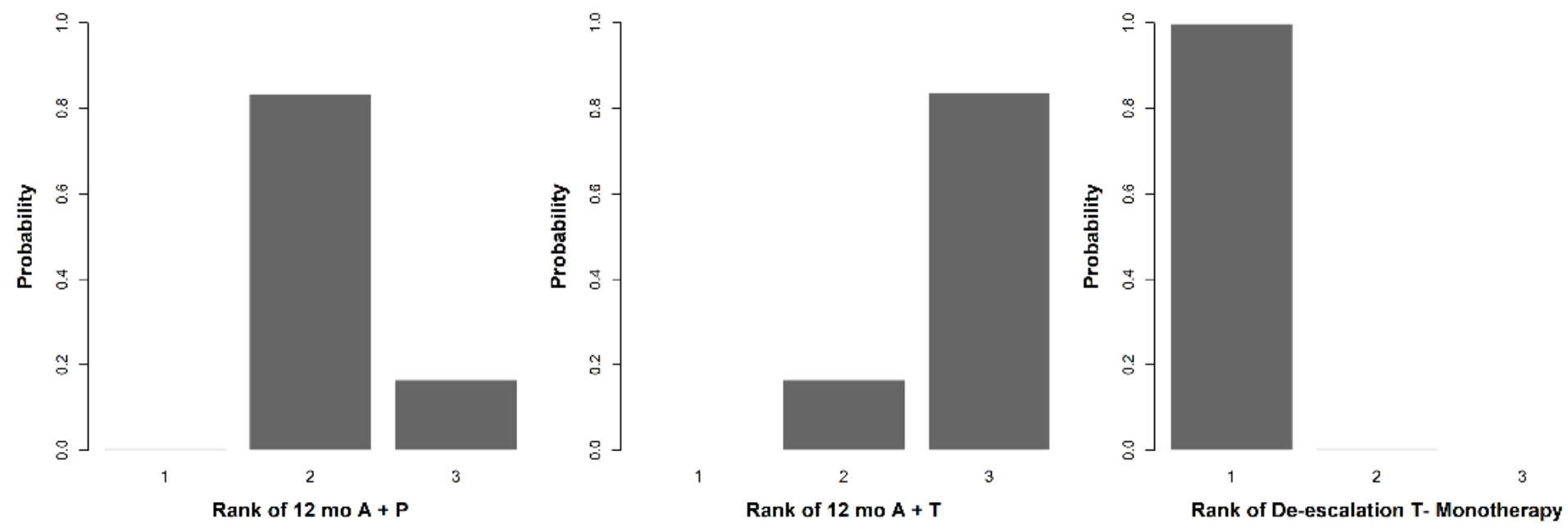

12 mo of DAPT (A + P)		
1.13 [0.89; 1.44]	12 mo of DAPT (A + T)	
0.54 [0.34; 0.84]*	0.48 [0.32; 0.70]*	1–3 mo of DAPT followed by ticagrelor monotherapy

Supplementary Figure 19. League tables and rankograms for sensitivity analyses of major bleeding according to timing of aspirin withdrawal

Supplementary Figure 19A. Restricted to trials with ≥3 months of DAPT before de-escalation to ticagrelor monotherapy

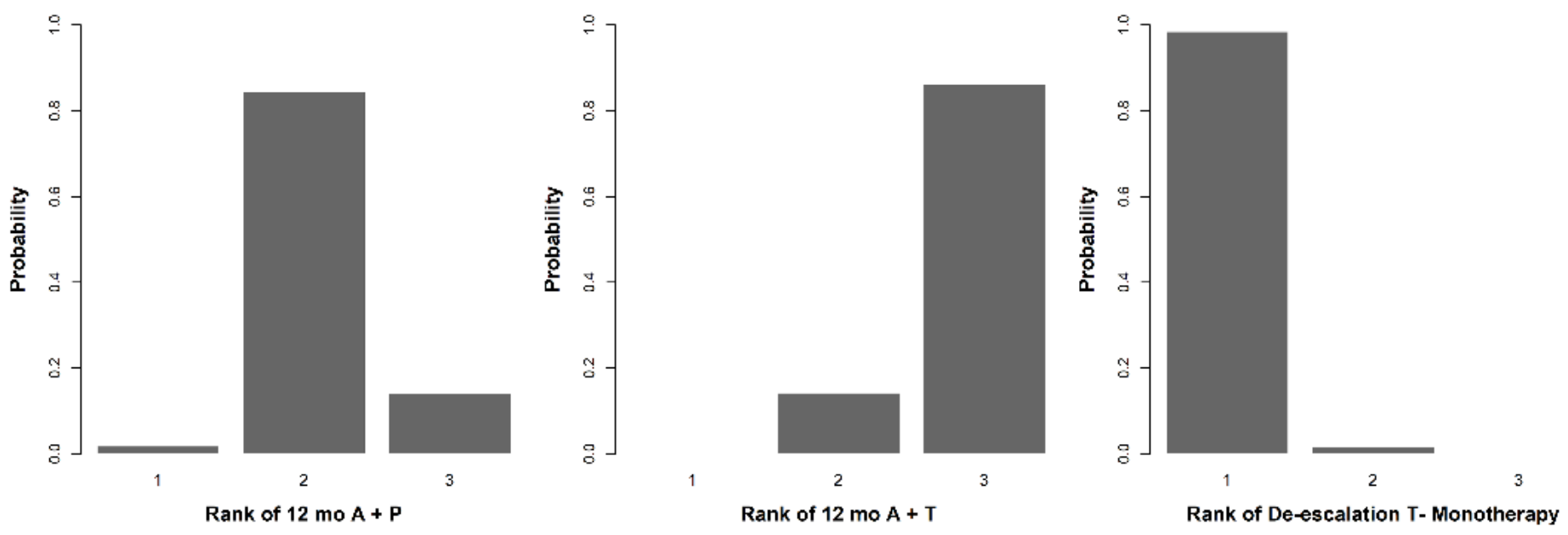

12 mo of DAPT (A + P)		
1.13 [0.89; 1.44]	12 mo of DAPT (A + T)	
0.66 [0.45; 0.97]*	0.58 [0.43; 0.79]*	1–3 mo of DAPT followed by ticagrelor monotherapy

Supplementary Figure 19B. Excluding trials with very early aspirin withdrawal (<1 month after PCI; T-PASS)

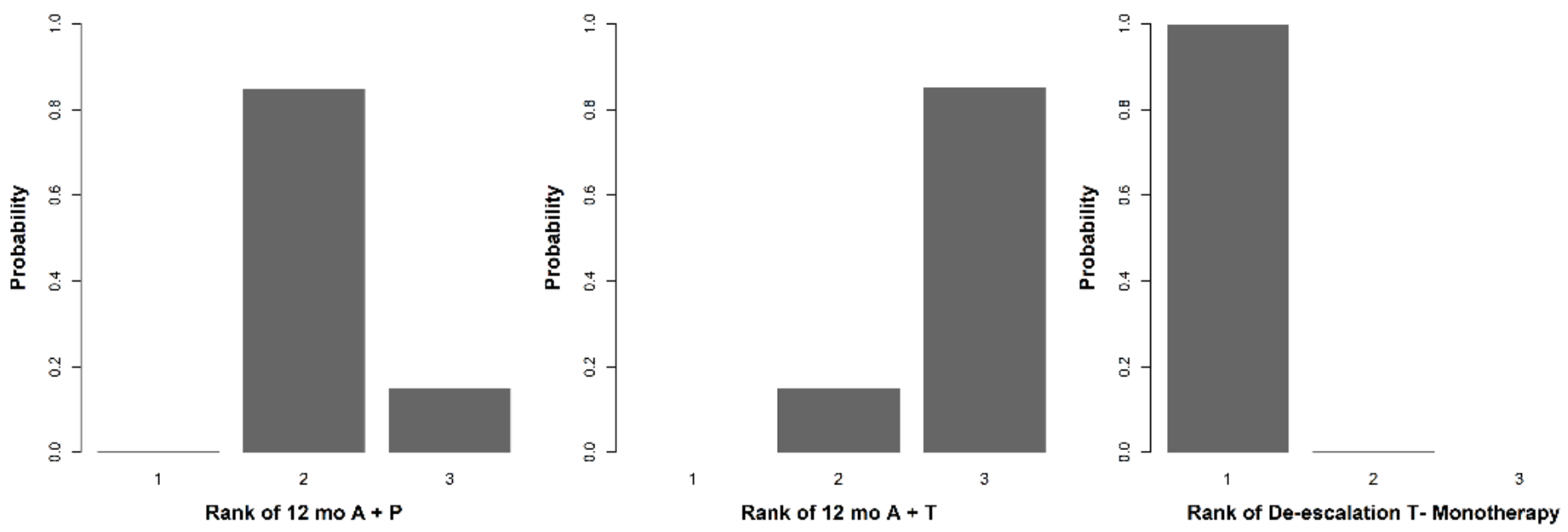

12 mo of DAPT (A + P)		
1.14 [0.87; 1.47]	12 mo of DAPT (A + T)	
0.58 [0.40; 0.83]*	0.51 [0.40; 0.65]*	1–3 mo of DAPT followed by ticagrelor monotherapy

Supplementary Figure 20. League Tables and Rankograms for the Sensitivity Analysis of Major Bleeding Using a Common-Effect Model

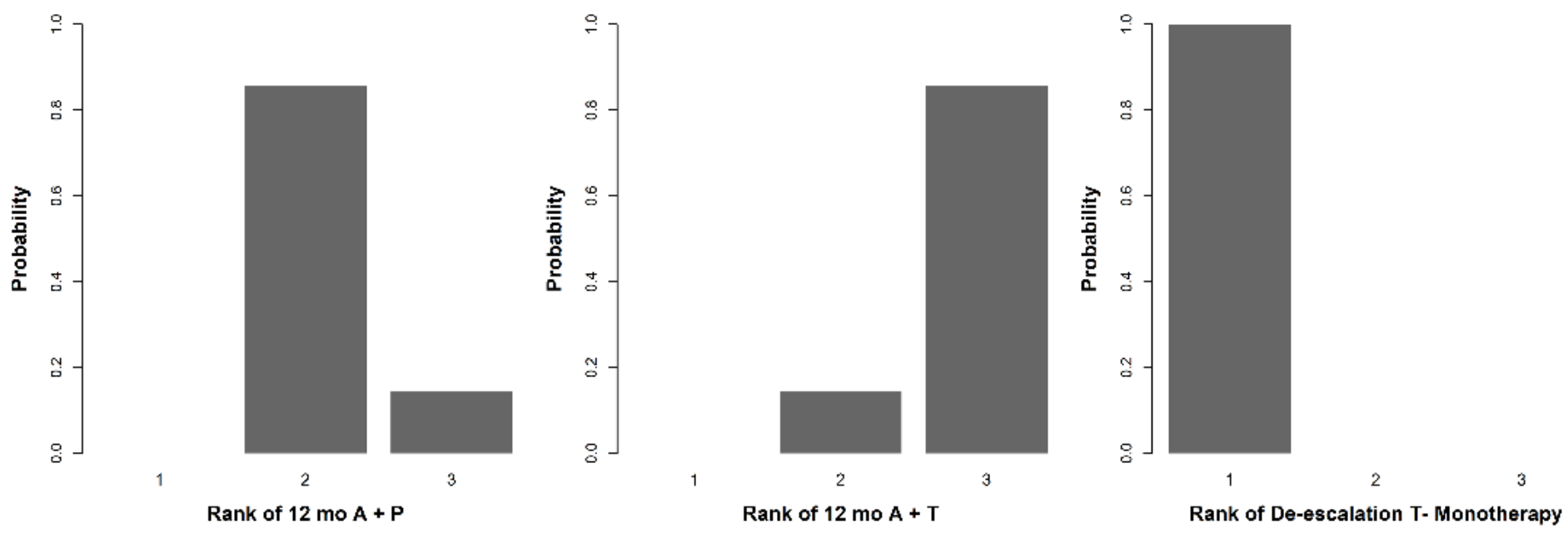

12 mo of DAPT (A + P)		
1.13 [0.89; 1.44]	12 mo of DAPT (A + T)	
0.55 [0.40; 0.76]*	0.49 [0.39; 0.61]*	1–3 mo of DAPT followed by ticagrelor monotherapy

Supplementary Figure 21. League Tables and Rankograms for the Sensitivity Analysis of Major Bleeding Using Pooled Hazard Ratios (Time-to-Event Analysis)

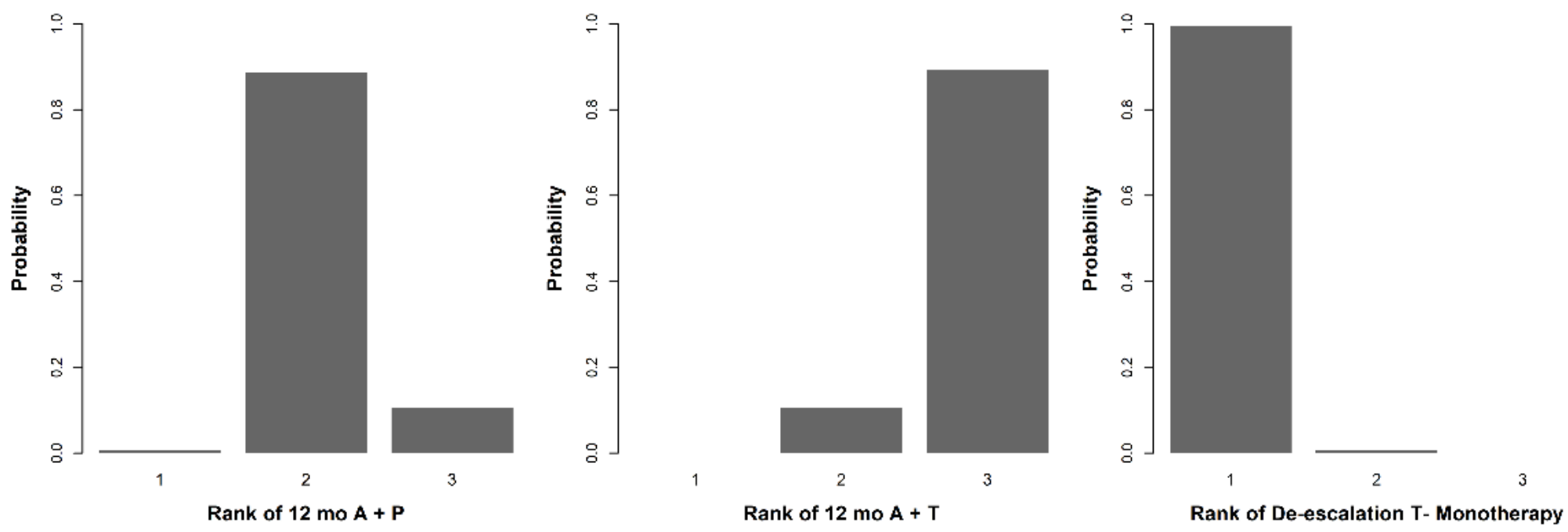

12 mo of DAPT (A + P)		
1.19 [0.89; 1.59]	12 mo of DAPT (A + T)	
0.57 [0.39; 0.83]*	0.48 [0.38; 0.61]*	1–3 mo of DAPT followed by ticagrelor monotherapy

Supplementary Figure 22. Forest plot and Rankogram for the Leave-One-Out Sensitivity Analysis of MACCE Excluding ISAR-REACT 5

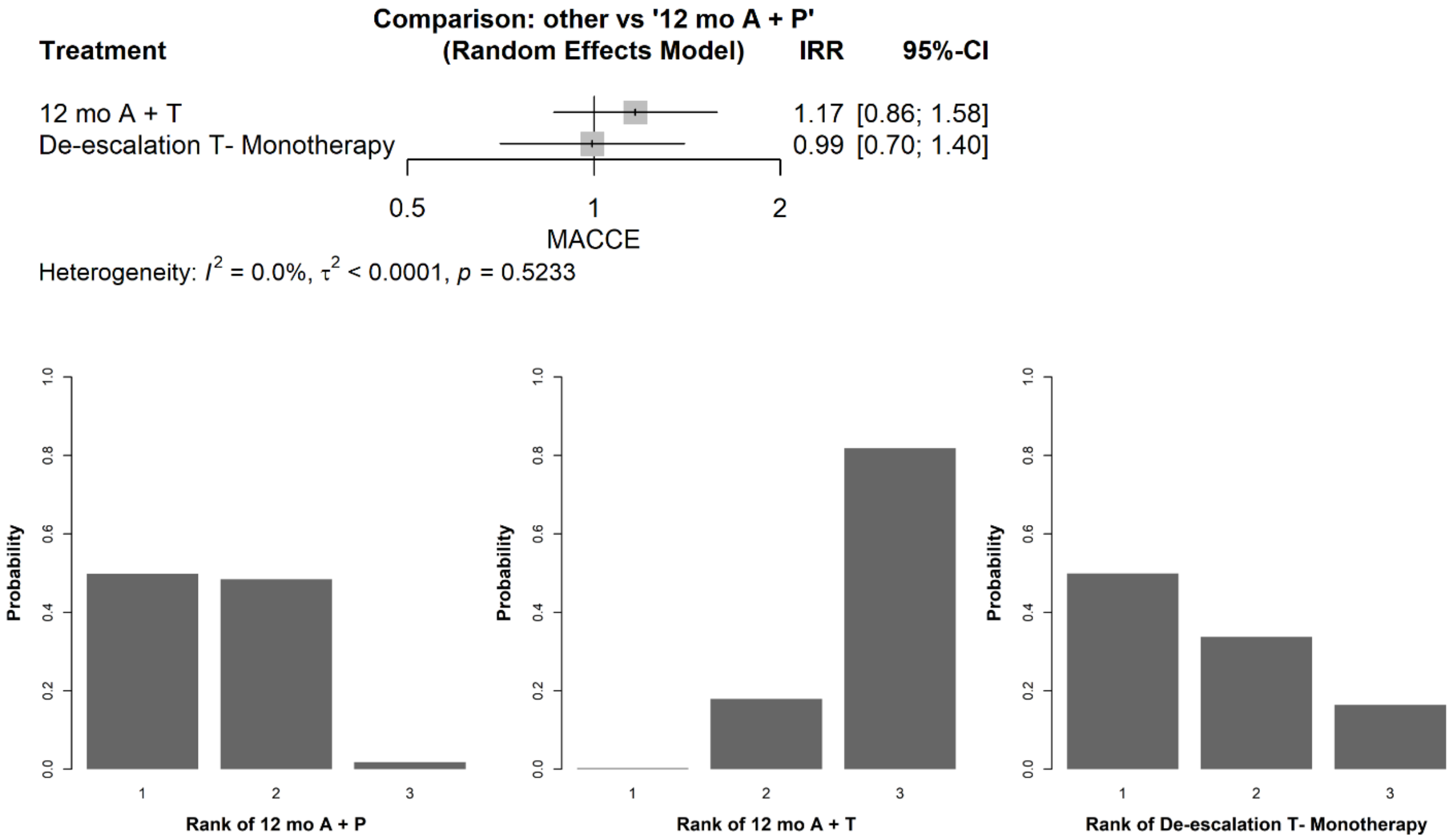

Supplementary Figure 23. Forest plot and Rankogram for the Leave-One-Out Sensitivity Analysis of MACCE Excluding TUXEDO-2

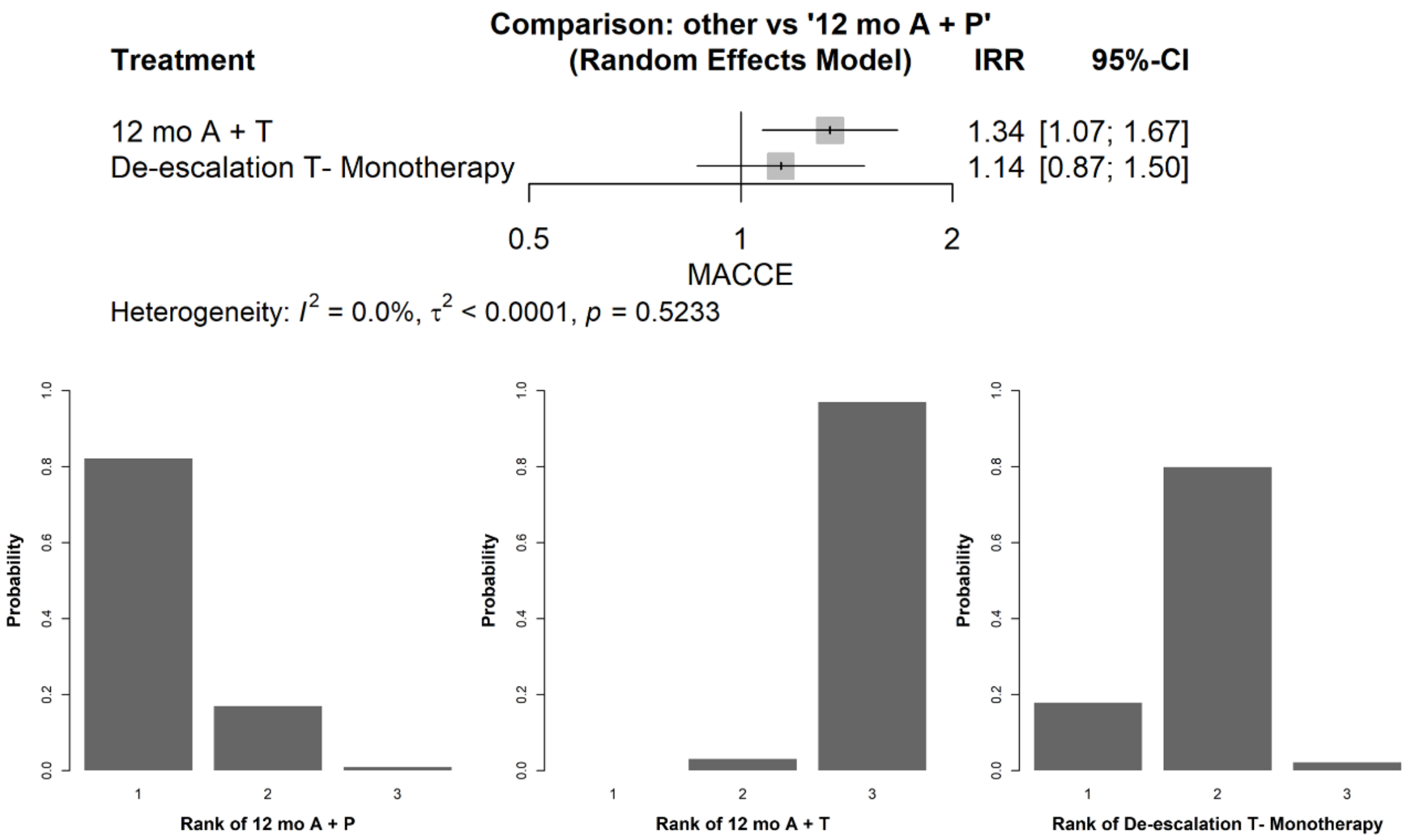

Supplementary Table 5. Meta-regression Analyses

Pairwise meta-regression for MACCE: de-escalation to ticagrelor monotherapy vs 12-month aspirin plus ticagrelor (k = 5).

Moderator	β	95% CI	p-value	I^2 residual	R^2
ACS (%)	—	—	—	—	—
Female (%)	0.0511	−0.0095 ; 0.1117	0.099	0%	0%
Diabetes (%)	0.0189	−0.0119 ; 0.0498	0.229	0%	0%
East-Asian (%)	−0.0004	−0.0048 ; 0.0039	0.848	14.8%	0%
Multivessel disease (%)	−0.0050	−0.0232 ; 0.0131	0.5875	24.07%	0.00%

Bayesian network meta-regression analyses for MACCE according to study-level characteristics.

Moderator	B (mean)	95% CrI	τ (sd.d mean)	DIC
ACS (%)	0.086	-0.276 to 0.463	0.131	24.07
Female (%)	-0.124	-0.886 to 0.570	0.124	23.85
Diabetes (%)	-0.081	-0.443 to 0.276	0.131	24.13
East-Asian (%)	-0.097	-0.560 to 0.343	0.127	23.85
Multivessel disease (%)	-0.105	-0.679 to 0.418	0.146	21.28

Treatment ranking (SUCRA values) for MACCE across Bayesian network meta-regression models including study-level moderators.

Moderator	12 mo A + P	De-escalation to T-monotherapy	12 mo A + T
ACS (%)	0.838	0.614	0.048
Female (%)	0.845	0.611	0.044
Diabetes (%)	0.840	0.615	0.045
East-Asian (%)	0.788	0.662	0.050
Multivessel disease (%)	0.845	0.576	0.079

SUCRA indicates surface under the cumulative ranking curve.
Higher SUCRA values indicate a greater probability of being the most favorable treatment for MACCE.

Pairwise meta-regression for major bleeding: de-escalation to ticagrelor monotherapy vs 12-month aspirin plus ticagrelor (k = 5).

Moderator	β	95% CI	p-value	I^2 residual	R^2
ACS (%)	—	—	—	—	—
Female (%)	−0.0062	−0.1068 ; 0.0945	0.905	44.7%	0%
Diabetes (%)	−0.0325	−0.0887 ; 0.0237	0.257	28.8%	14.8%
East-Asian (%)	0.0006	−0.0066 ; 0.0079	0.863	43.0%	0%
Multivessel disease (%)	0.0049	−0.0340 ; 0.0437	0.8060	58.36%	0.00%

Bayesian network meta-regression analyses for major bleeding according to study-level characteristics.

Moderator	B (mean)	95% CrI	τ (sd.d mean)	DIC
ACS (%)	−0.13	−0.85 to 0.58	0.27	25.55
Female (%)	0.24	−1.02 to 1.59	0.25	25.37
Diabetes (%)	0.12	−0.57 to 0.82	0.27	25.68
East-Asian (%)	0.15	−0.69 to 0.99	0.26	25.53
Multivessel disease (%)	0.160	−0.940 to 1.269	0.333	22.63

Treatment ranking (SUCRA values) for major bleeding across Bayesian network meta-regression models including study-level moderators.

Moderator	De-escalation T-monotherapy	12 mo A + P	12 mo A + T
ACS (%)	0.984	0.341	0.175
Female (%)	0.984	0.319	0.197
Diabetes (%)	0.987	0.354	0.159
East-Asian (%)	0.987	0.404	0.109
Multivessel disease (%)	0.9761	0.3247	0.1992

SUCRA indicates surface under the cumulative ranking curve.
Higher SUCRA values indicate a greater probability of being the most favorable treatment for major bleeding.

Supplementary Figure 24. Pairwise meta-analysis of the interaction between diabetes status and treatment effect on the MACCE endpoint.

A. De-escalaton to Ticagrelor monotherapy vs 12-month aspirin plus ticagrelor.

Study or Subgroup	logIRR	SE	Weight	Incidence Rate Ratio IV, Random, 95% CI
**T2DM = No**				
GLASSY	-0.1385	0.1023	30.3%	0.87 [0.71; 1.06]
TICO	-0.3540	0.2845	5.6%	0.70 [0.40; 1.23]
ULTIMATE-DAPT	-0.2604	0.2314	8.2%	0.77 [0.49; 1.21]
TWILIGHT	0.2232	0.1711	13.9%	1.25 [0.89; 1.75]
**Total (95% CI)**			**58.0%**	**0.91 [0.73; 1.15]**
Heterogeneity: $Tau^2 = 0.0219$; $Chi^2 = 4.98$, df = 3 (P = 0.1736); $I^2 = 39.7\%$				
**T2DM = Yes**				
GLASSY	-0.2211	0.1400	19.3%	0.80 [0.61; 1.05]
TICO	-0.4079	0.3450	3.9%	0.67 [0.34; 1.31]
ULTIMATE-DAPT	0.3272	0.2928	5.3%	1.39 [0.78; 2.46]
TWILIGHT	-0.2486	0.1740	13.5%	0.78 [0.55; 1.10]
**Total (95% CI)**			**42.0%**	**0.83 [0.69; 1.01]**
Heterogeneity: $Tau^2 = < 0.0001$; $Chi^2 = 3.68$, df = 3 (P = 0.2985); $I^2 = 18.4\%$				
**Total (95% CI)**			**100.0%**	**0.88 [0.77; 1.01]**
Heterogeneity: $Tau^2 = 0.0055$; $Chi^2 = 9.18$, df = 7 (P = 0.2401); $I^2 = 23.7\%$				
Test for subgroup differences: $Chi^2 = 0.35$, df = 1 (P = 0.5516)				

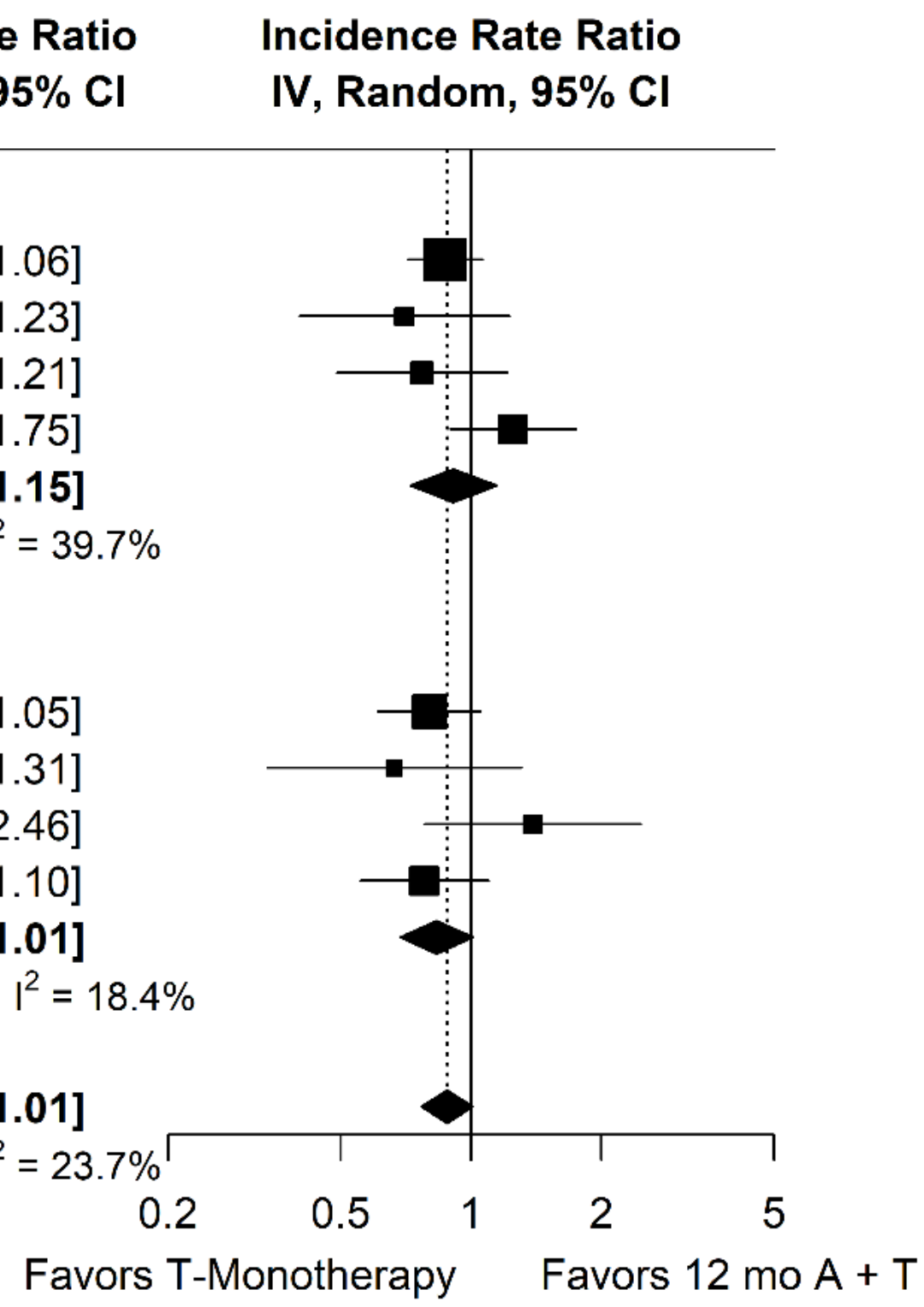

B. 12-month aspirin plus ticagrelor vs 12-month aspirin plus prasugrel.

Study or Subgroup	logIRR	SE	Weight	Incidence Rate Ratio IV, Random, 95% CI
**T2DM = No**				
ISAR-REACT 5	0.5063	0.1411	35.6%	1.66 [1.26; 2.19]
**T2DM = Yes**				
ISAR-REACT 5	-0.1518	0.1944	30.2%	0.86 [0.59; 1.26]
TUXEDO-2	0.1538	0.1552	34.2%	1.17 [0.86; 1.58]
**Total (95% CI)**			**64.4%**	**1.02 [0.76; 1.38]**
Heterogeneity: $Tau^2 = 0.0157$; $Chi^2 = 1.51$, df = 1 (P = 0.2193); $I^2 = 33.7\%$				
**Total (95% CI)**			**100.0%**	**1.21 [0.83; 1.74]**
Heterogeneity: $Tau^2 = 0.0794$; $Chi^2 = 7.93$, df = 2 (P = 0.0190); $I^2 = 74.8\%$				
Test for subgroup differences: $Chi^2 = 5.45$, df = 1 (P = 0.0195)				

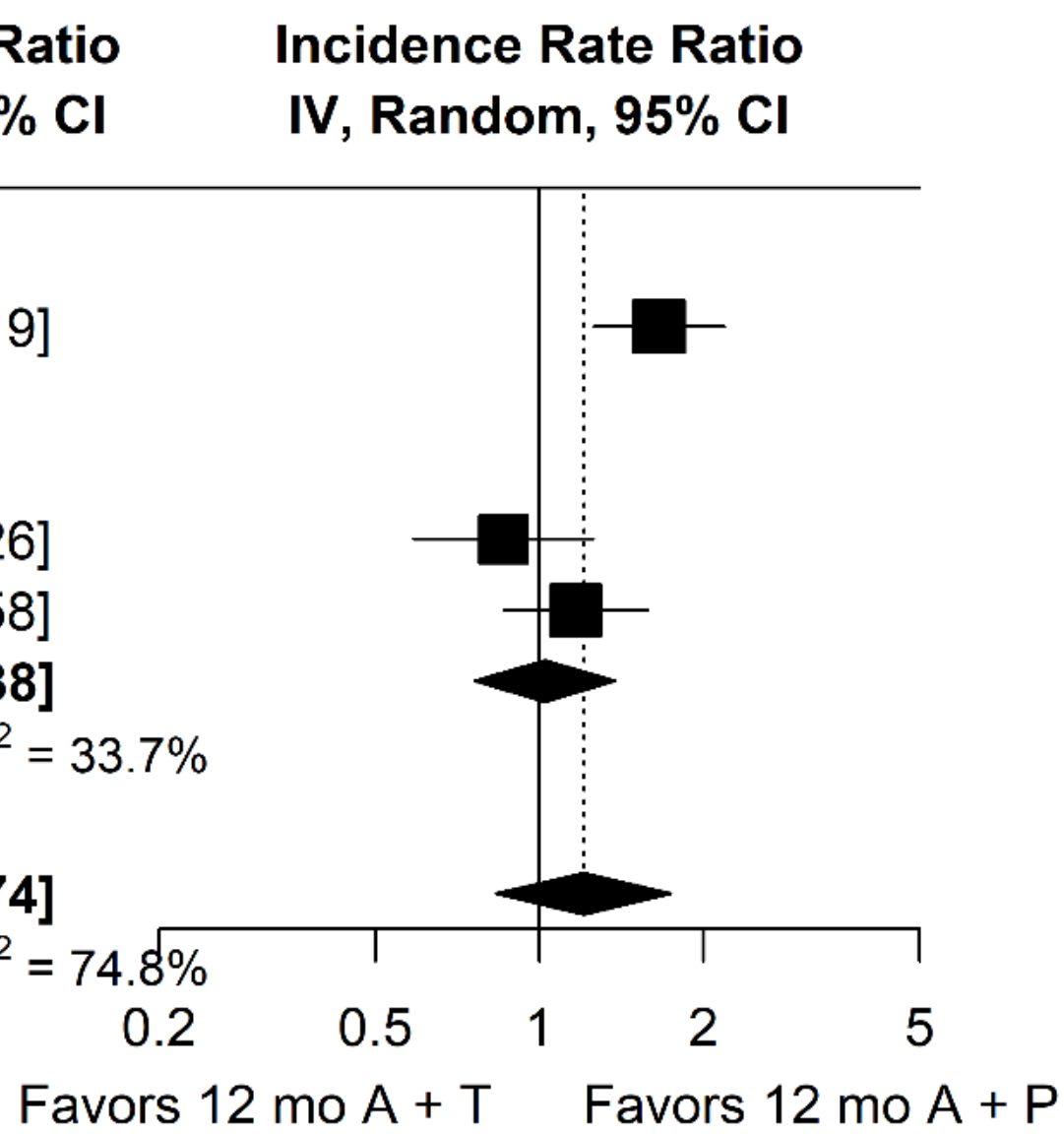

Supplementary Figure 25. Network meta-analysis forest plots, league tables, and ranking probabilities (rankograms) for MACCE in subgroup analyses of patients with diabetes

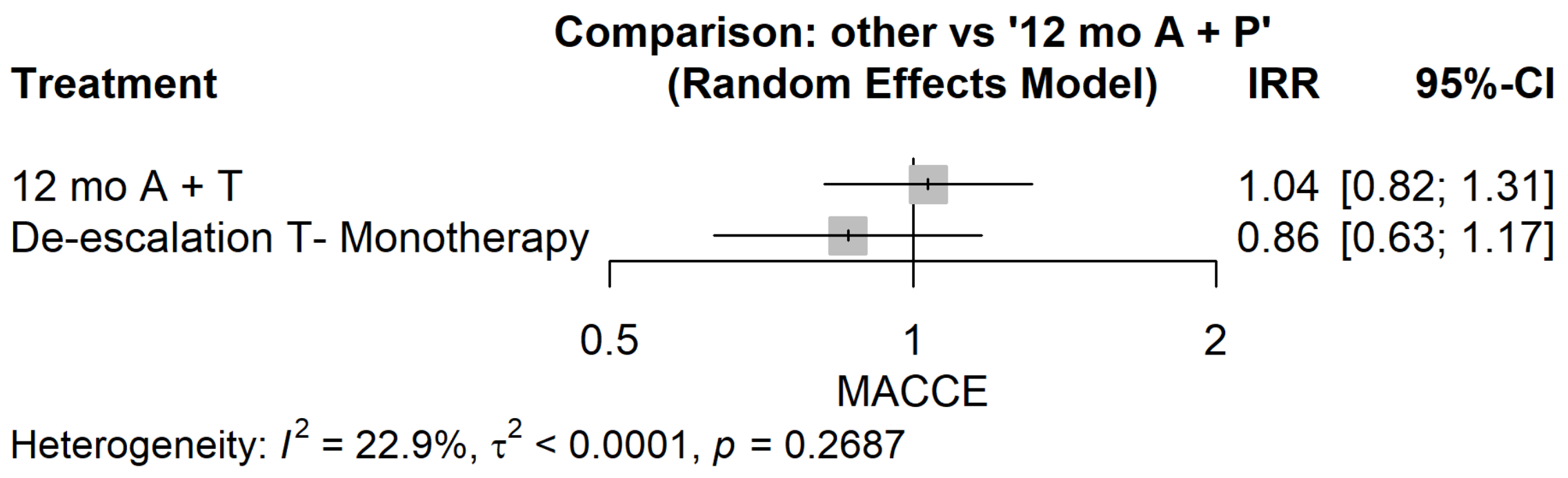

12 mo of DAPT (A + P)		
1.04 [0.82; 1.31]	12 mo of DAPT (A + T)	
0.86 [0.63; 1.17]	0.83 [0.69; 1.01]	1–3 mo of DAPT followed by ticagrelor monotherapy

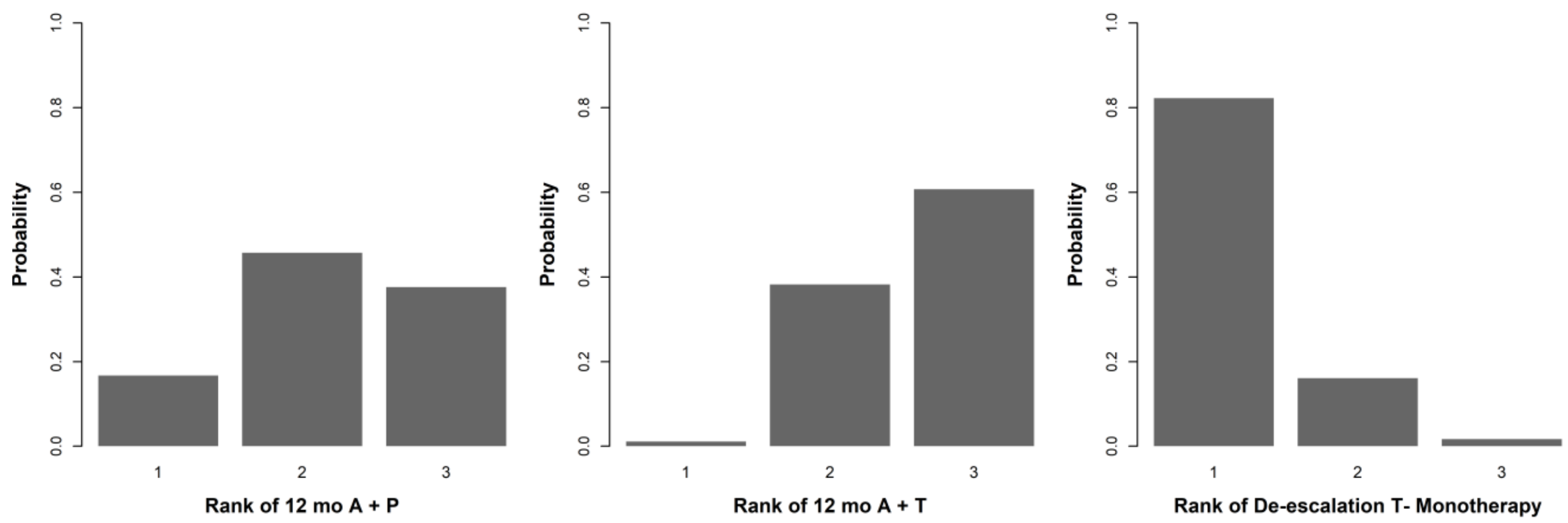

Supplementary Figure 26. Network meta-analysis forest plots, league tables, and ranking probabilities (rankograms) for MACCE in subgroup analyses of patients without diabetes

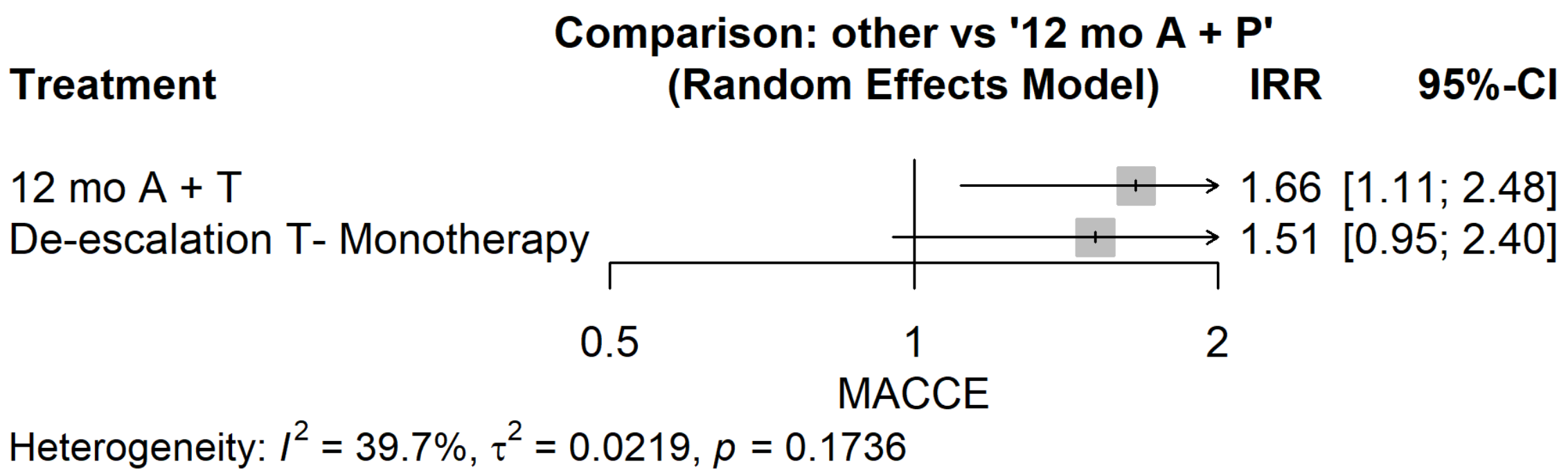

12 mo of DAPT (A + P)		
1.66 [1.11; 2.48]	12 mo of DAPT (A + T)	
1.51 [0.95; 2.40]	0.91 [0.73; 1.15]	1–3 mo of DAPT followed by ticagrelor monotherapy

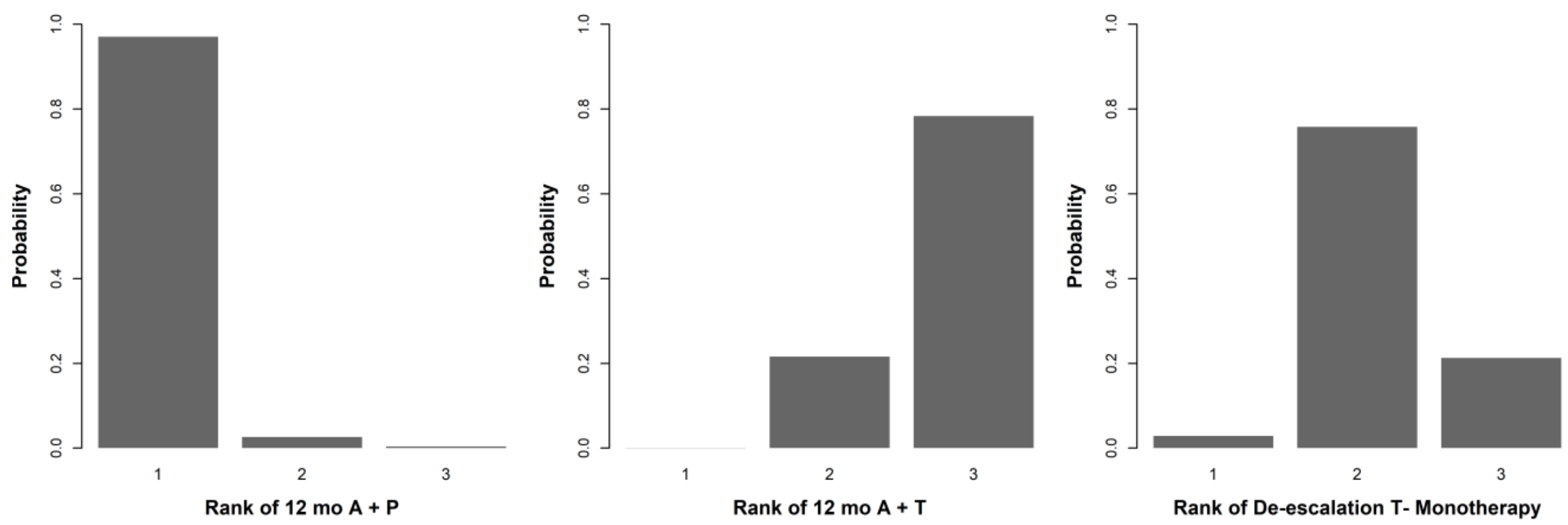

Supplementary Table 6. P-scores for Each Antithrombotic Strategy stratified by Diabetes status

**Antithrombotic Strategy**	**MACCE**		**Major bleeding**	
	Diabetes	No Diabetes	Diabetes	No Diabetes
12 mo DAPT (aspirin plus prasugrel)	0.3916	**0.9770**	0.5507	0.3352
1–3 mo of DAPT followed by ticagrelor monotherapy	**0.8996**	0.4131	**0.9029**	**0.9236**
12 mo DAPT (aspirin plus ticagrelor)	0.2089	0.1100	0.0465	0.2412

Supplementary Figure 27 Pairwise meta-analysis of the interaction between diabetes status and treatment effect on major bleeding endpoint.

A. . De-escalaton to Ticagrelor monotherapy vs 12-month aspirin plus ticagrelor.

Study or Subgroup	logIRR	SE	Weight	Incidence Rate Ratio IV, Random, 95% CI
**T2DM = No**				
GLASSY	0.0514	0.1709	16.7%	1.05 [0.75; 1.47]
TICO	-0.7282	0.3376	8.7%	0.48 [0.25; 0.94]
ULTIMATE-DAPT	-0.7879	0.2460	12.5%	0.45 [0.28; 0.74]
TWILIGHT	-0.6690	0.1347	19.0%	0.51 [0.39; 0.67]
**Total (95% CI)**			**56.8%**	**0.60 [0.40; 0.91]**
Heterogeneity: $Tau^2 = 0.1289$; $Chi^2 = 13.74$, df = 3 (P = 0.0033); $I^2 = 78.2\%$				
**T2DM = Yes**				
GLASSY	-0.1441	0.2805	10.9%	0.87 [0.50; 1.50]
TICO	-0.4079	0.3727	7.6%	0.67 [0.32; 1.38]
ULTIMATE-DAPT	-0.8303	0.3618	7.9%	0.44 [0.21; 0.89]
TWILIGHT	-0.4076	0.1699	16.8%	0.67 [0.48; 0.93]
**Total (95% CI)**			**43.2%**	**0.67 [0.52; 0.85]**
Heterogeneity: $Tau^2 = 0$; $Chi^2 = 2.25$, df = 3 (P = 0.5228); $I^2 = 0\%$				
**Total (95% CI)**			**100.0%**	**0.63 [0.49; 0.80]**
Heterogeneity: $Tau^2 = 0.0627$; $Chi^2 = 16.19$, df = 7 (P = 0.0235); $I^2 = 56.8\%$				
Test for subgroup differences: $Chi^2 = 0.16$, df = 1 (P = 0.6854)				

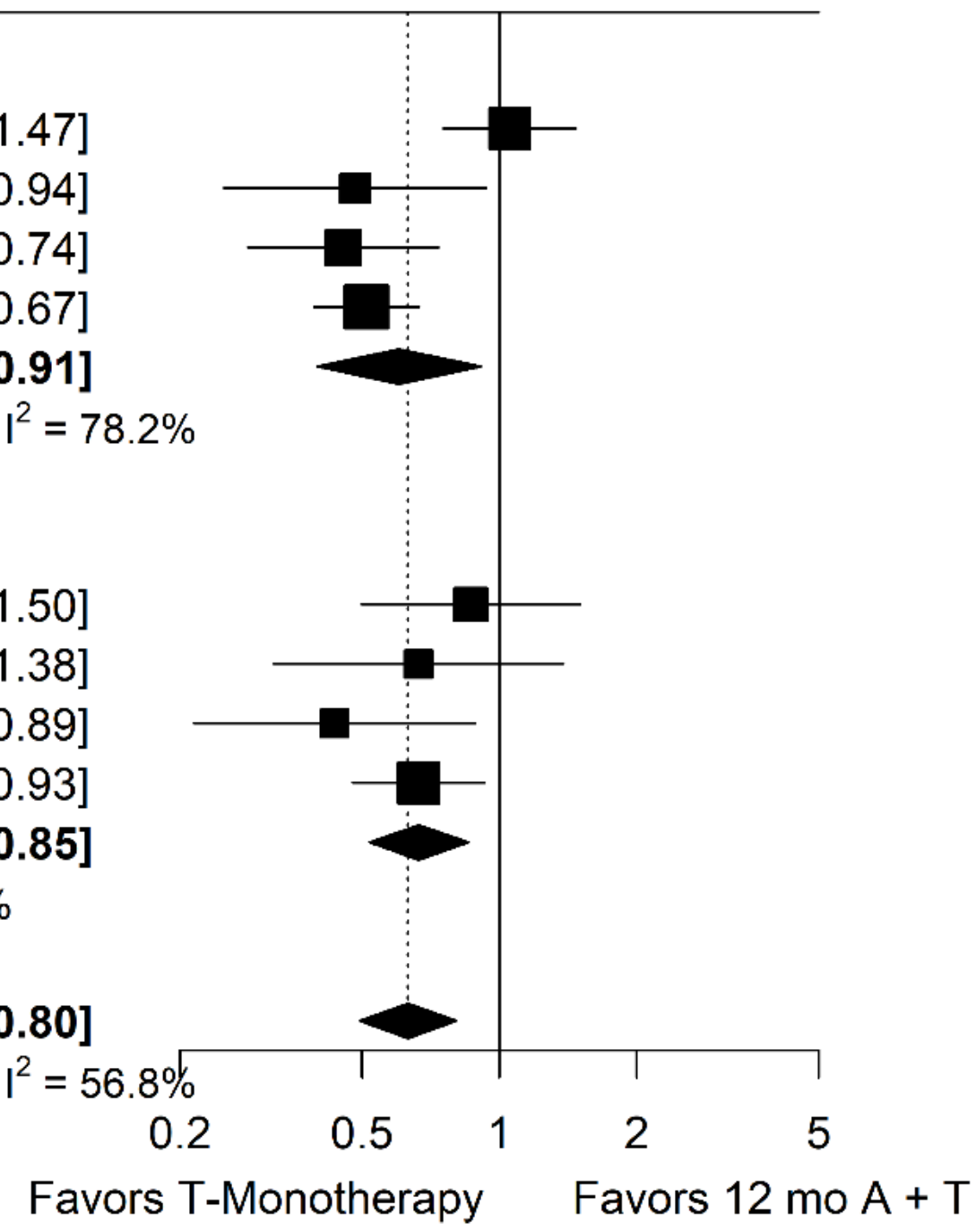

B. 12-month aspirin plus ticagrelor vs 12-month aspirin plus prasugrel.

Study or Subgroup	logIRR	SE	Weight	Incidence Rate Ratio IV, Random, 95% CI
**T2DM = No**				
ISAR-REACT-5	0.0259	0.1771	47.5%	1.03 [0.73; 1.45]
**T2DM = Yes**				
ISAR-REACT-5	0.1791	0.2994	16.6%	1.20 [0.67; 2.15]
TUXEDO-2	0.2439	0.2036	35.9%	1.28 [0.86; 1.90]
**Total (95% CI)**			**52.5%**	**1.25 [0.90; 1.74]**
Heterogeneity: $Tau^2 = 0$; $Chi^2 = 0.03$, df = 1 (P = 0.8580); $I^2 = 0\%$				
**Total (95% CI)**			**100.0%**	**1.14 [0.90; 1.45]**
Heterogeneity: $Tau^2 = 0$; $Chi^2 = 0.69$, df = 2 (P = 0.7097); $I^2 = 0.0\%$				
Test for subgroup differences: $Chi^2 = 0.65$, df = 1 (P = 0.4188)				

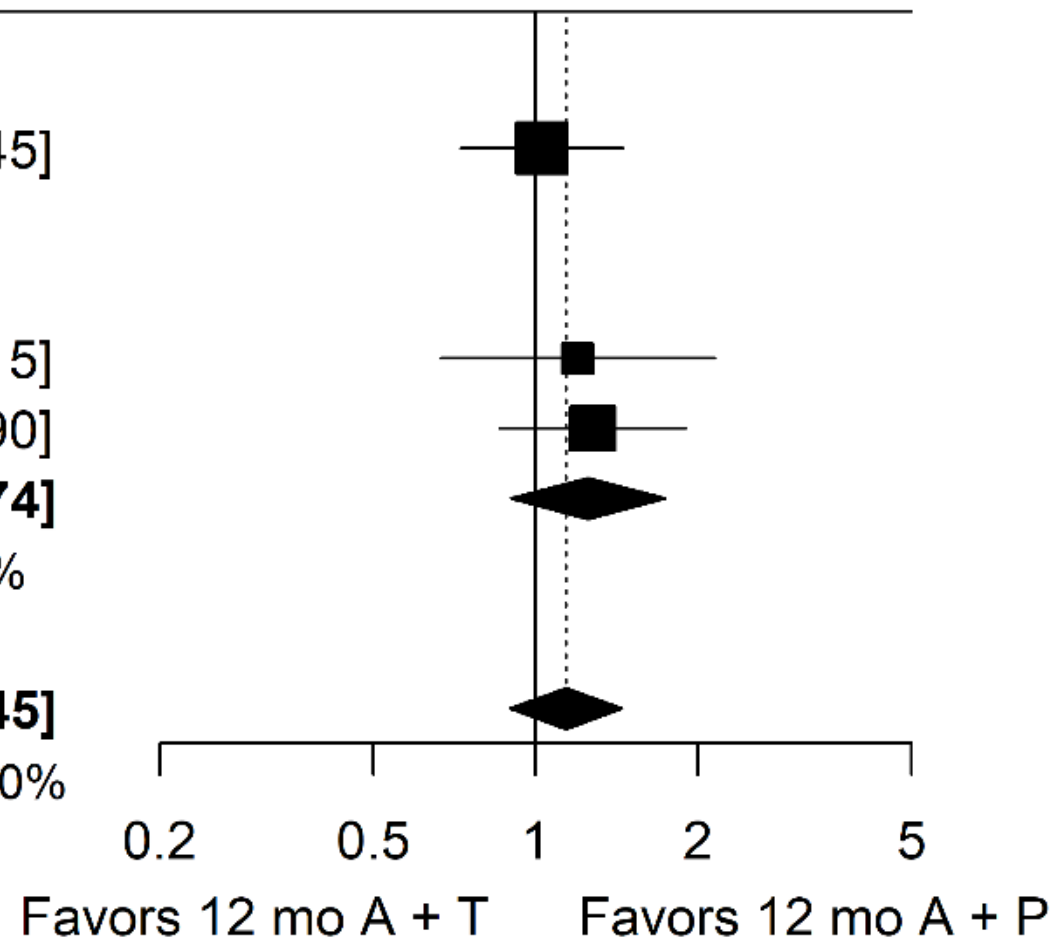

Supplementary Figure 28. Network meta-analysis forest plots, league tables, and ranking probabilities (rankograms) for the major bleeding endpoint in patients with diabetes.

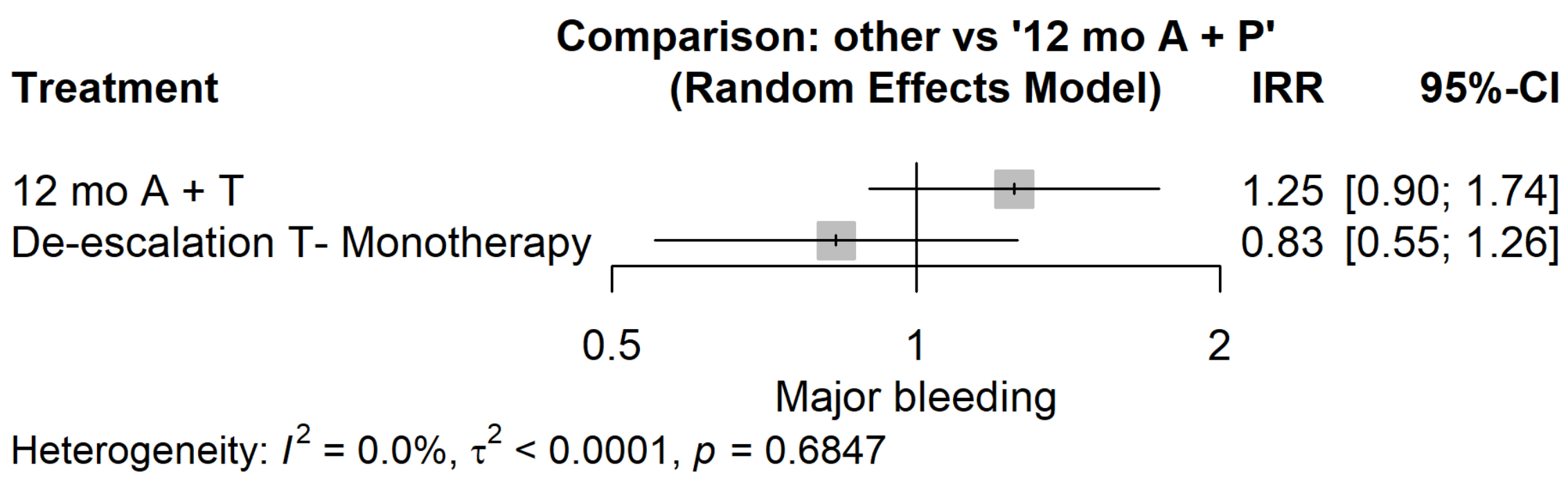

12 mo of DAPT (A + P)		
1.25 [0.90; 1.74]	12 mo of DAPT (A + T)	
0.83 [0.55; 1.26]	0.67 [0.52; 0.85]*	1–3 mo of DAPT followed by ticagrelor monotherapy

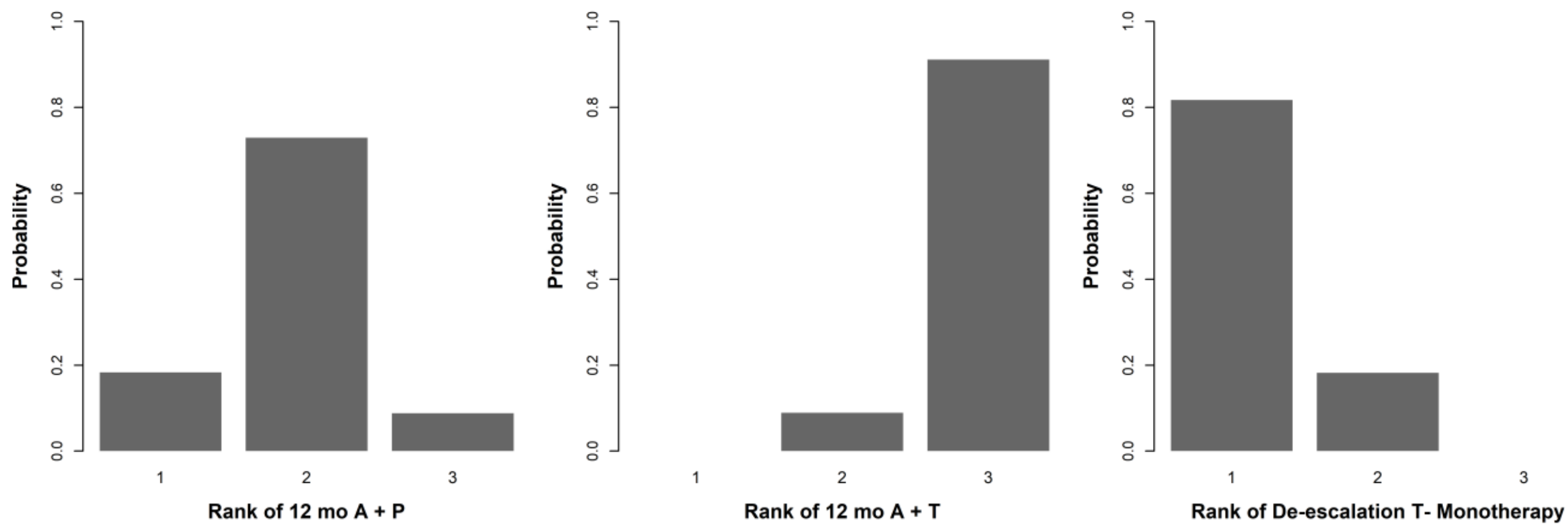

Supplementary Figure 29. Network meta-analysis forest plots, league tables, and ranking probabilities (rankograms) for the major bleeding endpoint in patients without diabetes.

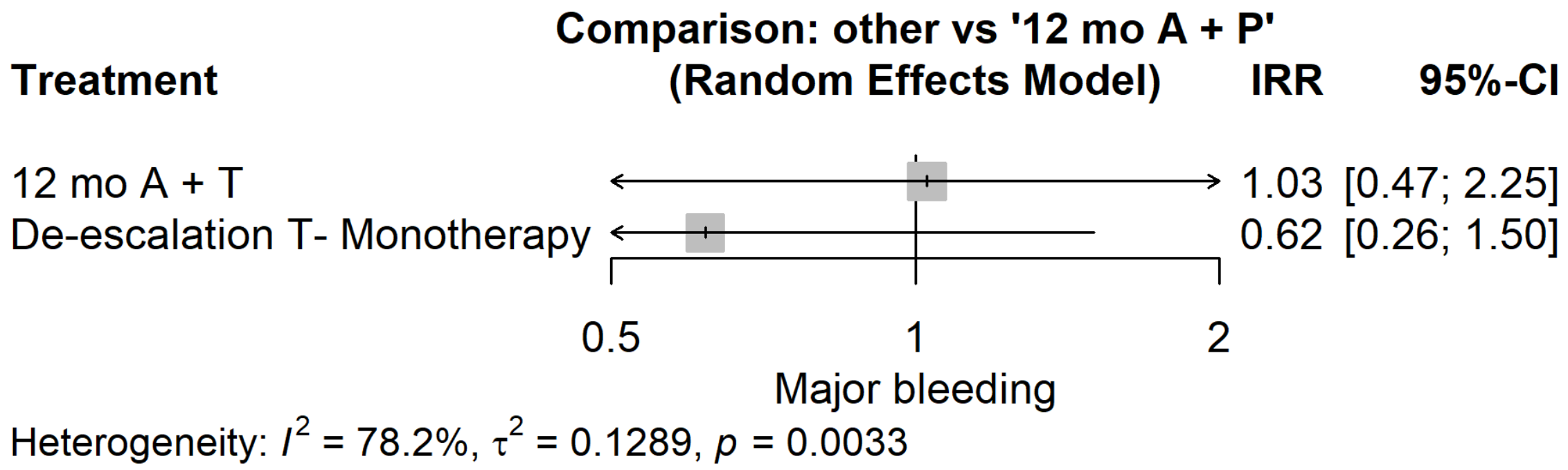

12 mo of DAPT (A + P)		
1.03 [0.47; 2.25]	12 mo of DAPT (A + T)	
0.62 [0.26; 1.50]	0.60 [0.40; 0.91] *	1–3 mo of DAPT followed by ticagrelor monotherapy

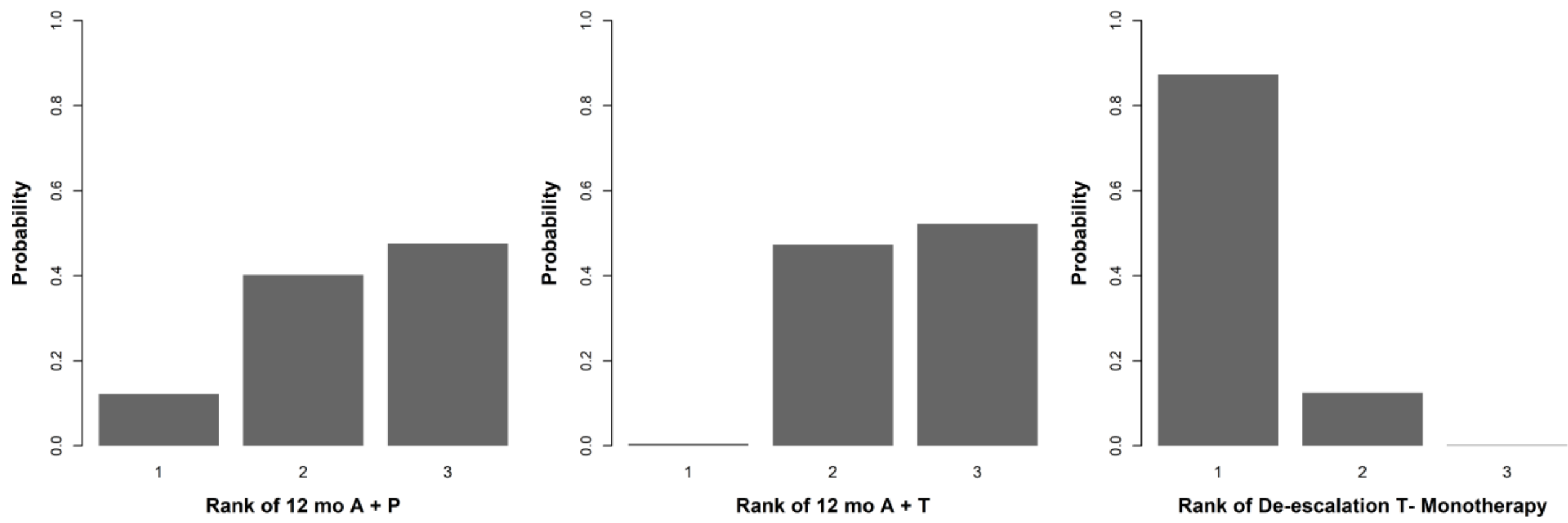

Supplementary Figure 30. Sensitivity analysis for MACCE restricted to ACS patients with diabetes, excluding GLASSY, TWILIGHT, and TUXEDO-2, and including TWILIGHT-Diabetes-ACS.

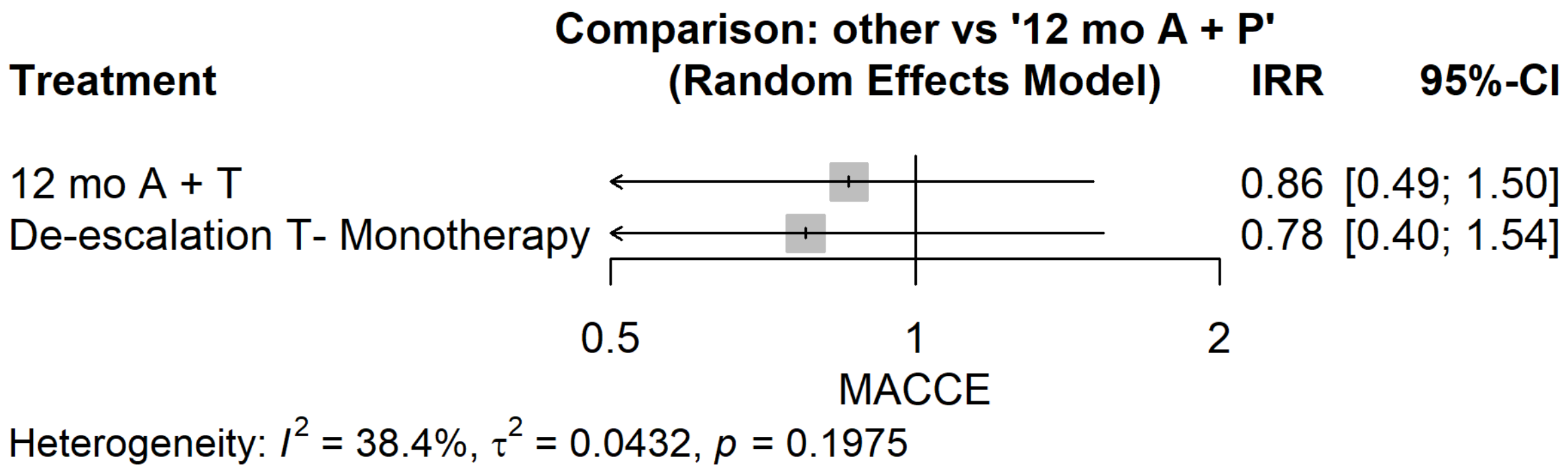

12 mo of DAPT (A + P)		
0.86 [0.49; 1.50]	12 mo of DAPT (A + T)	
0.78 [0.40; 1.54]	0.91 [0.62; 1.34]	1–3 mo of DAPT followed by ticagrelor monotherapy

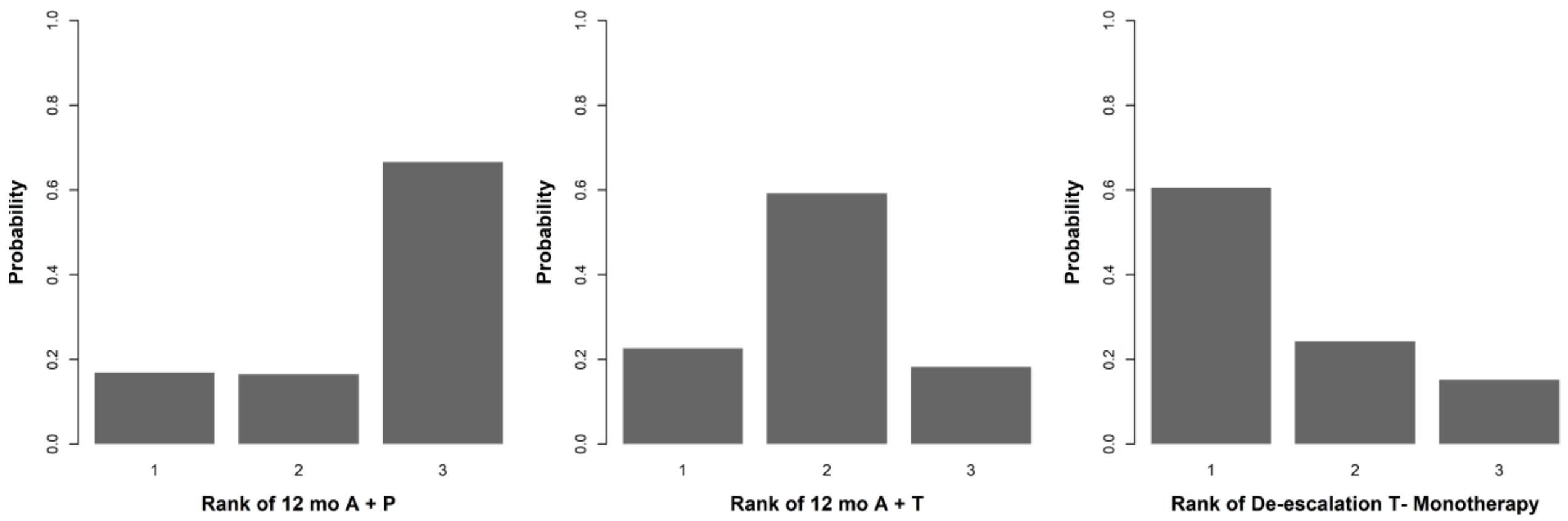

Supplementary Figure 31. Sensitivity analysis for major bleeding restricted to ACS patients with diabetes, excluding GLASSY, TWILIGHT, and TUXEDO-2, and including TWILIGHT-T2DM-ACS.

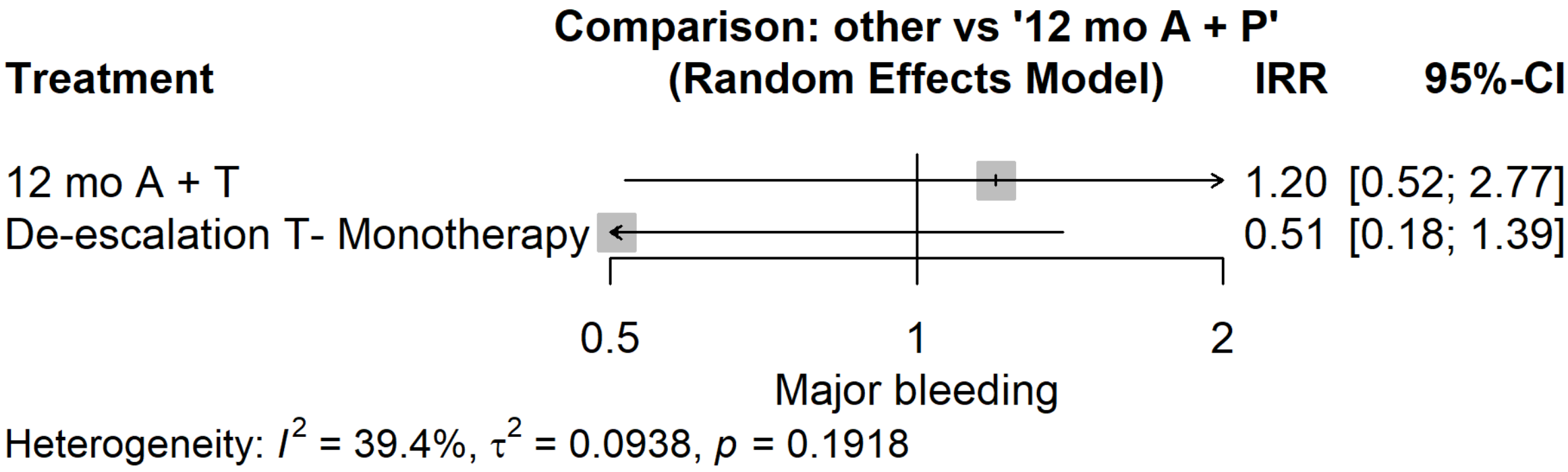

12 mo of DAPT (A + P)		
1.20 [0.52; 2.77]	12 mo of DAPT (A + T)	
0.51 [0.18; 1.39]	0.42 [0.24; 0.74]	1–3 mo of DAPT followed by ticagrelor monotherapy

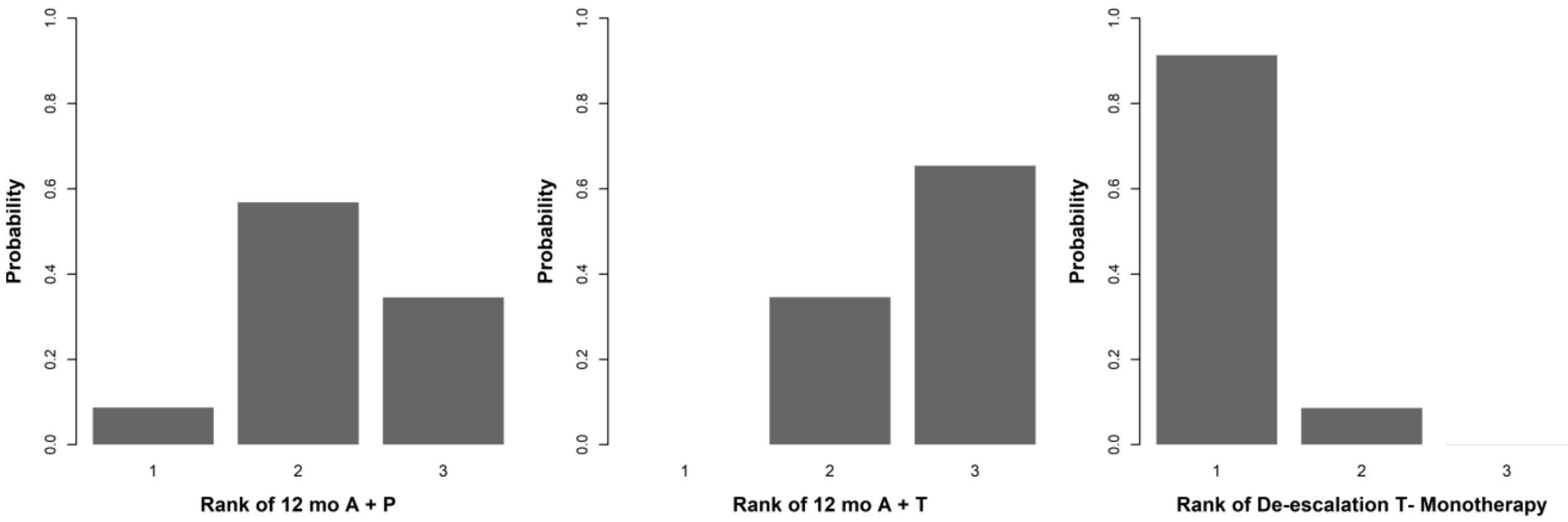

Supplementary Figure 32. Benefit–Risk Tradeoff Between MACCE and Major Bleeding in Patients With Diabetes

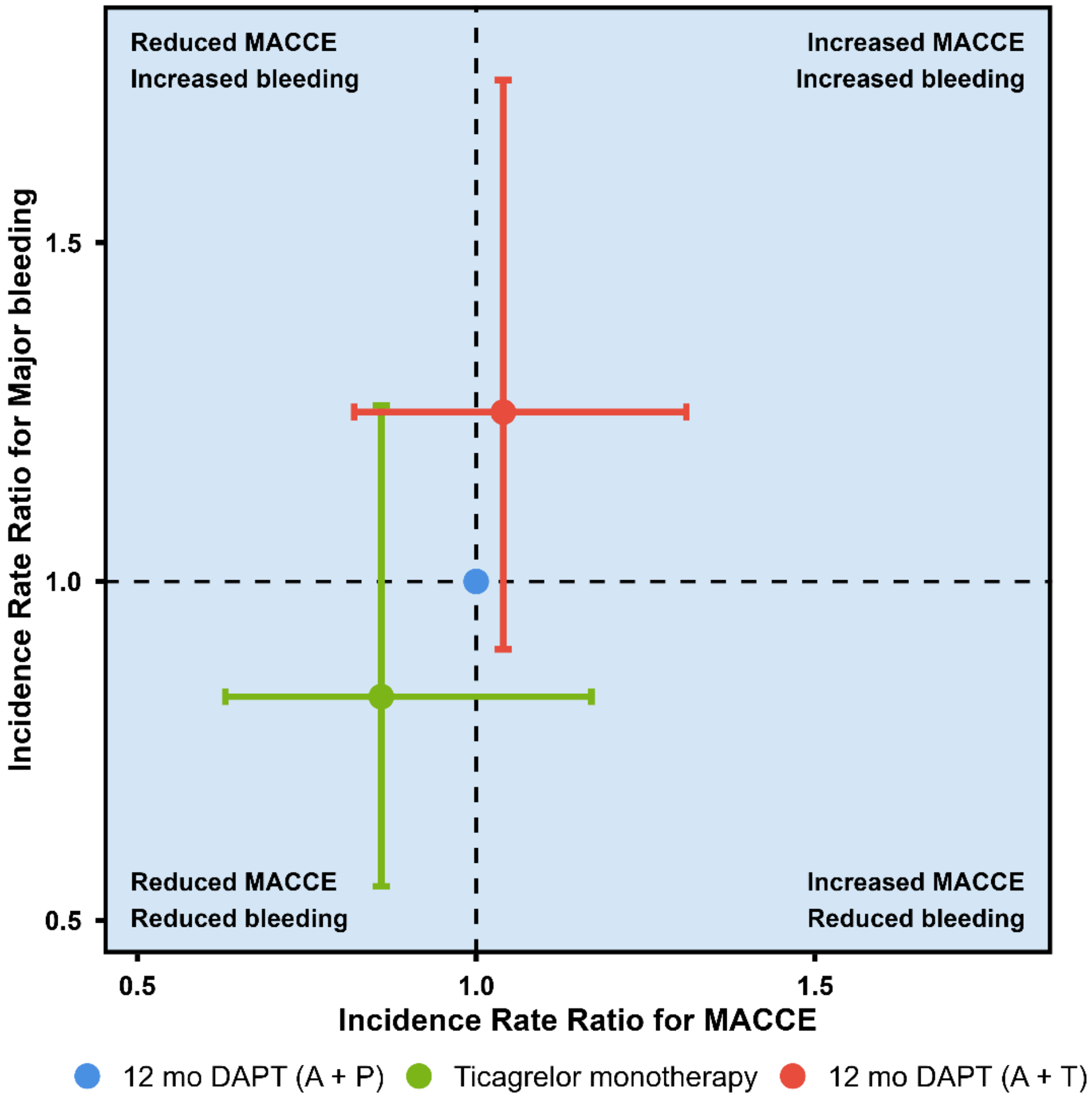

Incidence rate ratios for MACCE (x-axis) and major bleeding (y-axis) across antiplatelet strategies relative to 12 months of aspirin plus prasugrel (reference), with corresponding 95% confidence intervals. Dashed lines indicate the line of no effect (IRR = 1). The plot illustrates the relationship between ischemic and bleeding risk across treatment strategies in patients with diabetes.

Supplementary Table 7. Study-Level Event Counts and Effect Estimates Stratified by Diabetes Status

Study Acronym (First Author, Year)	ACS %	FU	MACCE		Major Bleeding	
			TD2M	No-TD2M	TD2M	No-TD2M
**†GLASSY** (Franzone et al., 2019)[8]	50.5%	24 mo	I: 93/923<br>C: 113/899<br>RR 0.79 (0.60-1.05)	I: 178/2870<br>C: 206/2892<br>RR 0.87 (0.71-1.07)	I: 24/923<br>C: 27/899<br>RR 0.87 (0.50-1.50)	I: 70/2870<br>C: 67/2892<br>RR 1.06 (0.76-1.48)
**TICO** (Kim et al., 2020)[2]	100%	12 mo	I: 14/418<br>C: 21/417<br>HR 0.67 (0.34-1.31)	I: 21/1109<br>C: 30/1112<br>HR 0.70 (0.40-1.23)	I: 12/418<br>C: 18/417<br>HR 0.67 (0.32-1.39)	I: 13/1109<br>C: 27/1112<br>HR 0.48 (0.25-0.94)
**ULTIMATE-DAPT** (Ge et al., 2024)[5]	100%	12 mo	I: 28/540<br>C: 20/535<br>HR 1.43 (0.80-2.53)	I: 33/1160<br>C: 43/1165<br>HR 0.77 (0.49-1.21)	I: 11/540<br>C: 25/535<br>HR 0.43 (0.21-0.87)	I: 24/1160<br>C: 53/1165<br>HR 0.45 (0.28-0.73)
**†TWILIGHT** (Mehran et al., 2020)[9]	64.8%	12 mo	I: 59/1282<br>C: 75/1271<br>HR 0.77 (0.55-1.09)§	I: 76/2171<br>C: 62/2214<br>HR1.24 (0.89-1.73)§	I: 58/1319<br>C: 86/1301<br>HR 0.65 (0.47-0.91)	I: 83/2236<br>C: 164/2263<br>HR 0.50 (0.39-0.66)
**TWLIGHT- T2DM - ACS ‡** (Angiolillo et al., 2020)[10]	100%	12 mo	I: 43/810<br>C: 53/804<br>HR 0.80 (0.54 – 1.20)		I: 6/810<br>C: 26/804<br>HR 0.23(0.09 – 0.55)	
**T-PASS** (Hong et al., 2023)[4]	100%	12 mo	NA	NA	NA	NA
**ISAR-REACT 5 T2DM** (Ndrepepa et al., 2020)[11]	100%	12 mo	I: 51/463<br>C: 55/429<br>HR 0.84 (0.58-1.24)	I: 132/1548<br>C: 81/1576<br>HR 1.70 (1.29-2.24)	I: 27/455<br>C: 19/383<br>HR 1.27 (0.70-2.29)	I: 68/1534<br>C: 60/1389<br>HR 1.13 (0.80-1.60)
**†TUXEDO-2** (Bangalore et al., 2026)[7]	79%	12 mo	I: 90/901<br>C: 77/899<br>HR 1.17 (0.87-1.59)	NA	I: 55/901<br>C: 43/899<br>HR 1.31 (0.88-1.95)	NA

† GLASSY, TWILIGHT, and TUXEDO-2 did not exclusively enroll patients with acute coronary syndromes and were therefore excluded from the sensitivity analysis restricted to patients with ACS and diabetes.
‡ The prespecified TWILIGHT-T2DM ACS substudy, which exclusively enrolled patients with ACS and diabetes, was included in the ACS-restricted sensitivity analysis.
§ Outcomes are reported for the per-protocol cohort.

Supplementary Results 3. Quality Assessment and Risk of Bias

No evidence of publication bias was identified on visual inspection of the comparison-adjusted funnel plot (*Supplementary Figure 31*). A formal Egger test was not performed because of the limited number of included studies (k=7). Study quality was assessed using the Cochrane Risk of Bias 2 (RoB 2) tool. Two studies were judged as having some concerns in domain 2, reflecting potential bias due to deviations from intended interventions in subgroup analyses of randomized trials, whereas all remaining studies were judged at low risk of bias (*Supplementary Figure 32*).

The certainty of evidence was evaluated using the Grading of Recommendations Assessment, Development and Evaluation (GRADE) framework (*Supplementary Tables 8–10*).

For direct comparisons, the certainty of evidence was rated as high for both MACCE and major bleeding when comparing ticagrelor monotherapy with 12 months of aspirin plus ticagrelor. The comparison between 12 months of aspirin plus ticagrelor and aspirin plus prasugrel showed high certainty for MACCE and moderate certainty for major bleeding, downgraded for imprecision.

For indirect comparisons, the certainty of evidence for ticagrelor monotherapy versus 12 months of aspirin plus prasugrel was rated as moderate for both MACCE and major bleeding. For MACCE, the certainty was downgraded for imprecision of the indirect estimate. For major bleeding, the certainty was moderate because the indirect comparison was informed by one direct comparison with moderate certainty. In the network meta-analysis, the overall certainty of evidence remained high for direct comparisons and moderate for the indirect comparison between ticagrelor monotherapy and aspirin plus prasugrel, with no concerns for incoherence across the network.

Supplementary Figure 33. Publication Bias Assessment

Supplementary Figure 33A. MACCE

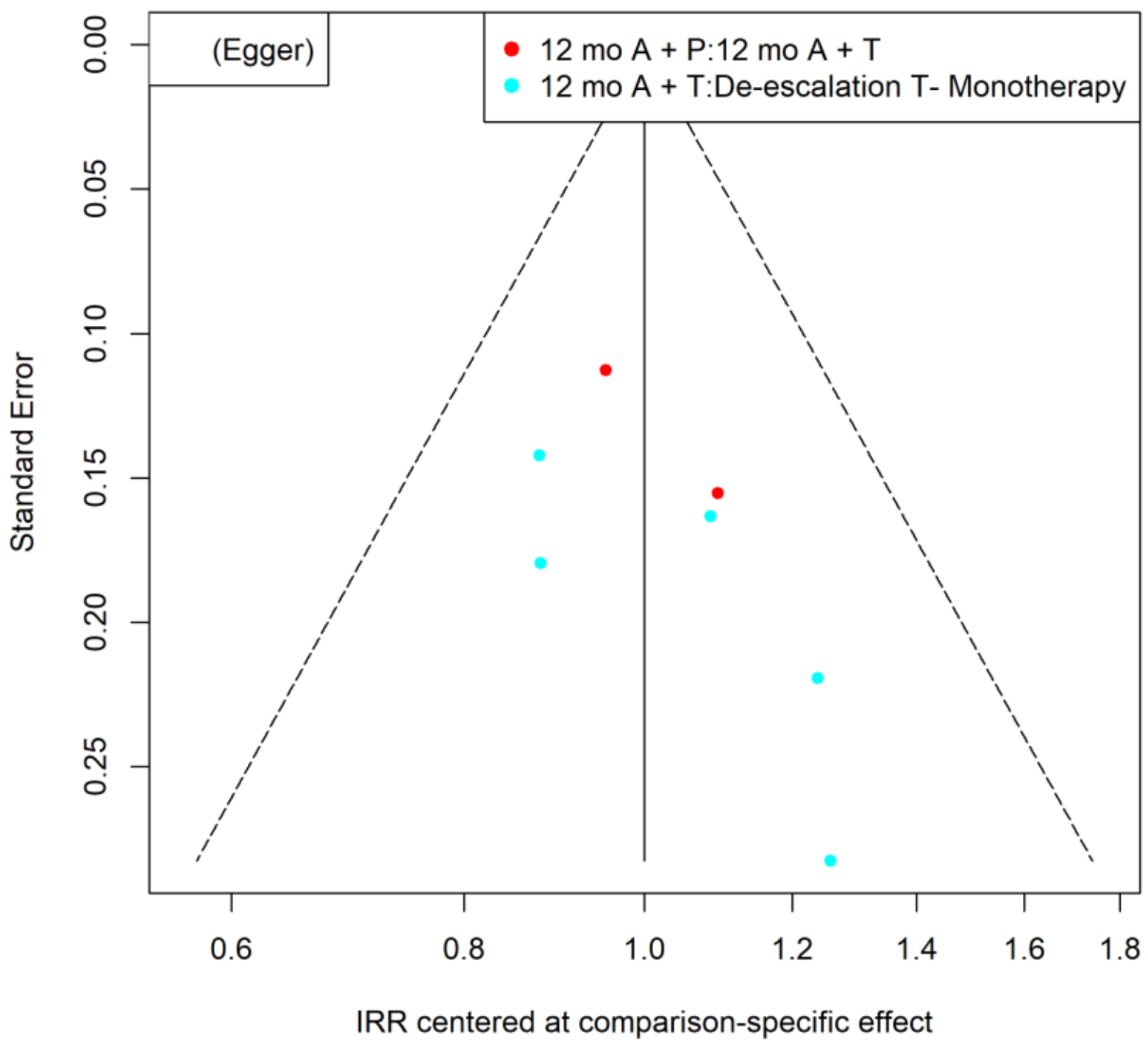

Supplementary Figure 33B. Major bleeding

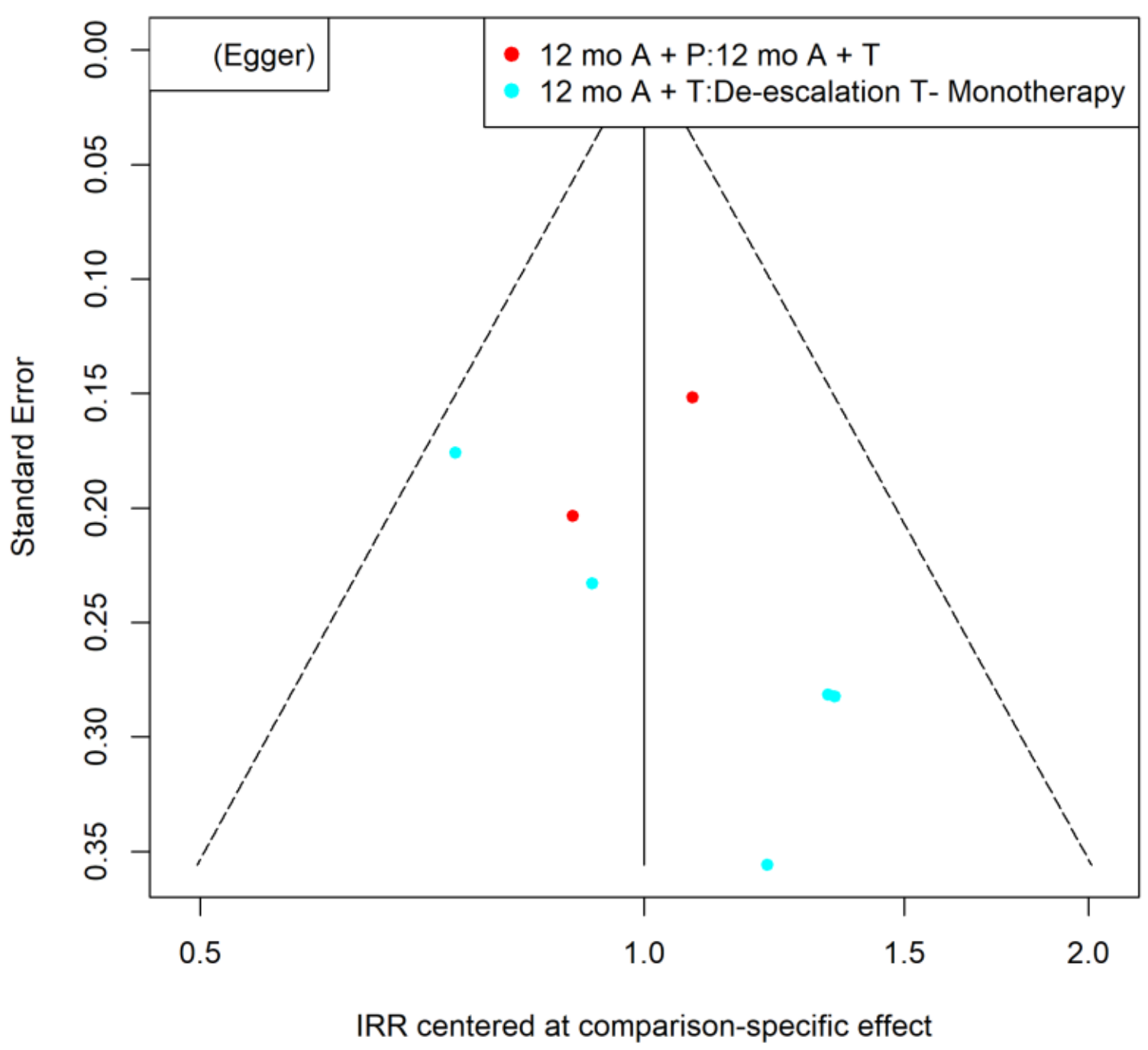

Supplementary Figure 33C. All-cause death

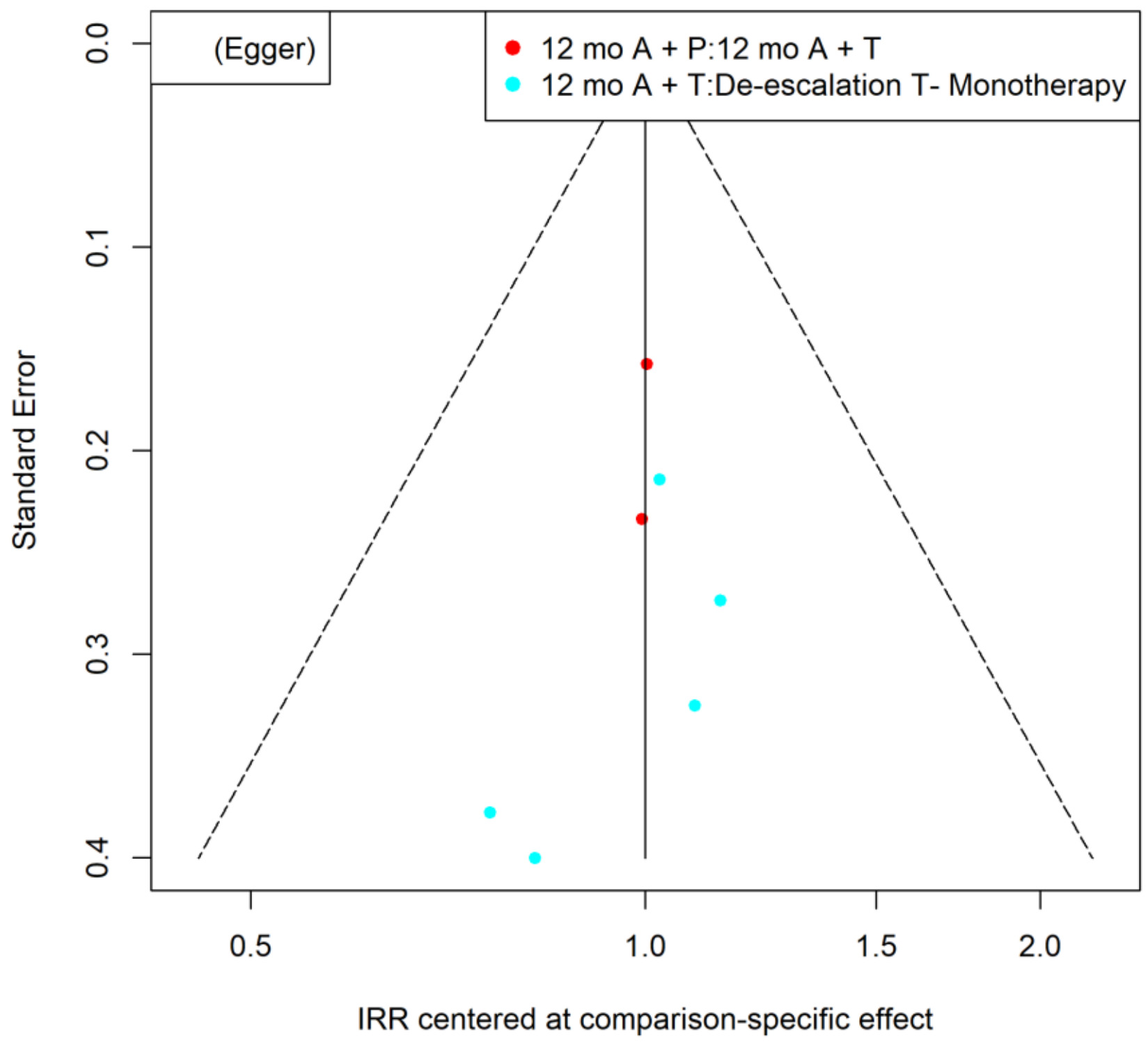

Supplementary Figure 33D. Cardiac death

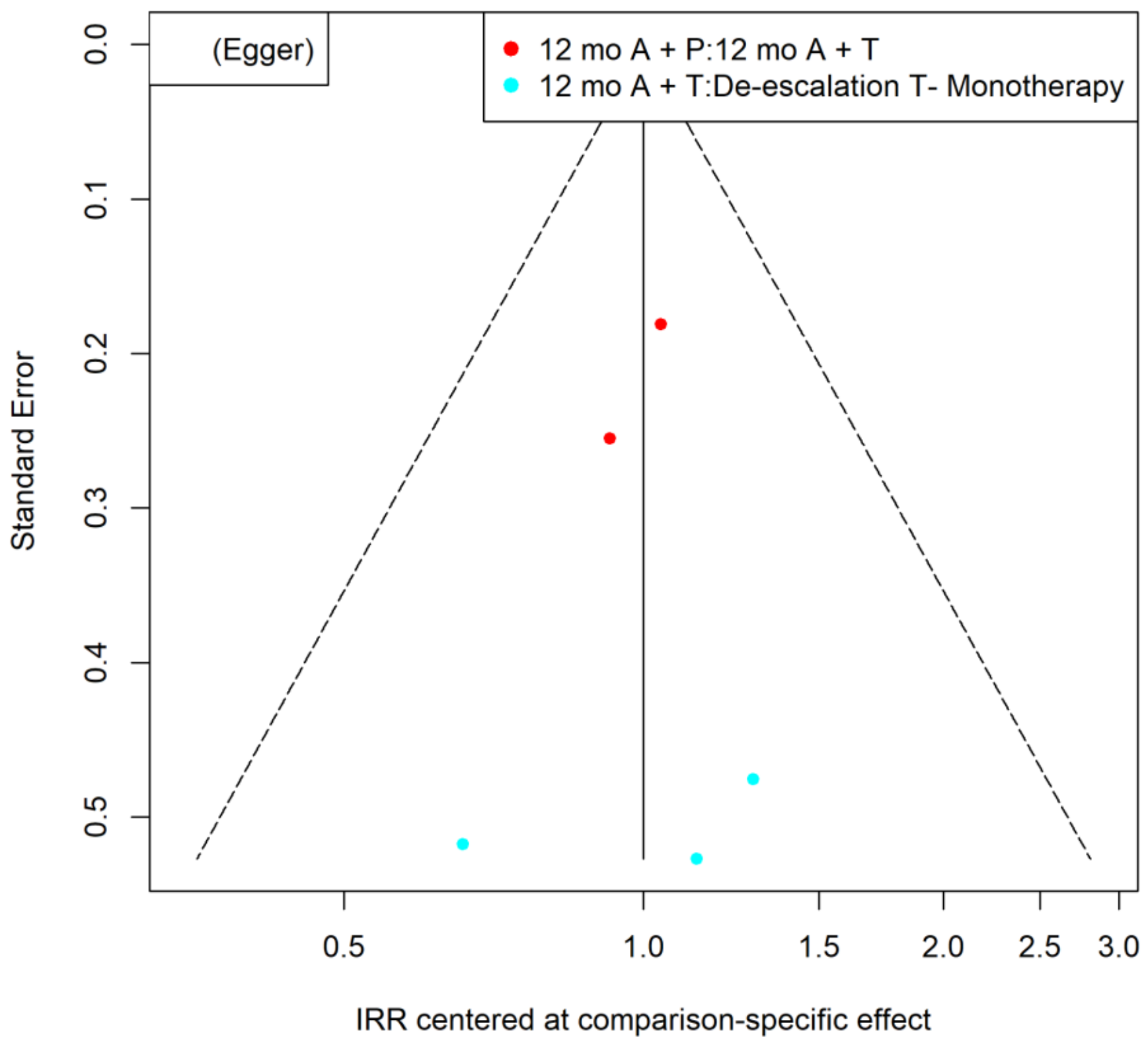

Supplementary Figure 33E. Myocardial infarction

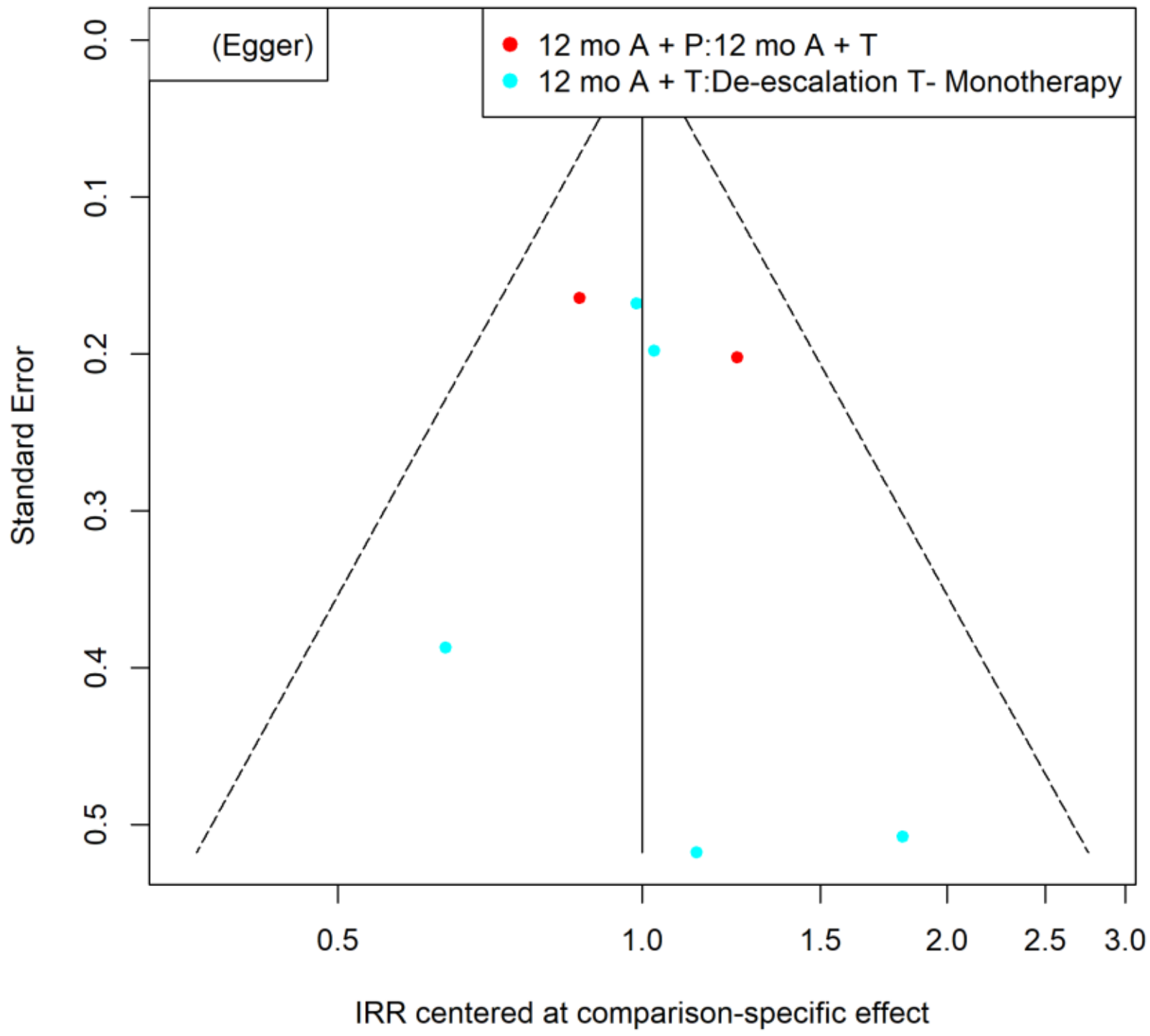

Supplementary Figure 33F. Stroke

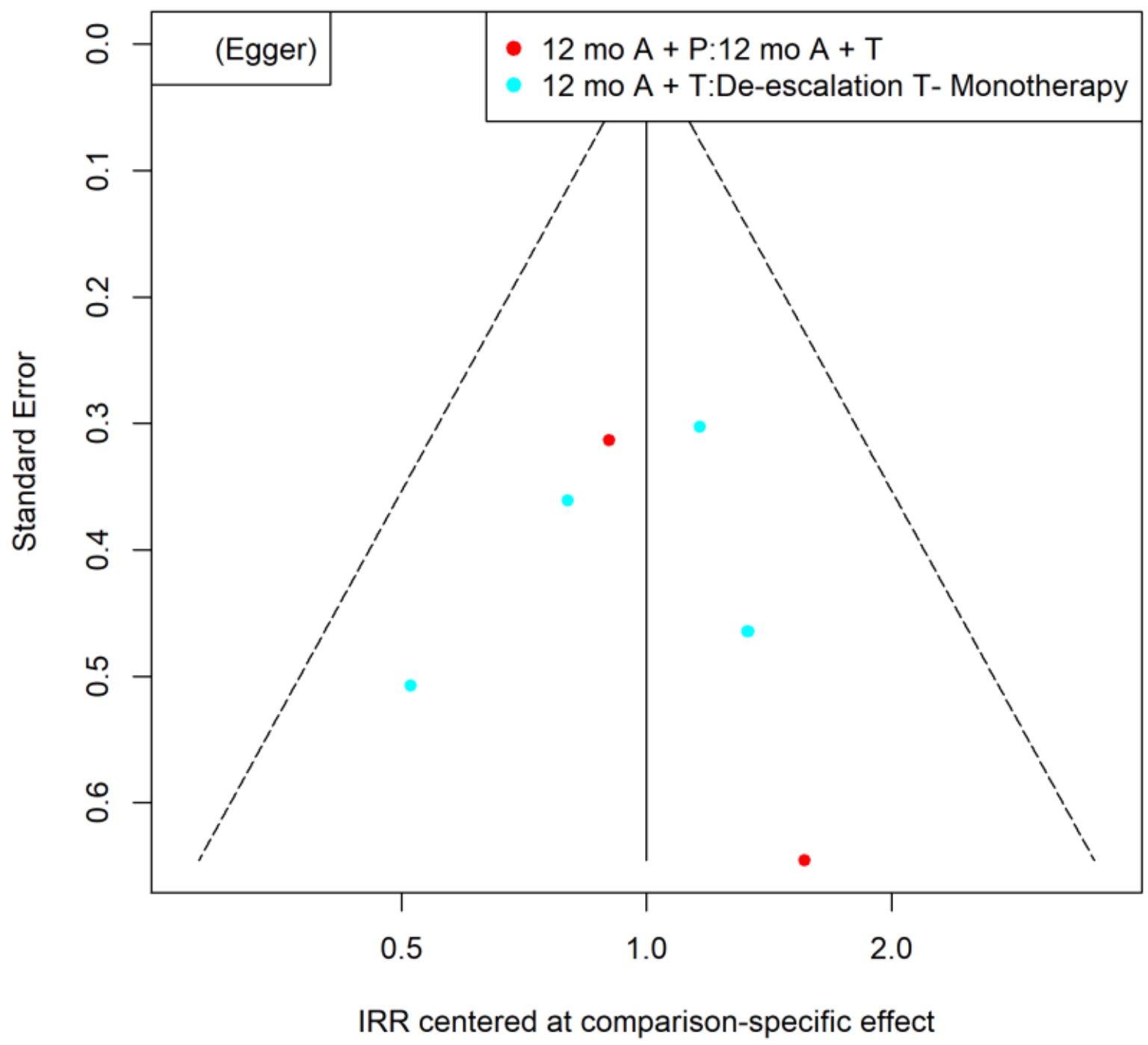

Supplementary Figure 33G. Stent thrombosis

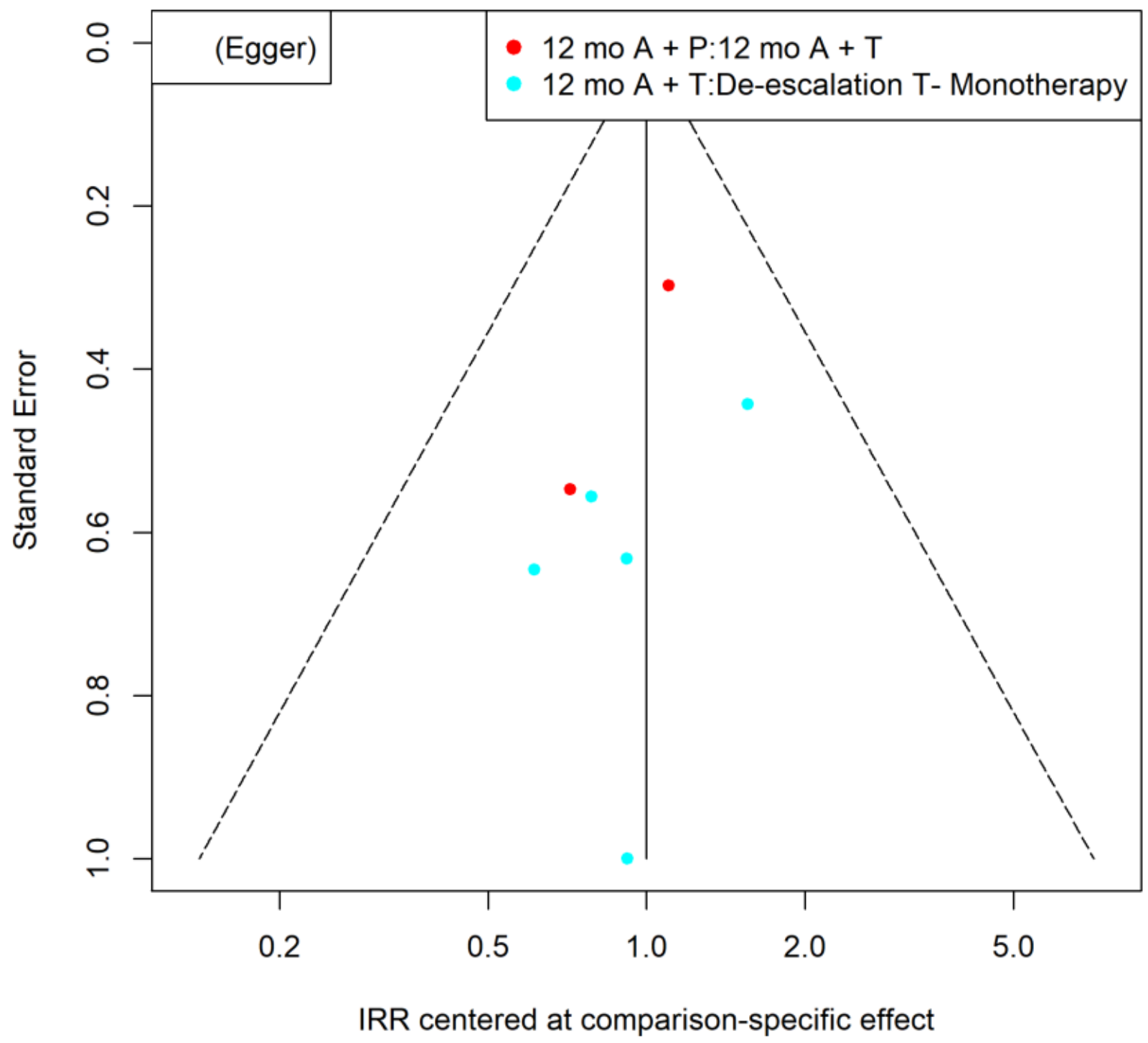

Supplementary Figure 34. RoB 2 - Cochrane tool for assessing the risk of bias in randomized clinical trials

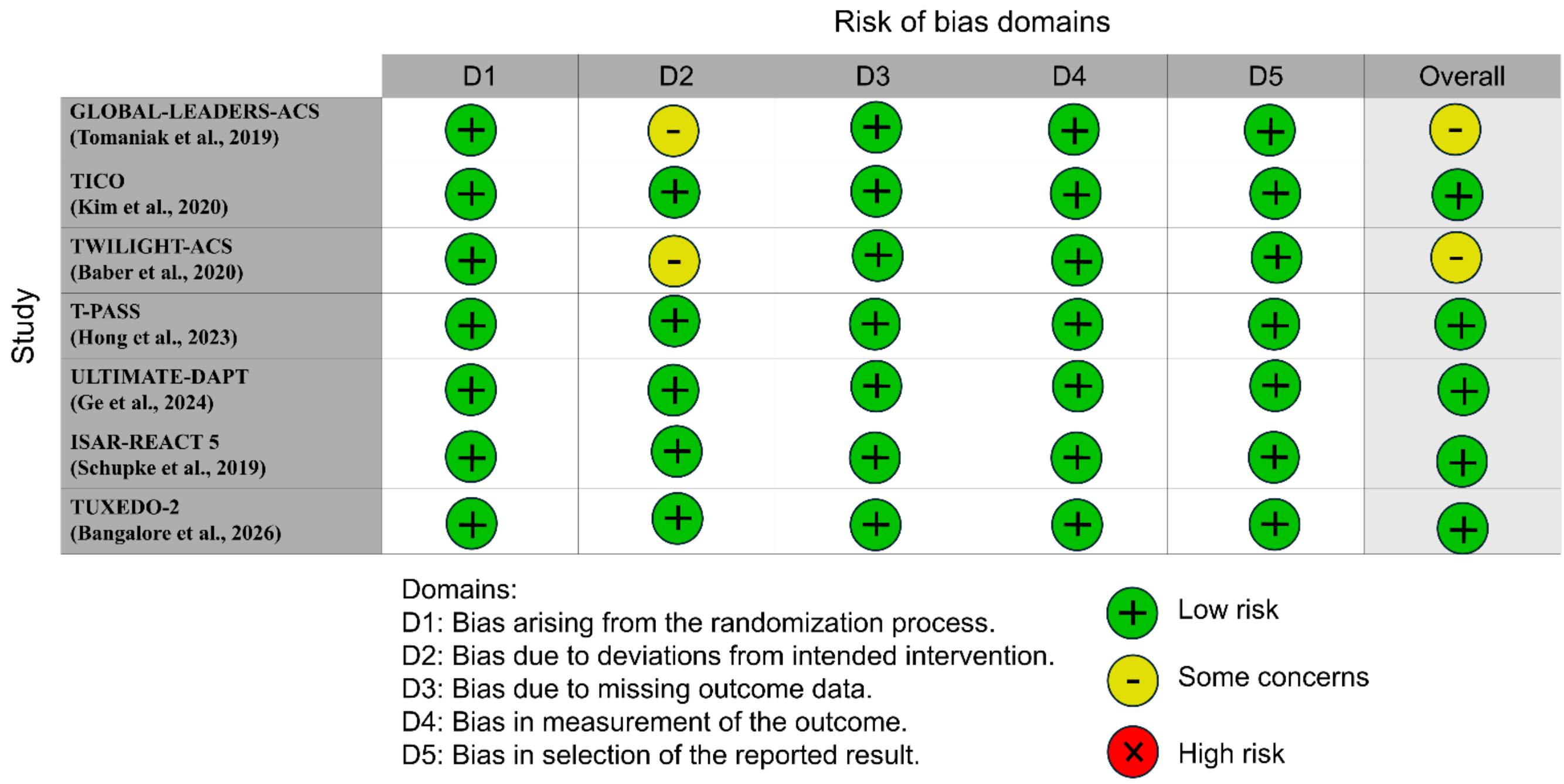

Supplementary Table 8. GRADE certainty of evidence assessment for direct estimates

Comparison	No. of studies	Risk of bias	Inconsistency	Indirectness	Publication bias	Preliminary direct rating	Imprecision	Final direct rating
**MACCE**								
**Ticagrelor monotherapy vs 12 mo A + T**	5	Not serious	Not serious	Not serious	Undetected	High	Not serious	High
**12 mo A + T vs 12 mo A + P**	2	Not serious	Not serious	Not serious	Undetected	High	Not serious	High
**Major bleeding**								
**Ticagrelor monotherapy vs 12 mo A + T**	5	Not serious	Not serious	Not serious	Undetected	High	Not serious	High
**12 mo A + T vs 12 mo A + P**	2	Not serious	Not serious	Not serious	Undetected	High	Serious	Moderate

Supplementary Table 9. GRADE certainty of evidence assessment for indirect estimates

Comparison (Treatment 1 vs. Treatment 2)	Common comparator	Treatment 1 versus Common comparator	Comparator 1 versus Comparator 2	Treatment 2 vs. Common comparator	Intransitivity	Start rating	Imprecision	Final indirect
**MACCE**								
**Ticagrelor monotherapy vs 12 mo A + P**	12 mo A + T	High	-	High	Not serious	High	Serious	Moderate
**Major bleeding**								
**Ticagrelor monotherapy vs 12 mo A + P**	12 mo A + T	High	-	Moderate	Not serious	Moderate	Not serious	Moderate

Supplementary Table 10. GRADE certainty of evidence assessment for network estimates

Comparison	Preliminary rating from direct or indirect	Incoherence	Imprecision	IRR and 95% CI for network	Final network rating	Most credible estimate
**MACCE**						
**Ticagrelor monotherapy vs 12 mo A + T**	High	**Coherence by design**	Not serious	0.85 (0.73–1.00)	High	Direct
**12 mo A + T vs 12 mo A + P**	High		Not serious	1.28 (1.07 – 1.53)	High	Direct
**Ticagrelor monotherapy vs 12 mo A + P**	Moderate		Not serious	1.09 (0.86 – 1.38)	Moderate	Network (indirect)
**Major bleeding**						
**Ticagrelor monotherapy vs 12 mo A + T**	High	**Coherence by design**	Not serious	0.47 (0.36 – 0.61)	High	Direct
**12 mo A + T vs 12 mo A + P**	High		Serious	1.14 (0.85 – 1.54)	Moderate	Direct
**Ticagrelor monotherapy vs 12 mo A + P**	Moderate		Not serious	0.54 (0.37 – 0.80)	Moderate	Network (indirect)

References

1. Tomaniak M, Chichareon P, Onuma Y, et al. Benefit and Risks of Aspirin in Addition to Ticagrelor in Acute Coronary Syndromes. *JAMA Cardiol*. 2019;4(11):1092. doi:10.1001/jamacardio.2019.3355

2. Kim BK, Hong SJ, Cho YH, et al. Effect of Ticagrelor Monotherapy vs Ticagrelor With Aspirin on Major Bleeding and Cardiovascular Events in Patients With Acute Coronary Syndrome. *JAMA*. 2020;323(23):2407. doi:10.1001/jama.2020.7580

3. Baber U, Dangas G, Angiolillo DJ, et al. Ticagrelor alone vs. ticagrelor plus aspirin following percutaneous coronary intervention in patients with non-ST-segment elevation acute coronary syndromes: TWILIGHT-ACS. *Eur Heart J*. 2020;41(37):3533-3545. doi:10.1093/eurheartj/ehaa670

4. Hong SJ, Lee SJ, Suh Y, et al. Stopping Aspirin Within 1 Month After Stenting for Ticagrelor Monotherapy in Acute Coronary Syndrome: The T-PASS Randomized Noninferiority Trial. *Circulation*. 2024;149(8):562-573. doi:10.1161/CIRCULATIONAHA.123.066943

5. Ge Z, Kan J, Gao X, et al. Ticagrelor alone versus ticagrelor plus aspirin from month 1 to month 12 after percutaneous coronary intervention in patients with acute coronary syndromes (ULTIMATE-DAPT): a randomised, placebo-controlled, double-blind clinical trial. *Lancet*. 2024;403(10439):1866-1878. doi:10.1016/S0140-6736(24)00473-2

6. Schüpke S, Neumann FJ, Menichelli M, et al. Ticagrelor or Prasugrel in Patients with Acute Coronary Syndromes. *N Engl J Med*. 2019;381(16):1524-1534. doi:10.1056/NEJMoa1908973

7. Bangalore S, Sinha SK, Singh R, et al. Ticagrelor vs Prasugrel in Patients With Diabetes and Multivessel Coronary Artery Disease. *JAMA Cardiol*. Published online February 11, 2026. doi:10.1001/jamacardio.2025.5057

8. Franzone A, McFadden E, Leonardi S, et al. Ticagrelor Alone Versus Dual Antiplatelet Therapy From 1 Month After Drug-Eluting Coronary Stenting. *J Am Coll Cardiol*. 2019;74(18):2223-2234. doi:10.1016/j.jacc.2019.08.1038

9. Mehran R, Baber U, Sharma SK, et al. Ticagrelor with or without Aspirin in High-Risk Patients after PCI. *N Engl J Med*. 2019;381(21):2032-2042. doi:10.1056/NEJMoa1908419

10. Angiolillo DJ, Baber U, Sartori S, et al. Ticagrelor With or Without Aspirin in High-Risk Patients With Diabetes Mellitus Undergoing Percutaneous Coronary Intervention. *J Am Coll Cardiol*. 2020;75(19):2403-2413. doi:10.1016/j.jacc.2020.03.008

11. Ndrepepa G, Kastrati A, Menichelli M, et al. Ticagrelor or Prasugrel in Patients With Acute Coronary Syndromes and Diabetes Mellitus. *JACC Cardiovasc Interv*. 2020;13(19):2238-2247. doi:10.1016/j.jcin.2020.07.032
